# Supplementary material for: Trichoderma reesei Contains a Biosynthetic Gene Cluster That Encodes the Antifungal Agent Ilicicolin H
Source: J Fungi (Basel). 2021 Dec 1;7(12):1034. doi: 10.3390/jof7121034 (PMC8705728; doi:10.3390/jof7121034)
Supplement: Supplementary file 1 [file jof-07-01034-s001.zip › jof-1477853-supplementary.pdf]

Journal of Fungi

MDPI

Article

Trichoderma reesei contains a biosynthetic gene cluster that encodes the Antifungal Agent Ilicicolin H

Mary L. Shenouda [1,2], Maria Ambilika [1] and Russell J. Cox [1,*]

1 Institute for Organic Chemistry and BMWZ, Schneiderberg 38, 30167, Hannover, Germany.
2 Pharmacognosy Department, Faculty of Pharmacy, Alexandria University, 21521, Egypt.
* Correspondence: russell.cox@oci.uni-hannover.de

Electronic Supplementary Information

Contents

1. **Growth media, buffers and solutions, enzymes and antibiotics** 2
2. **Bacterial and fungal strains and microbiological methods** 4
3. **Vectors and oligonucleotides** 7
4. **LCMS Programmes** 8
5. **Bioinformatic Analyses** 9
6. **Structure Elucidation** 11
7. **Tabulated NMR Data** 14
8. **Selected NMR spectra** 20
9. **HRMS data** 45
10. **References** 48

1. Growth media, buffers and solutions, enzymes and antibiotics

Table S1.1: Growth media:

Media	Composition [% (*w/v*)]	Ingredients
**LB**	0.50	Yeast extract
	1.00	Tryptone
	0.50	NaCl
**SOC**	93.75 (*v/v*)	SOB
	1.25 (*v/v*)	$MgCl_2 \times 6\ H_2O$
	5.00 (*v/v*)	D(+)-glucose 20 %
**YPAD**	1	Yeast extract
	2	Tryptone
	2	D(+)-Glucose Monohydrate
	0.03	Adenine
DPY	2.00	Dextrin from potato starch
	1.00	Polypeptone
	0.5	Yeast extract
	0.5	KH_2PO_4
	0.05	$MgSO_4.6(H_2O)$

Table S1.2: Solid media:

Agar	Composition [% (*w/v*)]	Ingredients
**SM-URA**	0.17	Yeast nitrogen base
	0.50	$(NH_4)_2SO_4$
	2.00	D(+)-Glucose Monohydrate
	0.077	Complete supplement mixture minus Uracil
	2.50	Agar
**LB agar**	0.5	Yeast extract
	1.00	Tryptone
	0.5	NaCl
	1.5	Agar
**YPAD agar**	1.00	Yeast extract
	2.00	Tryptone
	2.00	D(+)-Glucose Monohydrate
	0.03	Adenine
	1.50	Agar
**CZD/S agar**	3.50	Czapek Dox broth
	18.22	D-sorbitol (1M)
	0.10	$(NH_4)_2SO_4$
	0.05	Adenine
	0.15	L-Methionine
	1.50	Agar
**CZD/S soft agar**	3.50	Czapek Dox broth
	18.22	D-sorbitol (1M)
	0.10	$(NH_4)_2SO_4$
	0.05	Adenine
	0.15	L-Methionine
	0.80	Agar
**CD1 agar**	3.50	Czapek Dox broth
	4.68	NaCl (0.8 M)
	0.10	$(NH_4)_2SO_4$
	0.05	Adenine
	0.15	L-Methionine
	1.50	Agar
**DPY agar**	2.00	Dextrin from potato starch
	1.00	Polypeptone
	0.5	Yeast extract
	0.5	KH_2PO_4
	0.05	$MgSO_4.\ 6(H_2O)$
	2.50	Agar

Table S1.3: Solutions:

Solutions	Components
**Protoplasting solution for *A. oryzae***	0.8 M NaCl, 10 mg/mL T*richoderma harzianum* lysing enzyme
**Transformation solution I for *A. oryzae***	0.8 M NaCl, 10 mM $CaCl_2$, 50 mM Tris-HCl, pH 7.5
**Transformation solution II for *A. oryzae***	0.8 M NaCl, 10 mM $CaCl_2$, 50 mM Tris-HCl, pH 7.5, 60 % (*w/v*) PEG3350
**10 x TE buffer**	100 mM Tris HCl pH 8, 10 mM EDTA
**Loading-dye (6 x)**	0.25 % (*w/v*) bromophenol blue, 30 % (*v/v*) glycerine, 0.25 % (w/v) xylene cyanol
**50 X TAE buffer**	2 M Tris acetate, 0.05 M EDTA, pH 8.3

Table S1.4: Enzymes:

Name	Function	Reference
**AscI**	Restriction enzyme	NEB
**NotI**	Restriction enzyme	NEB
**Gateway™ LR Clonase™ II Enzyme Mix**	contains integrase, excisionase and integration host factor	Thermo Fisher Scientific
**OneTaq® 2X Master Mix with Standard Buffer**	DNA polymerase with agarose gel running buffer	NEB
**Proteinase K**	Proteinase	Thermo Fisher Scientific
**Q5® High-Fidelity DNA Polymerase 2x Master Mix**	DNA polymerase with enhanced proofreading ability	NEB
***Trichoderma harzianum* lysing enzyme**	Cell Dissociation and cell lysis; preparation of protoplasts	Sigma
Driselase	Cell Dissociation and cell lysis; preparation of protoplasts	Sigma

Table S1.5: Antibiotics:

Name	Stock concentration / mg/mL in H_2O	Final concentration / µg/mL
Carbenicillin disodium salt (Carb)	50	50
Kanamycin (Kan)	50	50

2. Bacterial and Fungal Strains and microbiological methods

Table S2.1: Bacterial and fungal strains:

Strain	Genotype	Origin
E. coli Top10	F-*mcrA* Δ(*mrr-hsd*RMS-*mcr*BC) Φ80*lac*ZΔM15 Δ*lac*X74 *rec*A1 *ara*D139 Δ(*araleu*)7697 *gal*U *gal*K *rps*L(Str^R) *end*A1 *nup*G	Thermo Fisher Scientific
E. coli ccdB survival cells	F-*mcrA* Δ(*mrr-hsd*RMS-*mcr*BC) Φ80*lac*ZΔM15 Δ*lac*X74 *rec*A1 *ara*Δ139 Δ(*ara-leu*)7697 *gal*U *gal*K *rps*L (Str^R) *end*A1 *nup*G *fhu*A::IS2	Thermo Fisher Scientific
S. cerevisiae CEN.PK2	*MATa/α ura3-52/ura3-52 trp1-289/trp1-289 leu2-3_112/leu2-3_112 his3 Δ1/his3 Δ1MAL2-8C/MAL2-8C SUC2/SUC2*	Hahn group, Hannover
A. oryzae NSAR1	Δ*niaD*, Δ*sC*, Δ*adeA*, Δ*argB*	Lazarus group, Bristol [1,2]
T. reesei* QM6a Δ*tmus53* Δ*pyr4	Δ*tmus53* Δ*pyr4*	Mach-Aigner group [3]

2.01 Protocol for Escherischia coli transformation

E. coli strains were cultivated on liquid and solid LB medium with the approperiate antibiotic. Cultures were incubated at 37 °C and 200 rpm for 16-24 hours. Glycerol stocks (25% glycerol) were stored at -80 °C for long term storage of the strains. Competent *E. coli* cells stored at -80 °C were thawed on ice for 5 min. 1-10 µL vector DNA were added and cells were stored on ice for 20-30 min. Heat shock was conducted at 42 °C for 30-45 s and cells were immediately placed on ice for 2 min. 250 µL SOC medium were then added and samples were incubated at 37 °C with 300 rpm shaking for 1 h. The cells were spun down for 15 s and 250 µL of the supernatant was discarded. The cells were then suspended in the remaining supernatant and spread on LB agar plates containing the appropriate antibiotic and incubated over night at 37 °C.

2.02 Protocol for Saccharomyces cerevisiae transformation

S. cerevisiae strain was cultivated on YPAD agar and incubated at 30 °C for three to five days. A single colony was used to inoculate 10 ml liquid YPAD media and was then grown overnight at 30 °C and 200 rpm. After transformation with *ura3* containing plasmids, *S. cerevisiae* strains was grown on SM-Ura agar plates at 30 °C for three to five days. The high efficiency version of LiAc/SS carrier DNA/PEG protocol developed by Gietz and Schiestl [4] was used for the preparation and transformation of competent *S. cerevisiae* cell. A single colony was transferred into 10 mL YPAD medium and incubated over night at 30 °C with 200 rpm shaking. This starter culture was added to 40 mL YPAD medium in a 250 mL Erlenmeyer flask and incubated for another 4.5 h at 30 °C with 200 rpm shaking. Culture was harvested by centrifuging at 3.000 × g for 5 min. The cell pellet was first washed with 25 mL double-distilled (dd) H_2O before it was resuspended in 1 ml dd.H_2O, transferred into a 1.5 mL reaction tube and centrifuged at 13.000 × g for 30 s and supernatant discarded. The pellet was suspended in 400 µL water and each 100 µL were transferred into a separate 1.5 mL reaction tube.

For yeast recombination based cloning using *S. cerevisiae*, 100 µL aliquots of yeast competent cells were centrifuged for 30 s at 13.000 × g and the supernatant was discarded. The following components were added to the pellet in order: 240 µL PEG solution (50 % (*w/v*) polyethylene glycol 3350), 36 µL LiAc (1 M), 50 µL denaturated salmon testis DNA (2 mg.mL^{-1}), 34 µL DNA containing the linearized plasmid and desired inserts obtained by PCR in equimolar concentration. The PCR fragments contain each 30 bp overlap at both 5` and 3` with the cut sites of the vector fragments to facilitate homologous recombination. The uncut plasmid was used for positive and linearized plasmid was used for negative control. The pellet was resuspended in the transformation mix by vigorous vortexing and incubated for 40 min at 42 °C. Cells were pelleted by centrifugation at 13.000 × g for 30 s and supernatant was removed. The pellet was resuspended in 500 µL dd.H_2O and each 250 µL were spread on selective SM-Ura plates, which were incubated for

three to four days at 30 °C. Yeast plasmid was extracted using a Zymoprep™ Yeast Plasmid Miniprep II kit (Zymo reserach, Orange, California, USA).

2.03 Protocol for Aspergillus oryzae NSAR1 transformation

A. oryzae NSAR1 [1,2] was grown on DPY agar at 28 °C. For glycerol stocks 5 ml of dd.H_2O were added to the plate and spores were scraped off using a sterile woody spatula. 800 µl of spore solution were supplemented with 800 µl glycerol (50 %) and stored in a cryovial at -80 °C. For liquid cultures 100 ml DPY medium were inoculated with *A. oryzae* spore solution and incubated at 28 °C for two to seven days with 110 rpm shaking.

A. oryzae NSAR1 conidia from sporulating plates were inoculated into 100 ml DPY media and incubated overnight at 28 °C with shaking at 110 rpm. Germinated conidia were collected by filtrating with the sterile Miracloth and the pellet was resuspended in 15 mL of filter-sterilized protoplasting solution (10 mg/mL *Trichoderma* lysing enzyme in 0.8 M sodium chloride). The conidia was then incubated with the protoplasting solution for three hours at 28 °C with gentle mixing. The protoplasts were released from hyphal strands by gentle pipetting with wide-bore pipette and filtered through sterile Miracloth. The filtrate was then centrifuged at 3000 × g for 5 min to pellet the protoplasts which were resuspended in solution 1 (100 µl per transformation) and the concentration of the protoplasts was assessed microscopically. 10 µl of the plasmid DNA was added to 100 µl of the protoplast suspension and incubated on ice for 2 min. Then 1 ml of solution 2 was added and the transformation mixture was incubated at room temperature for 20 min. 5 ml of molten CZD/S soft agar at 50 °C was added to the transformation mixture and overlaid onto prepared CZD/S and the plates were incubated at 28 °C for 3-5 days. For selection on arginine–deficient media, the selection has to be repeated twice to prevent false positives due to consuming arginine from dead cell material. A few spores of the grown colony were picked with a tooth pick and transferred to a CD1 plate. The plates were incubated at 28 °C for 3–4 days and the step was repeated by streaking out single colonies. Finally, a single colony was grown on DPY agar for one week at 28 °C and 110 rpm before fermentation.

2.04 Molecular Biology Methods

All enzymes used were purchased from NEB and Thermo Fisher scientific and was used according to the manufacturer′s instructions using the supplied buffers.

2.05 Plasmid DNA Extraction from E. coli

Plasmid DNA was generally extracted from overnight culture of *E. coli* using the NucleoSpin® Plasmid Kit (Machery-Nagel), according to the manufacture′s protocol. The plasmid DNA concentration of each isolation was measured using Nanodrop spectrophotometer.

2.06 Plasmid DNA Extraction from S. cerevisiae

Plasmid DNA was isolated from the 2-3 - days culture of *S. cerevisiae* using Zymoprep™ Yeast Plasmid Miniprep II Kit (Zymo Research, USA), according to the manufacture′s protocol. All the colonies grown on one SM-URA agar plate after the yeast recombination were collected with a tooth pick and dissolved in 200 µl of solution 1 of the yeast miniprep kit. Then yeast DNA was isolated according to manufacturers′ instructions and 10 µl dd.H_2O was used for the final elution. Generally, 3-10 µl of the isolated plasmid was transformed immediately to competent *E. coli* cells.

2.07 Fungal Genomic DNA extraction

Fungal genomic DNA was isolated from about 100 mg of mycelia using the GeneElute™ Plant Genomic DNA Miniprep Kit (Sigma-Aldrich, Germany) according to the manufacture′s protocols.

2.08 Polymerase Chain Reaction (PCR)

PCR was used to amplify DNA fragments for both screening and cloning purposes. OneTaq® 2 × Master Mix (NEB) was used for screening purposes, while Q5® High–Fidelity 2 × Master Mix (NEB) was used for precise fragment amplification for cloning purposes. Both enzymes were used according to the manufacture′s protocols.

2.09 Cloning Procedure

All of restriction enzymes were purchased from NEB. The enzymatic reactions were conducted according to restriction digest protocols. The digested plasmids were then evaluated by agarose gel electrophoresis. Invitrogen Gateway® LR Clonase II enzyme mix was used to perform *in vitro* recombination between pE-YA entry plasmids and pTYGS expression plasmids [2]. The enzyme was used according to the manufacturer's protocol with reduced reaction scale (reduced to half). DNA samples were sequenced by Eurofins Genomics (Ebersberg, Germany). 2 µL of 10 µM primer solution was added to templates consisting of at least 15 µg of purified DNA. The mixture was then sent for sequencing.

2.10 Agarose Gel Electrophoresis

Agarose gel electrophoresis was used to analyse DNA fragments and Rota safe DNA stain was used to visualize the DNA fragments. Generally, 1 % agarose solution was prepared by dissolving 1g Agarose in 100 mL of 0.5 × TAE buffer. DNA samples were mixed with 6 × DNA Loading Dye and loaded on 1% (*w/v*) agarose gels. 1 kb DNA Ladder (NEB) was used as a molecular DNA size marker. Electrophoresis was carried out in horizontal gel tanks (BioRad) at 90–110 V for 20-35 min using 0.5 × TAE as running buffer. DNA was visualised under UV (254 nm) and photographed using Gel Doc™ XR+ Gel Documentation System (BioRad).

2.11 DNA recovery from PCR

NucleoSpin® Gel and PCR Clean–up Kit was used to directly purify PCR mixture according to the manufacture's protocol. The isolated DNA fragments were then evaluated by agarose gel electrophoresis.

2.12 E. coli colony PCR

To screen positive colonies of *E. coli*, part of each single colony was picked from the LB agar plate using a toothpick and dipped into 10 µl TE buffer in PCR tube. The cells were then disrupted by heating to 80 °C for 15 minutes followed by 55 °C for another 15 min using a thermocycler. Then 1 µl of the cell suspension was used for PCR (using OneTaq® 2 × Master Mix, NEB), according to the manufacturers protocol.

3. Vectors and Oligonucleotides

Table S3.1. Vectors used in this work:

Name	Origin
pTYGS-*argB*	Lazarus group, Bristol [2]
pE-YA	Lazarus group, Bristol [2]

Table S3.2. Vectors constructed in this work

vector	ID	Vector backbone	Oligonucleotides for construction in *S. cerevisiae.*
**pE-YA-*triliA***	MSIII139	pE-YA	P_{a+b}: 1543+ 1544<br>P_{c+d}: 1545 +1546<br>P_{e+f}: 1547 + 1548<br>P_{g+h}: 1549 + 1550
**pTYGS-*argB-triliA***	MSIII144	pTYGS-*argB*	LR with MSIII139
**pTYGS-*argB-triliA-triliB***	MSIII152	MSIII144	ER-58289: 1551 + 1552<br>Patch adh: 89 + 90<br>Patch Peno: 87 + 88
**pTYGS-*argB-triliA-triliB-triliC***	MSIII165-A	MSIII144	ER-58289: 1551 + 1552<br>P450-58953: 1553 + 1554<br>Patch Peno: 87 + 88
**pTYGS-*argB-triliA-triliB-triliC-triliD***	MSIII165-B	MSIII144	ER-58289: 1551 + 1552<br>P450-58953: 1553 + 1554<br>C-MT: 1555 + 1556

Table S3.3 Primers and oligonucleotides:

NO.	Name	sequence
**87**	Peno plugF	CTTCTTAAATATCGTTGTAACTGTTCCTGA
**88**	Peno plugR	CGAAGTATATTGGGAGACTATAGCTACTAG
**89**	Padh plugF	ATTCACCACTATTATTCCCACCCTATAATA
**90**	Padh plugR	GAGACGAAACAGACTTTTTCATCGCTAAAA
**1543**	IlliA-P1	TAATGCCAACTTTGTACAAAAAAGCAGGCTATGGATCAACAAAGACAGCA
**1544**	IlliA- P2	ACACCTTGTCGTGGTCCCACGAGTATAAAGGCAATCCCTTGGGGATTTTG
**1545**	IlliA- P3	CTTTATACTCGTGGGACCACGACAAGGTGTTCTGGCGCGAGGGACGTCTA
**1546**	IlliA- P4	TCTCCGGGCTGCAATCTTGCTCATCAATGGGGATGACGTGTGTGCCATGA
**1547**	IlliA- P5	CCATTGATGAGCAAGATTGCAGCCCGGAGAGGCTCTCTGCTATTGCAAAT
**1548**	IlliA- P6	CCAGAACAACGCCCAGCCCAATGACATCCATGACAATGTGATGGTATGCG
**1549**	IlliA- P7	TGGATGTCATTGGGCTGGGCGTTGTTCTGGACGACTTGAACAATGCTTAC
**1550**	IlliA- P8	TATAATGCCAACTTTGTACAAGAAAGCTGGTTAATCGCTTCCCATATAAG
**1551**	ER-58289-gpdA-PF	ACAGCTACCCCGCTTGAGCAGACATCACCGATGACTGTCATTGATGTGCT
**1552**	ER-58289-gpdA-PR	TACGACAATGTCCATATCATCAATCATGACTCAAGCCGATACAGCAATGG
**1553**	P450-58953-adh-PR	TTTCATTCTATGCGTTATGAACATGTTCCCTCACTTGTAGAAAGCTATCT
**1554**	PF-long P450-adh	TTCTTTCAACACAAGATCCCAAAGTCAAAGATGTTGTTGCCCGAGTACGT
**1555**	PF C-MT eno	CGACTGACCAATTCCGCAGCTCGTCAAAGGATGTCTTCTACTCAAACAAC
**1556**	PR C-MT eno	CAGGTTGGCTGGTAGACGTCATATAATCATCTACTTGCTTTTGTGGTCTT
**1704**	Check illiA PF	CCGAGTTCGAGACGTGGGCT
**1705**	Check illiA PR	CCACCAGTACCGGCTCCGAT

4. LCMS Programmes

Table S41. Program used for analytical LCMS:

Program	Time/min	Water-acetonitrile / %
Standard	0-1<br>1-10<br>10-12<br>12-13<br>13-15	90-10<br>10-90<br>10-90<br>90-10<br>90-10

5. Bioinformatic Analysis

5.1 Search for all PKS and PKS-NRPS genes

Bioinformatic analysis was done using a manual search strategy based on the Blastp algorithm using a template protein sequence of known non-reducing PKS (AMS38559.1) from *Penicillium chrysogenum* against the protein database of *T. reesei* QM6a. The protein database for *T. reesei* QM6a was downloaded from NCBI (Refseq assembly accession number GCF_000167675.1, V2.0). Blastp search was done using Geneious Prime 2021.2.2 using standard parameters (BLOSUM62 matrix, Max E-value 0.05, gap cost 11 1). The Blastp search resulted in 33 hits representing 21 different proteins. Careful inspection of the conserved domains on NCBI of 21 proteins showed only 13 proteins to have the minimum catalytic domains for PKS (KS and AT domains), which include 11 PKS genes and two hybrid PKS-NRPS genes (Figure S5.1).

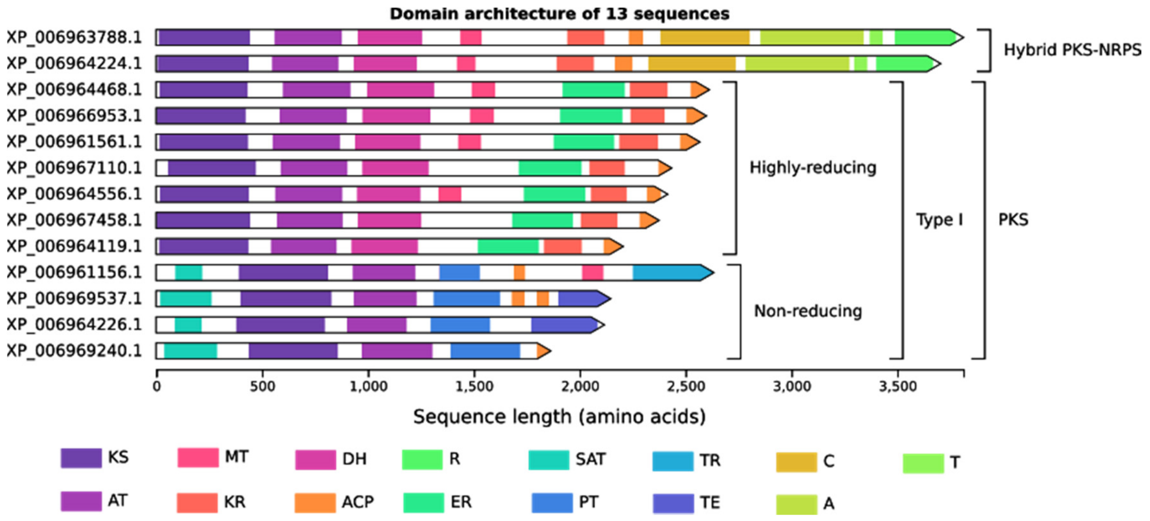

Figure S5.1 Domain architecture of all the predicted PKS and hybrid PKS-NRPS from *T. reesei* represented by synthaser software.[5]

5.2 PKS and hybrid PKS-NRPS in T. reesei

Table S5.1 A list of all PKS and hybrid PKS-NRPS in *T. reesei* genome:

	Protein accession No.	Gene No. (TRIREDRAFT)	Function	Length of the protein (aa)	Closest hit by Blastp [6] (% identity at protein level, swissprot database)	Ref.
**1**	XP_006961561	73618	hr-PKS	2567	SorA (100%)	[7,8]
**2**	XP_006964119	59482	hr-PKS	2205	T-toxin PKS (34.6%)	[9]
**3**	XP_006964224	59315	PKS-NRPS	3704	Fusarin C (61.2%)	[10]
**4**	XP_006964468	106272	hr-PKS	2612	Alternapyrone (32.6%)	[11]
**5**	XP_006964556	60118	hr-PKS	2415	Beauveriolides (*cm3B*, 33.6 %)	[12]
**6**	XP_006966953	65172	hr-PKS	2598	6-hydroxymellein (*cdmE*, 36%) Ochratoxin (37%)	[13,14]
**7**	XP_006967458	65891	hr-PKS	2374	Radicicol (*radS1*, 32%)	[15]

**8**	XP_006963788	58285	PKS-NRPS	3812	Illicicolin-H (*iliA*, 66.4%)	[16]
**9**	XP_006967110	65116	hr-PKS	2434	Trichoxide Vir A (87.1 %)	[17]
**10**	XP_006969537	82208	nr-PKS	2146	conidial pigment synthase (76%)	[18]
**11**	XP_006961156	73621	nr-PKS	2633	SorB (100%)	[7,8]
**12**	XP_006969240	81964	nr-PKS	1863	PkgA (67.1 %)	[19]
**13**	XP_006964226	105804	nr-PKS	2116	(AscC) ascofuranone/ascochlorin BGC	[20]

5.3 cblaster search using the reannotated Trili-PKS/NRPS BGC

The re-annotated *T. reesei* PKS/NRPS BGC was then used to identify homologous gene clusters from other fungal species using cblaster [21] (Figure S5.2). The results showed a highly similar BGC present in some other *Trichoderma* spp. such as the biocontrol fungus *Trichoderma virens* GV29-8, although ilicicolin-H was not reported before from any *Trichoderma* spp. [22]

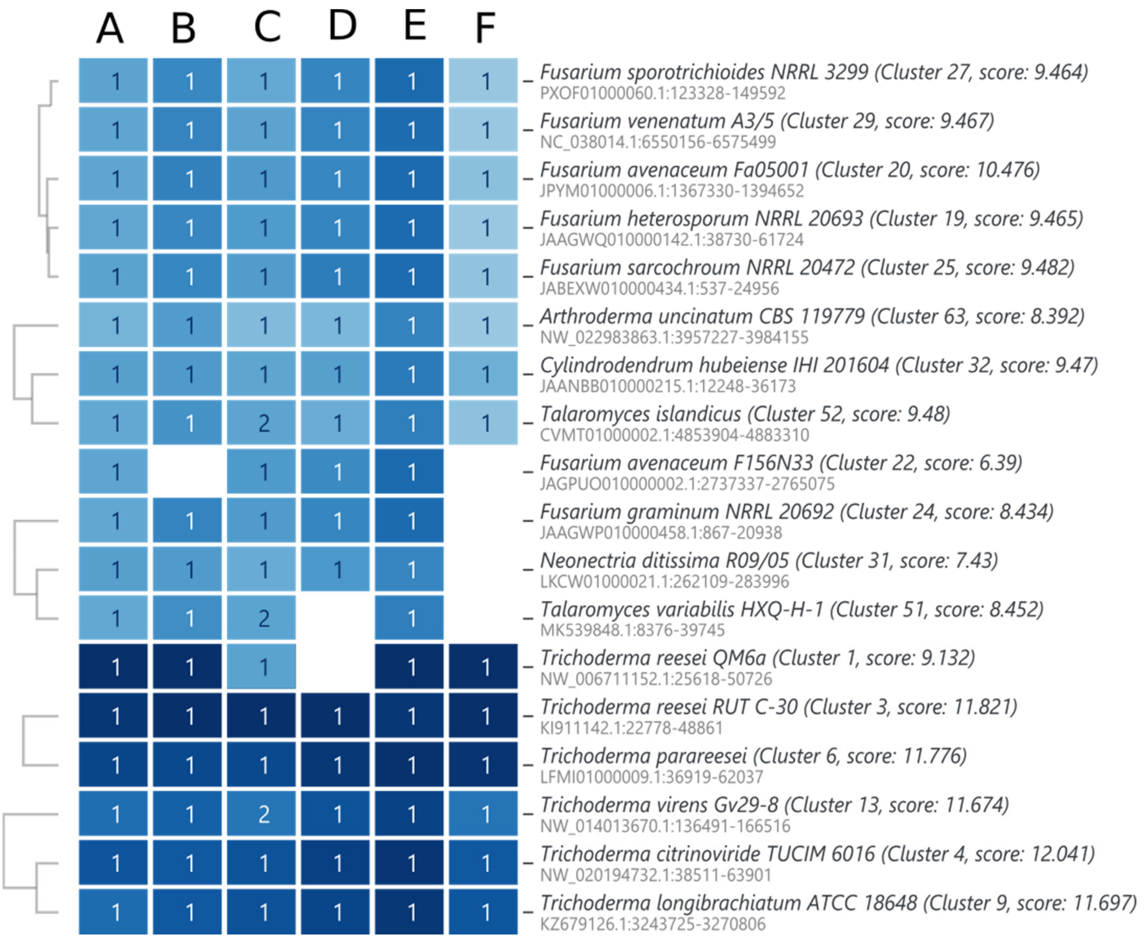

Figure S5.2 cblaster results for clusters from other fungal species that are closely related to the identified Trili-PKS-NRPS cluster from *T. reesei*, Gene codes: **A**, PKS-NRPS; **B**, ER; **C**, oxidoreductase; **D**, *C*-MeT; **E**, P450; **F**, TF.

6. Structure Elucidation

6.1 Coexpression of triliA and triliB

A. oryzae pTYGS·*argB·triliA·triliB colony* B was cultivated in 10 x 100 ml DPY media for six days. The crude extract was then submitted to preparative LCMS and 30 mg of the major compound **11** (T_R = 6.7 min) and 4 mg of the minor compound **12** (T_R = 8.05 min) were isolated. However, isolation of compound **13** (T_R = 11.0 min) was not possible due to the very low concentration of the compound in the extract.

Compound 11

HRMS analysis of the major compound **11** confirmed a molecular formula of $C_{23}H_{28}NO_6$ ([M] + H^+ calculated 414.1917, found 414.1917). ^{13}C-NMR analysis of this compound in deuterated methanol showed the presence of only 18 carbon resonances corresponding to 20 carbon atoms, suggesting that three of the expected carbon resonances may be too weak to be directly observed. Eight peaks were in the range of 114-156 ppm indicating aromatic or olefinic carbons.

HSQC analysis of compound **11** showed that the two ^{13}C resonances at 116.1 and 131.8 ppm correspond to the two proton doublet signals at 6.67 and 6.99 ppm respectively, each integrating to two protons. This indicated the presence of a *para*-substituted benzene ring. HSQC spectra also showed the presence of two methyl carbons at 12.1 and 19.9 ppm, four methylene carbons at 27.7, 36.8, 37.7 and 42.4 ppm, five methine carbons at 31.3, 64.0, 116.7, 146.7 and 150.7 ppm and five quaternary carbons at 127.8, 135.6, 157.3, 176.2 and 176.9 ppm.

Proton signals for the hydroxyl groups are missing, presumably due to proton exchange in the deuterated solvent methanol. This can also explain the missing signals for three quaternary carbons in the ^{13}C-NMR spectrum as ^{2}H-exchange in deuterated solvent can result in delayed relaxation in the ^{13}C-NMR and therefore, very broad signals that are subsumed by baseline noise. To identify the missing carbon signals and hydroxyl protons, the deuterated methanol was evaporated and re-protonation of compound **11** was done by dissolving the compound in pure methanol and evaporation several times. Finally, 7 mg of the pure compound was dissolved in deuterated DMSO-d_6 and submitted for NMR analysis. ^{13}C-NMR spectrum of compound **11** in DMSO-d_6 showed the presence of 21 peaks corresponding to 23 carbon atoms (Figure S8.7).

Although the ^{1}H-NMR spectrum of the compound in DMSO-d_6 showed the missing hydroxyl groups, some proton signals were poorly separated and overlapped with the water and solvent peaks (Figure S8.6). Therefore, the data obtained from the two solvents were used together to fully elucidate the structure (Table S7.1). The ^{1}H-^{1}H COSY spectrum revealed the coupling between the methylene protons (H-6a at 2.87 ppm and H-6b at 3.00 ppm) and the methine proton (H-5 at 4.08 ppm, Figure S8.11-A). Based on that and the HMBC correlations (Figure S8.11 B-D), it was evident that the structure has a tetramic acid moiety. This was supported by an observed UV_{max} of 360 nm typical for this class of compound.

The side chain of the structure showed signals for four carbons in the olefin range; three methine carbons (at 116.7, 150.7 and 146.7) and one quaternary carbon at 135.6 ppm. The olefin at C-8/C-9 was shown to be *trans*, as the corresponding doublets at 7.10 and 7.50 ppm had the same coupling constant of 15.6 Hz. Based on COSY and HMBC correlations, the rest of the structure was elucidated (Figure S8.12). Notably, although the signal for the methyl group

(H-18 at 1.89 ppm) is an apparent singlet, it demonstrated a coupling to the olefin hydrogen (H-11 at 6.11 ppm) which is 4-bonds away that might be attributed to the W-coupling between these protons.

Compound 12

The chemical structure of the minor compound with nominal mass of 465 (T_R = 8.05 min) was identified based on HRMS, 1D and 2D NMR data. HRMS analysis of the minor compound **12** confirmed a molecular formula of $C_{27}H_{32}NO_6$ ([M] + H^+ calculated 466.2230, found 466.2237). When compared to the major compound **11**, the NMR signals for compound **12** in the tetramic acid region is quite similar. Additionally the uv_{max} of 360 nm also supported this conclusion. However, the rest of the structure showed some differences in the NMR data. For the side chain of the structure, the ^{1}H-NMR spectrum exhibited eight methines (including three vinylic), three methylene and two methyl groups. Based on 1D and 2D NMR, the compound was identified to be compound **12**, which is the oxidized Diels-Alder product of the ilicicolin-H tetramic acid (Table S7.2). The side chain of compound **12** showed a number of COSY correlations (Figure S8.18 A-C) proving the predicted decalin structure. The predicted decalin structure was also confirmed by HMBC correlations (Figure S8.19). This indicated that the formation of the decalin rings could occur spontaneously by intramolecular Diels-Alder reaction. The rest of the structure was also confirmed by HMBC correlations (Figure S8.20).

However, the major compound **11** lacks a double bond at the end of the side chain that could interact with the double bond at C-8/C-9 to form this kind of decalin structure. This might indicate the formation of a compound with longer side chain similar to that of ilicicolin-H tetramic acid. This compound could then form the new compound **12** by intramolecular Diels-Alder reaction followed by oxidation of the methyl group at the end of the carbon chain. Also the fact that the major product **11** of the megasynthase has very similar chemical structure to the ilicicolin-H tetramic acid **13** with only four carbon less was quiet intriguing, especially with the unsaturation at the end of the chain that makes the structure vulnerable to oxidation.

5.2 *Coexpression of triliA, triliB and triliC.*

For the structure elucidation of the new compounds, the producing strain *A. oryzae* pTYGS·*argB·triliA·triliB·triliC* was cultivated in 1 L DPY media for six days at 28 °C and 110 rpm. The crude extract was then submitted to preparative LCMS and 1 mg of each of the two compounds with nominal mass 433 and 411 were purified. The two isolated compounds were then submitted to HRMS, 1 and 2D NMR analysis.

Compound 14

HRMS analysis of compound **14** with nominal mass 411 confirmed a molecular formula of $C_{23}H_{24}NO_6$ ([M] - H^+ calculated 410.1604, found 410.1608). The molecular weight of this compound is two mass units less than compound **11**, which might indicate a structure with 2-pyridone ring instead of tetramic acid ring due to oxidative ring expansion through the action of the expressed P450 enzyme. Additionally, uv_{max} of 340 nm is typical of acyl 2-pyridones rather than acyl tetramic acids (uv_{max} 360 nm). The structure of the compound **14** was elucidated based on 1 and 2D NMR. ^{13}C- and ^{1}H-NMR signals for the side chain of the compound were comparable to those of the tetramic acid compound **11** in

deuterated methanol but showed some down-field chemical shift that could be attributed to the conjugation with the phenyl-2-pyridone rings. ^{1}H-NMR of compound **14** showed three signals in the aromatic range 6.8-7.5 ppm corresponding to five hydrogens. The singlet peak at 7.47 ppm corresponding to H-6 showed no COSY correlations (Figure S8.26-B) and HMBC correlations to two carbonyl carbons at 164.5 and 178.1 ppm and to a quaternary carbon at 125.4 (Figure S8.27-B). This indicated the expansion of the tetramic acid 5-membered ring to the 2-pyridone 6-membered ring. The rest of the structure was confirmed by COSY and HMBC correlations.

Compound 15

HRMS analysis of compound **15** with nominal mass 433 confirmed a molecular formula of $C_{27}H_{30}NO_4$ ([M] - H^+ calculated 432.2175, found 432.2175). ^{13}C- and ^{1}H-NMR data of this compound in deuterated acetone was identical to that reported for compound **15** from the ilicicolin-H BGC (Table S7.4).[23]

5.3 *Coexpression of triliA, triliB, triliC and triliD.*

Transformation of *A. oryzae* NSAR1 was done with pTYGS·*argB·triliA·triliB·triliC·triliD* PEG-mediated transformation protocol and resulted in the generation of six transformants with the four genes (*triliA-D*). The strain *A. oryzae* NSAR1 (control) and the transformants were cultivated for 6 days in DPY media (28 °C, 110 rpm). The transformants were then extracted with ethyl acetate and submitted to LCMS for analysis. Out of the six transformants, only three transformants showed the production of a new compound that have the same mass and UV as that of ilicicolin-H **10** and 8-*epi*-ilicicolin-H **16** (Figure S8.40).

Isolation of this compound with molecular mass of 433 was done by cultivation of *A. oryzae* pTYGS·*argB·triliA·triliB·triliC·triliD* colony B in 1 L DPY media for 5 days then extraction and isolation of the compound using preparative HPLC. This afforded ~ 1 mg of compound **16** that was submitted for NMR analysis. 1 and 2D NMR analysis and comparison with published data (Table S7.5.) showed that the compound isolated was 8-*epi*-ilicicolin-H **16**. However, the NMR data showed that minor peaks correspond to ilicicolin-H (Table S7.6). HMBC correlations also confirmed the presence of ilicicolin-H **10** (Figures S8.38 and S8.39) in the mixture.

7. Tabulated NMR Data

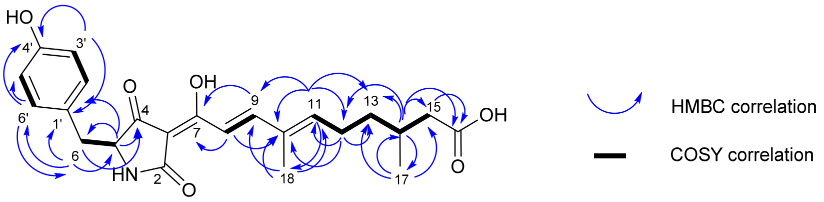

Table S7.1 Chemical shifts of compund **11** in Methanol-d4 and DMSO-d6.

	In methanol-d4				In DMSO-d6		
**Position**	**δC**	**δH (mult., int., J)**	**COSY**	**HMBC**	**δC**	**δH**	**HMBC (H to C)**
**2**	---------	---------	---------	---------	175.0	---------	---------
**3**	---------	---------	---------	---------	100.2	---------	---------
**4**	---------	---------	---------	6	195.0	---------	---------
**5**	64.0	4.08 (t, 1H, J = 5 Hz)	6, 1`	6, 1`	62.5	4.08 (1H, broad peak)	4, 1`(very weak)
**6**	37.7	(H_a) 2.87 (dd, 1H, J = 6.0, 14.0 Hz) (H_b) 3.00 (dd, 1H, J = 4.5, 14.0 Hz)	5	5, 1`, 2`, 6`	35.9	(H_a) 2.80 (dd, 1H, J = 5.3, 14.0 Hz) (H_b) 2.86 (dd, 1H, J = 4.7, 14.0 Hz)	4, 5, 1`, 2`, 6`
**7**	176.9	---------	---------	---------	173.2	---------	---------
**8**	116.7	7.10 (d, 1H, J = 15.6 Hz)	9	7, 9, 10, 11,	115.8	6.98 (overlap with 2`,6`)	---------
**9**	150.7	7.50 (d, 1H, J = 15.6 Hz)	8	8, 11, 12, 18	148.2	7.41 (d, 1H, J = 15.6 Hz)	7, 10, 11, 18
**10**	135.6	---------	---------	8, 9, 11, 12, 14	133.4	---------	---------
**11**	146.7	6.11 (t, 1H, J = 7.5 Hz)	12, 14	8, 9, 12, 13, 14	145.8	6.17 (1H, broad peak)	9, 12, 13, 18
**12**	27.7	2.33 (2H, m)	11	---------	26.3	2.26 (overlap with H-15b)	10, 11, 13
**13**	36.8	(H_a) 1.36 (1H, m) (H_b) 1.51 (1H, m)	12	11, 12, 14, 15, 17	35.1	(H_a) 1.28 (1H, m) (H_b) 1.43 (1H, m)	11, 12, 14, 15, 17
**14**	31.3	1.97 (1H, m)	15, 17	12, 13, 15, 16, 17	29.5	1.86 (1H, m)	12, 13, 15, 16, 17
**15**	42.4	(H_a) 2.14 (dd, 1H, J = 7.8, 15.0 Hz) (H_b) 2.32 (dd, 1H, J = 6.0, 15.0 Hz)	14	13, 14, 16, 17	41.1	(H_a) 2.04 (dd, 1H, J = 8.0, 15.0 Hz) (H_b) 2.25 (dd, 1H, J = 5.8, 15.0 Hz)	13, 14, 16, 17
**16**	176.2	---------	---------	14, 15	173.9	---------	---------
**17**	19.9	0.99 (d, 3H, J = 6.7 Hz)	14	13, 14, 15	19.4	0.91 (d, 3H, J = 6.7 Hz)	13, 14, 15
**18**	12.1	1.89 (s, 3H)	11	9, 10, 11	11.8	1.81 (s, 3H)	9, 10, 11
**1`**	127.8	---------	---------	5, 6, 3`, 5`	125.9	---------	---------
**2`, 6`**	131.8	6.99 (d, 2H, J = 8.5 Hz)	3`, 5`	6, 3`, 4`, 5`	130.6	6.95 (d, 2H, J = 8.5 Hz)	5, 6, 3`, 4`, 5`
**3`, 5`**	116.1	6.67 (d, 2H, J = 8.5 Hz)	2`, 6`	2`, 4`, 6`	114.9	6.62 (d, 2H, J = 8.5 Hz)	1`, 4`
**4`**	157.3	---------	---------	---------	155.9	---------	---------
**16-OH**	---------	---------	---------	---------	---------	12.00 (s, 1H)	---------
**4`-OH**	---------	---------	---------	---------	---------	9.19 (s, 1H)	2`, 3`, 4`, 5`, 6`

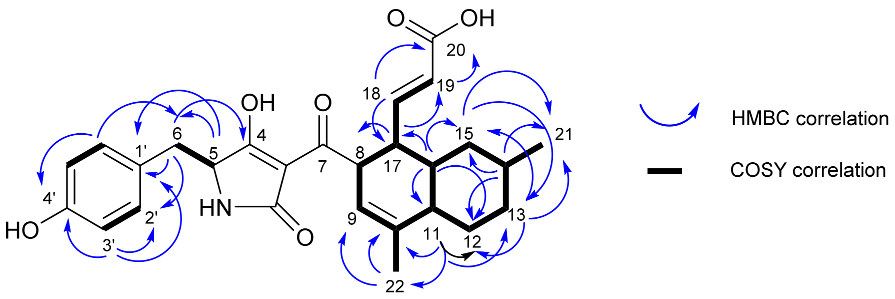

Table S7.2 Chemical shifts of compound **12** in Methanol-d4

POSITION	δC	δH (MULT., INT., *J*)	COSY	HMBC
**2**	173.8	-----------	-----------	-----------
**3**	101.5	-----------	-----------	-----------
**4**	196.8	-----------	-----------	-----------
**5**	64.0	4.00 (t, 1H, *J* = 5 Hz)	6	1`, 6
**6**	37.5	2.98 (dd, 1H, *J* = 4, 14.4 Hz)<br>2.86 (m, 1H)	5	4, 1`, 2`, 6`
**7**	190.0	-----------	-----------	-----------
**8**	45.5	4.29 (broad, 1H)	9, 17, 22	-----------
**9**	119.4	5.11 (broad, 1H)	8, 22	-----------
**10**	141.5	-----------	-----------	-----------
**11**	45.8	1.61 (m, 1H)	12, 22	10, 12, 13, 15, 22
**12**	30.6	Ha 1.04 (m, 1H)<br>Hb 2.08 (dd, 1H, *J* = 3, 12.3 Hz)	11, 13	11, 13
**13**	36.7	Ha 0.99 (m, 1H)<br>Hb 1.79 (d, 1H, *J* = 12.4 Hz)	12	12, 15, 21
**14**	34.0	1.44 (m, 1H)	21	12, 15, 21
**15**	41.1	Ha 0.54 (q, 1H, *J* = 12.2 Hz)<br>Hb 1.62 (m, 1H)	14, 16	13, 21
**16**	40.8	1.97 (m, 1H)	11, 15	11, 12, 15, 17
**17**	46.8	2.41 (m, 1H)	8, 18	8, 19
**18**	151.5	6.84 (dd, 1H, *J* = 10.4, 15.4 Hz)	17, 19	17, 20
**19**	124.3	5.82 (d, 1H, *J* = 15.5 Hz))	18	17, 20
**20**	169.5	-----------	-----------	-----------
**21**	23.0	0.89 (d, 3H, *J* = 6.5 Hz)	14	15
**22**	21.3	1.70 (s, 3H)	8, 9, 11	9, 10, 11, 13
**1`**	127.6	-----------	-----------	-----------
**2`, 6`**	131.8	6.99 (d, 2H, *J* = 8.4 Hz)	3`, 5`	6, 4`
**3`, 5`**	116.1	6.67 (d, 2H, *J* = 8.4 Hz)	2`, 6`	1`, 2`, 6`, 4`
**4`**	157.4	-----------	-----------	-----------

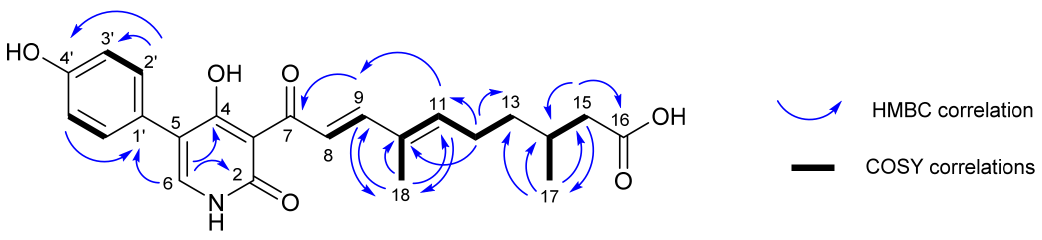

Table S7.3 Chemical shifts of compound **14** in deuterated Methanol-d4.

**Pos.**	δ_C	δ_H **(MULT., INT., *J*)**	COSY	HMBC
**2**	164.5	------------	---------	---------
**3**	------	------------	---------	---------
**4**	178.1	------------	---------	---------
**5**	-------	------------	---------	---------
**6**	140.6	7.47 (s, 1H)	---------	2, 4, 1`
**7**	195.9	---------------	---------	---------
**8**	124.5	7.97 (d, 1H, *J* = 15.5 Hz)	9	--------
**9**	151.1	7.59 (d, 1H, *J* = 15.5 Hz)	8	7, 18
**10**	135.8			
**11**	145.6	6.10 (t, 1H, *J* = 7.5 Hz)	12, 18	9, 18
**12**	27.6	2.33 (m, 2H)	11	10, 11, 13
**13**	37.0	Ha 1.36 (m, 1H)<br>Hb 1.53 (m, 1H)	12	---------
**14**	31.5	1.97 (m, 1H)	15, 17	---------
**15**	43.4	Ha 2.11 (q, 1H, *J* = 14.0, 8.0 Hz)<br>Hb 2.30 (m, 1H)	14	14, 16, 17
**16**	178.2	--------------	---------	---------
**17**	20.1	1.00 (d, 3H, *J* = 7.0 Hz)	14	13, 14, 15
**18**	12.5	1.92 (s, 3H)	11	9, 10, 11
**1´**	125.4	----------------	---------	---------
**2´, 6´**	131.4	7.29 (d, 1H, *J* = 8.5 Hz)	3', 5'	3', 4', 5'
**3´, 5´**	116.1	6.82 (d, 1H, *J* = 8.5 Hz)	2', 6'	1'
**4´**	158.3	-----------------	---------	---------

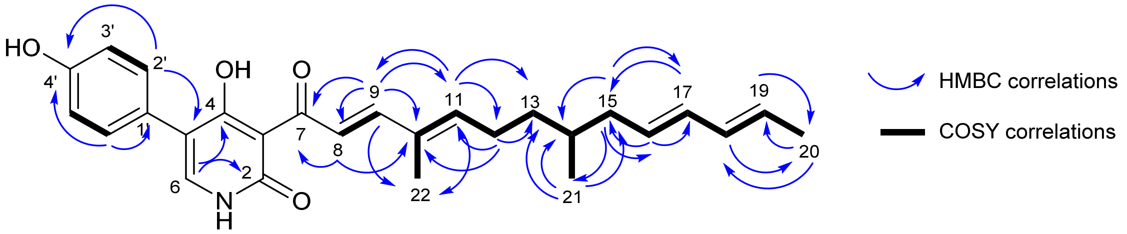

Table S7.4 Chemical shifts of compound **15** (Comparison with reported NMR data[23])

Pos.	Reported NMR (in $CDCl_3$: acetone-d_6 = 3:1)[23]		Found NMR (in Acetone-d_6)	
	δ_C	δ_H **(MULT., INT., *J*)**	δ_C	δ_H **(MULT., INT., *J*)**
**2**	162.9	-------	162.7	---------
**3**	106.7	---------	107.2	---------
**4**	178.0	--------	179.0	---------
**5**	114.8	-----------	114.6	---------
**6**	139.1	7.44 (s, 1H)	140.8	7.61 (obscured, 1H)
**7**	194.6	7	195.2	
**8**	123.3	8.01 (d, 1H, *J* = 15.5 Hz)	124.2	8.15 (d, 1H, *J* = 15.5 Hz)
**9**	150.1	7.53 (d, 1H, *J* = 15.5 Hz)	150.4	7.59 (d, 1H, *J*= 15.5 Hz))
**10**	134.5	---------	135.1	---------
**11**	144.8	6.01 (t, 1H, *J* = 7.5 Hz)	145.5	6.15 (t, 1H, *J* = 7.5 Hz)
**12**	26.8	2.22 (m, 2H,)	27.4	2.32 (m)
**13**	35.5	Ha 1.22 (m, 1H)<br>Hb 1.42 (m, 1H)	36.4	1.30<br>1.51
**14**	33.1	1.50 (m, 1H)	33.8	1.58 (m, 1H)
**15**	40.0	Ha 1.88 (q, 1H, *J* = 13.0, 7.0 Hz)<br>Hb 2.02 (q, 1H, *J* = 13.0, 6.0 Hz)	40.5	1.94 (m)<br>2.11 (m)
**16**	130.0	5.45 (m, 1H)	130.8	5.55 (m, 1H)
**17**	131.4	5.88 (m, 1H)	132.8	6.00 (m, 1H)
**18**	131.6	5.94 (m, 1H)	132.8	6.04 (m, 1H)
**19**	126.7	5.50 (m, 1H)	127.3	5.56 (m, 1H)
**20**	17.9	1.65 (d, 3H, *J* = 6.5 Hz)	18.1	1.69 (d, , 3H, *J* = 6.5 Hz)
**21**	19.4	0.85 (d, 3H, *J* = 7.0 Hz)	19.7	0.924 (d, 3H, *J* = 7.0 Hz)
**22**	12.4	1.84 (s, 3H)	12.5	1.90 (s, 3H)
**1'**	124.4	---------	125.4	---------
**2'/6'**	130.3	7.26 (d, 1H, *J* = 8.5 Hz)	131.1	7.34 (d, 1H, *J* = 8.5 Hz)
**3'/5'**	115.4	6.83 (d, 1H, *J* = 8.5 Hz)	115.9	6.87 (d, 1H, *J* = 8.5 Hz)
**4'**	156.9	---------	157.8	---------

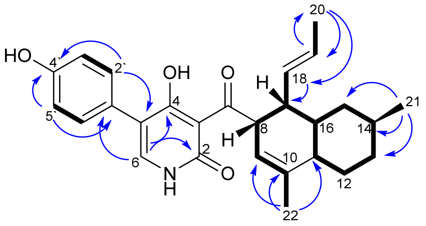

Table S7.5 Chemical shifts of compound **16** (Comparison with reported NMR data[23])

Pos.	Reported NMR for 8-epi-ilicicolin-H (in acetonitrile-d_3)[23]		Found NMR for 8-epi-ilicicolin-H (in acetonitrile-d_3)	
	δ_C	δ_H (MULT., INT., *J*)	δ_C	δ_H (MULT., INT., *J*)
2	162.9	----------------	162.4	----------------
3	108.0	----------------	---------------	----------------
4	177.6	----------------	177.7	----------------
5	114.8	----------------	114.3	----------------
6	140.8	7.42 (s, 1H)	140.7	7.40 (s, 1H)
7	209.8	----------------	209.5	----------------
8	50.8	5.07 (m, 1H)	50.7	5.07 (m, 1H)
9	120.1	5.47 (m, 1H)	120.1	5.45 (m, 1H)
10	141.2	----------------	141.2	----------------
11	46.0	1.54 (m, 1H)	45.9	1.55 (m, 1H)
12	30.4	Ha 1.00 (m, 1H)<br>Hb 2.03 (m, 1H)	30.4	Ha 0.99 (m, 1H)<br>Hb 2.07 (m, 1H)
13	36.3	Ha 0.93 (m, 1H)<br>Hb 1.77 (m, 1H)	36.3	Ha 0.95 (m, 1H)<br>Hb 1.78 (m, 1H)
14	33.7	1.41 (m, 1H)	33.7	1.42 (m, 1H)
15	40.7	Ha 0.44 (q, 1H, *J* = 24.5, 12.0 Hz)<br>Hb 1.74 (m, 1H)	40.7	Ha 0.46 (q, 1H, *J* = 24.5, 12.0 Hz)<br>Hb 1.77 (m, 1H)
16	40.6	1.93 (m, 1H)	40.7	1.94 (obscured by solvent peak, 1H)
17	47.4	2.17 (m, 1H)	47.5	2.18 (Obscured by H_2O peak, 1H)
18	133.4	5.45 (m, 1H)	133.5	5.46 (m, 1H)
19	127.3	5.44 (m, 1H)	127.2	5.46 (m, 1H)
20	18.1	1.54 (d, 3H)	18.0	1.56 (d, 3H, *J* = 4.5 Hz)
21	23.0	0.88 (d, 3H, *J* = 6.5 Hz)	23.1	0.89 (d, 3H, *J* = 6.5 Hz)
22	21.5	1.66 (s, 3H)	21.6	1.68 (m, 3H)
1`	125.4	----------------	125.5	----------------
2`, 6`	131.4	7.26 (d, 1H, *J* = 9.0 Hz)	131.4	7.27 (d, 1H, *J* = 8.7 Hz)
3`, 5`	116.0	6.83 (d, 1H, *J* = 9.0 Hz)	116.1	6.84 (d, 1H, *J* = 8.7 Hz)
4`	157.5	10.09 (s, OH)	157.5	----------------

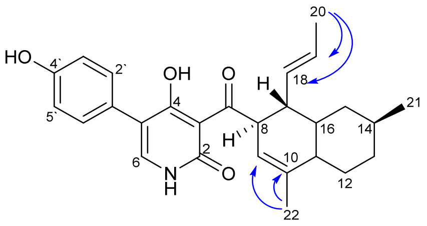

Table S7.6 Chemical shifts of compound **10** (Comparison with reported NMR data[23])

Pos.	Reported NMR for ilicicolin-H (in acetonitrile-d_3)[23]		Found NMR for ilicicolin-H (in acetonitrile-d_3)	
	δ_C	δ_H **(MULT., INT., *J*)**	δ_C	δ_H **(MULT., INT., *J*)**
**2**	162.8	----------------	162.4	----------------
**3**	107.8	----------------	----------------	----------------
**4**	177.6	----------------	177.7	----------------
**5**	114.6	----------------	114.3	----------------
**6**	140.9	7.43 (s, 1H)	140.7	7.41 (s, 1H)
**7**	210.7	----------------	----------------	----------------
**8**	53.8	4.98 (m, 1H)	53.6	4.99 (m, 1H)
**9**	120.4	5.21 (m, 1H)	120.4	5.21 (m, 1H)
**10**	139.1	----------------	139.1	----------------
**11**	45.1	1.67 (m, 1H)	45.2	1.69 (m, 1H)
**12**	30.6	Ha 0.97 (m, 1H)<br>Hb 2.03 (m, 1H)	30.4	Ha 1.02 (m, 1H)<br>Hb 2.05 (m, 1H)
**13**	36.2	Ha 0.97 (m, 1H)<br>Hb 1.76 (m, 1H)	36.3	Ha 0.99 (m, 1H)<br>Hb 1.76 (m, 1H)
**14**	33.4	1.38 (m, 1H)	33.5	1.40 (m, 1H)
**15**	40.7	Ha 0.57 (q, 1H)<br>Hb 1.76 (m, 1H)	40.2	Ha 0.59 (q, 1H)<br>Hb 1.77 (m, 1H)
**16**	44.2	1.22 (m, 1H)	44.3	1.20 (m, 1H)
**17**	45.8	2.48 (m, 1H)	45.9	2.48 (m, 1H)
**18**	134.2	5.21 (m, 1H)	134.2	5.24 (m, 1H)
**19**	126.8	5.32 (m, 1H)	126.6	5.32 (m, 1H)
**20**	18.0	1.52 (dd, 3H, *J* = 6.5, 1.3 Hz)	18.0	1.56 (d, 3H, *J* = 4.5 Hz)
**21**	23.0	0.88 (d, 3H, *J* = 6.5 Hz)	23.0	0.89 (d, 3H, *J* = 6.5 Hz)
**22**	21.0	1.62 (s, 3H)	21.0	1.65 (m, 3H)
**1`**	125.3	----------------	125.5	----------------
**2`, 6`**	131.4	7.26 (d, 1H, *J* = 8.6 Hz)	131.4	7.27 (d, 1H, *J* = 9.0 Hz)
**3`, 5`**	116.0	6.83 (d, 1H, *J* = 8.6 Hz)	116.1	6.84 (d, 1H, *J* = 9.0 Hz)
**4`**	157.5	----------------	157.5	----------------

8. NMR Spectra

Compound 11

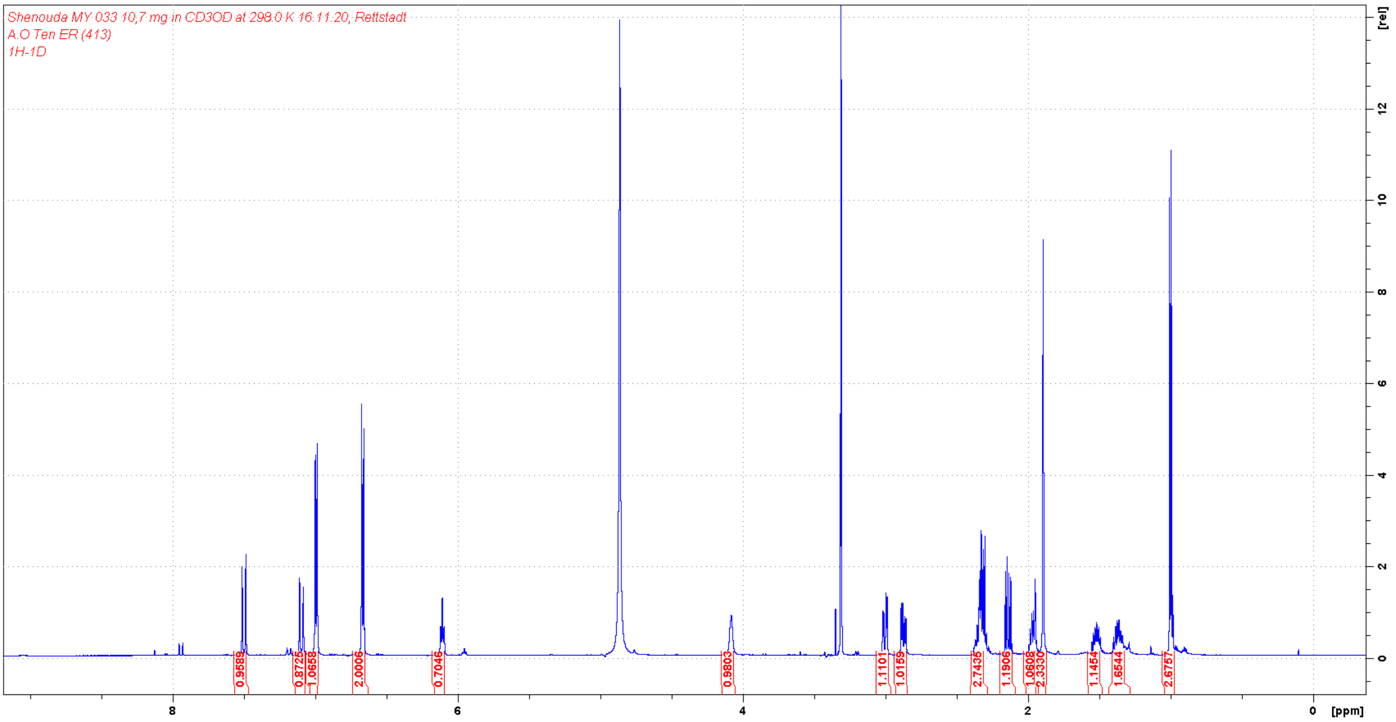

Figure S8.1 ^{1}H-NMR spectrum of compound **11** (500 MHz, CD_3OD).

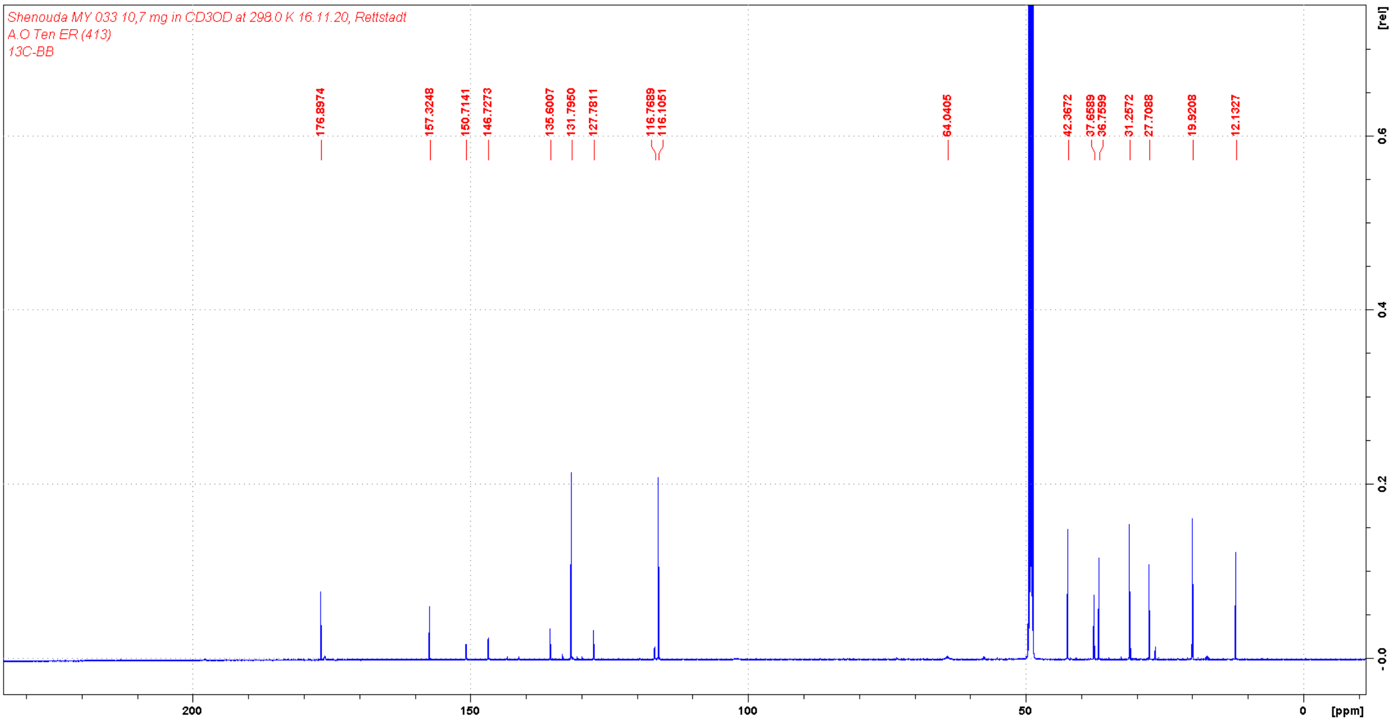

Figure S8.2 ^{13}C-NMR spectrum of compound **11** (125 MHz, CD_3OD).

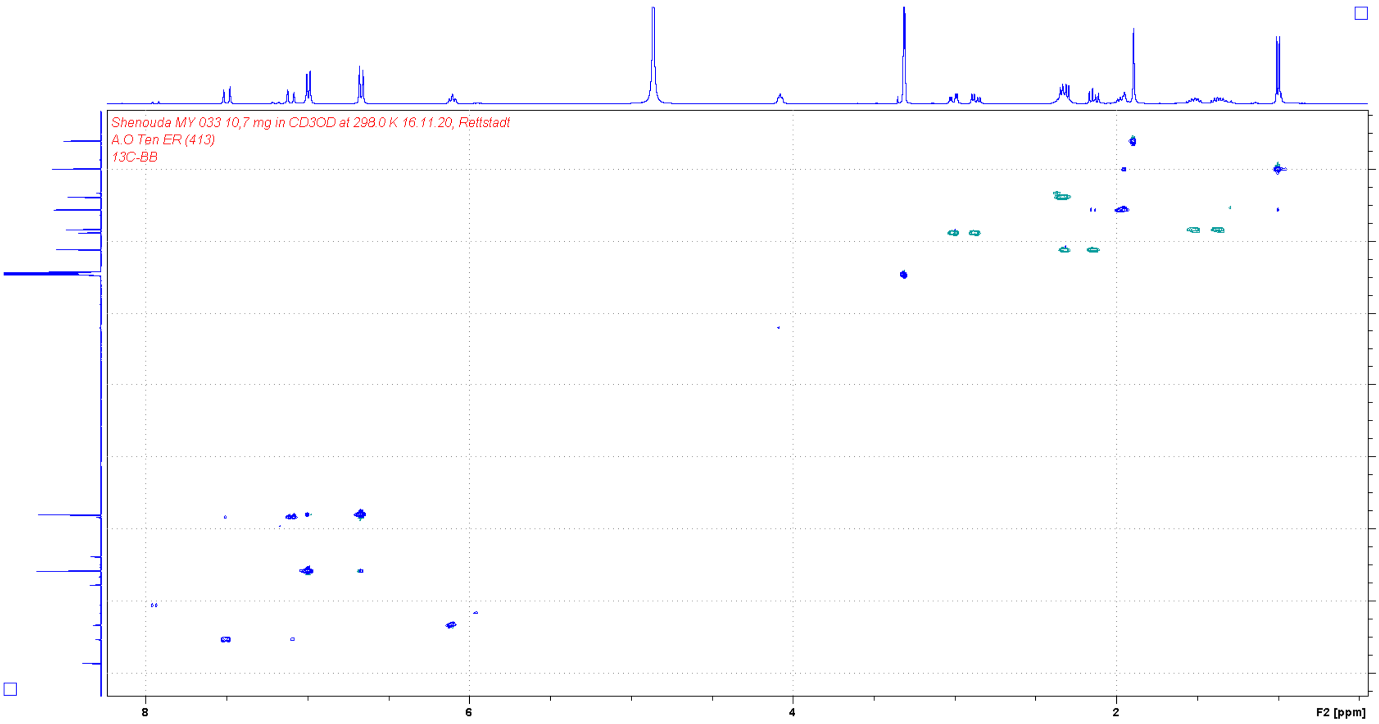

Figure S8.3 HSQC spectrum of compound **11** in CD_3OD.

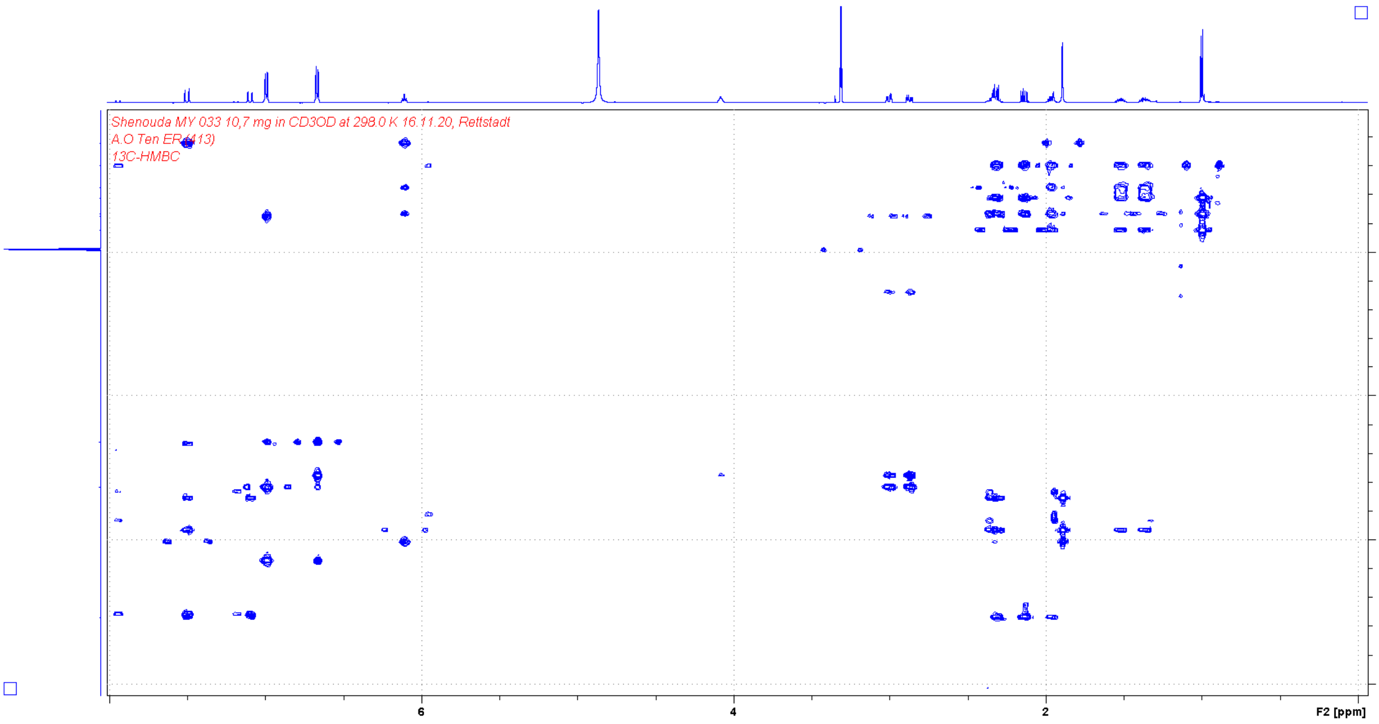

Figure S8.4 HMBC spectrum of compound **11** in CD_3OD.

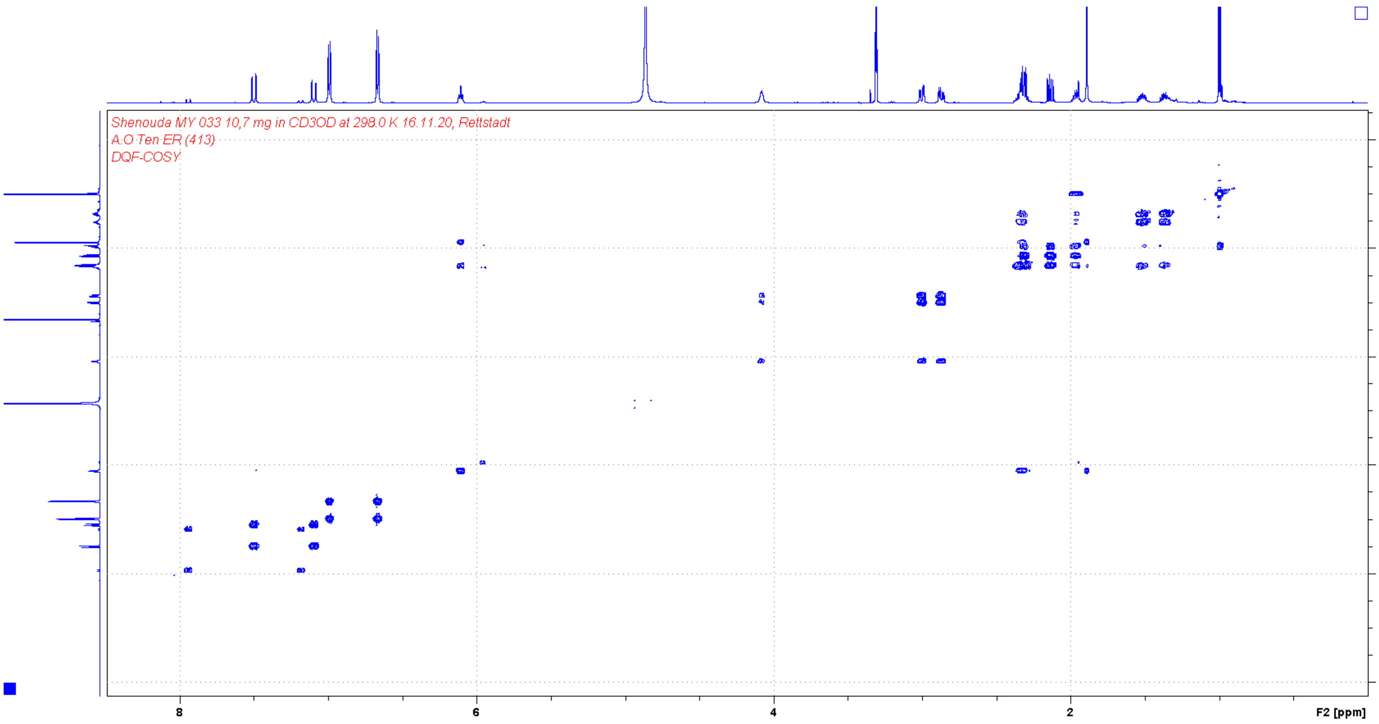

Figure S8.5 COSY spectrum of compound **11** in CD_3OD.

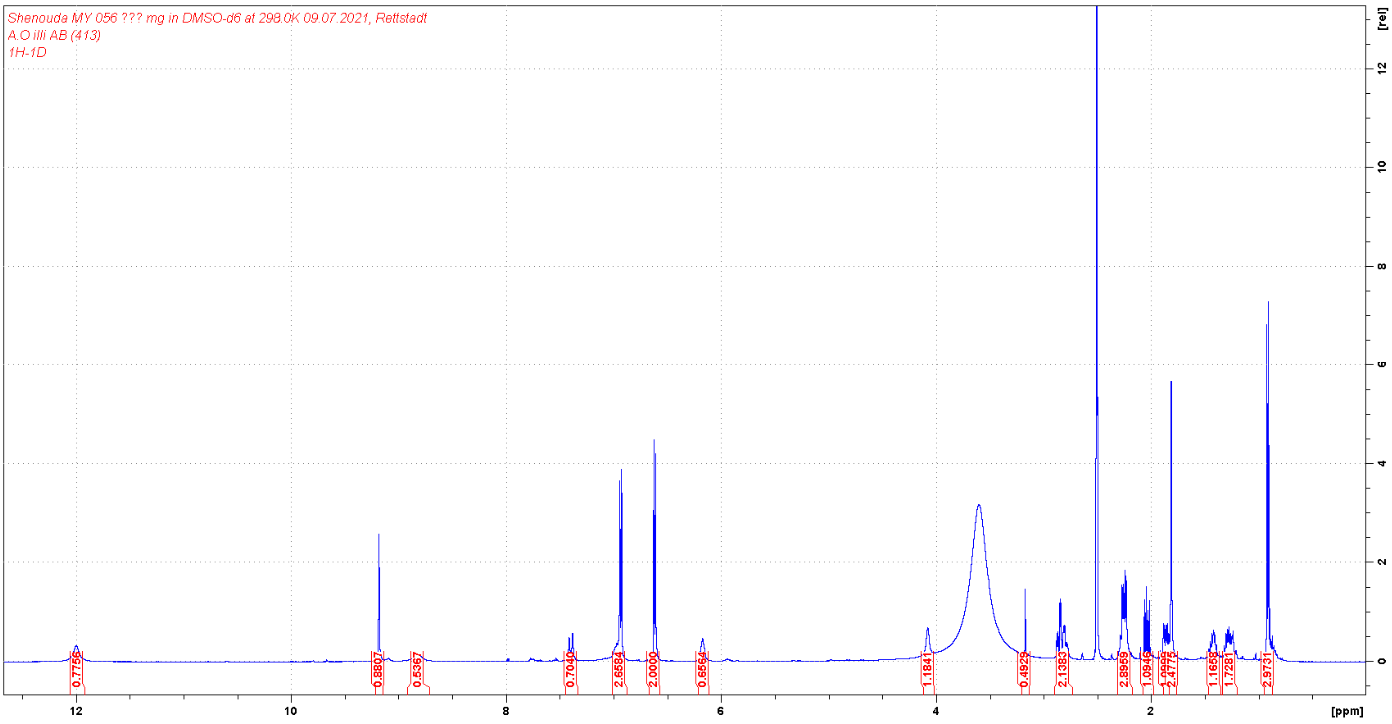

Figure S8.6 ^{1}H-NMR spectrum of compound **11** (500 MHz, DMSO-d6).

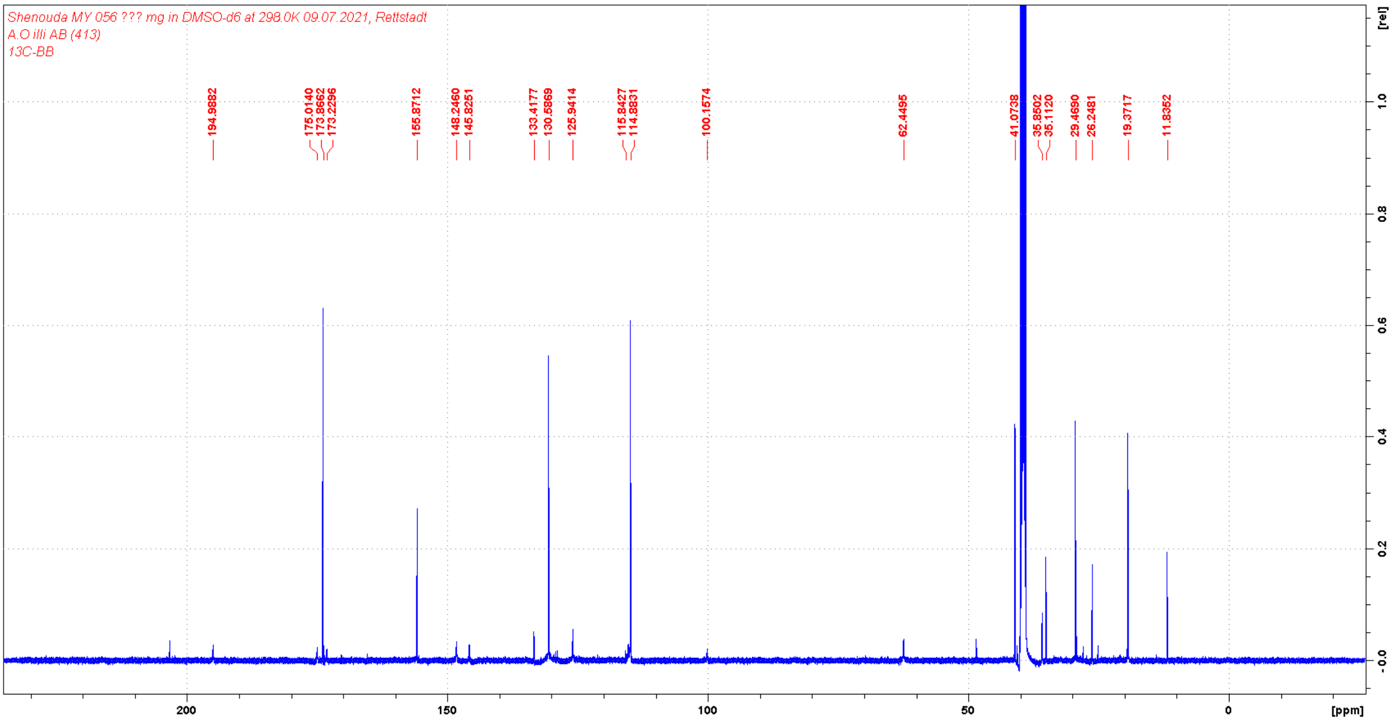

Figure S8.7 ^{13}C-NMR spectrum of compound **11** (125 MHz, DMSO-d$_6$).

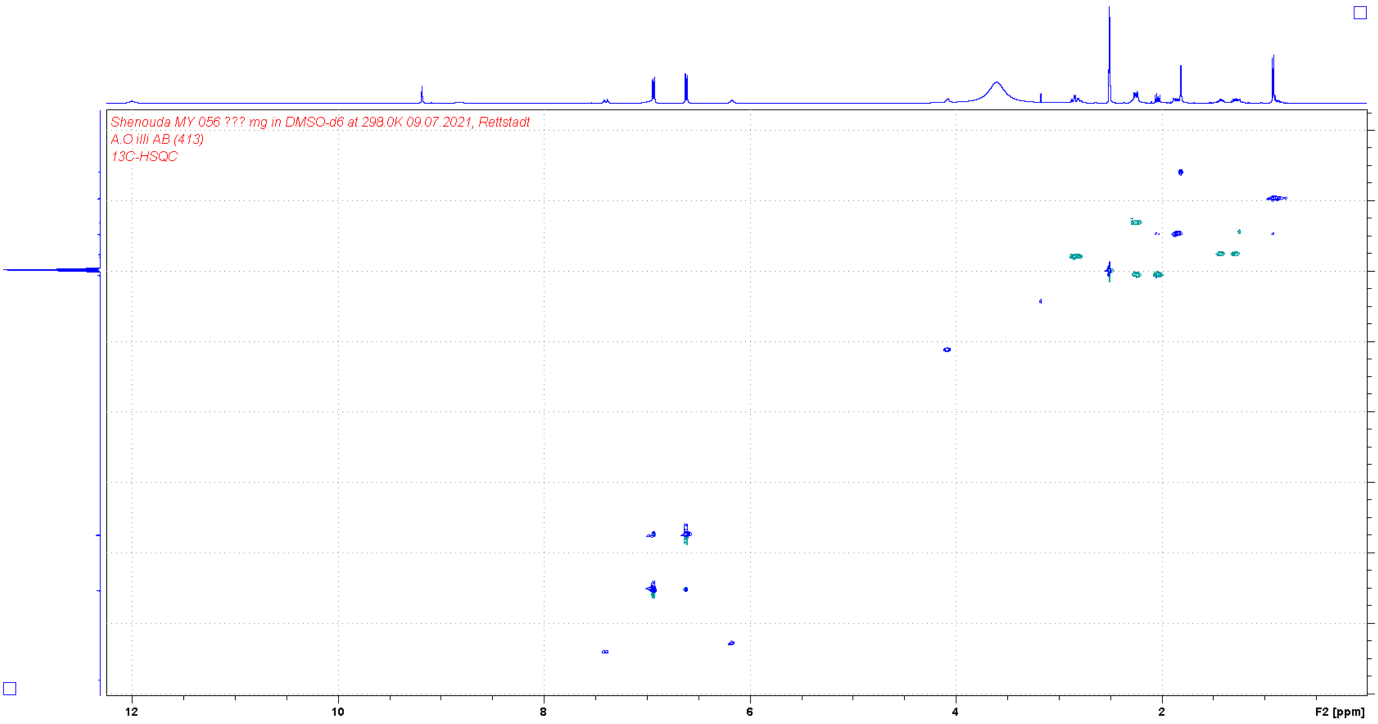

Figure S8.8 HSQC spectrum of compound **11** (DMSO-d$_6$).

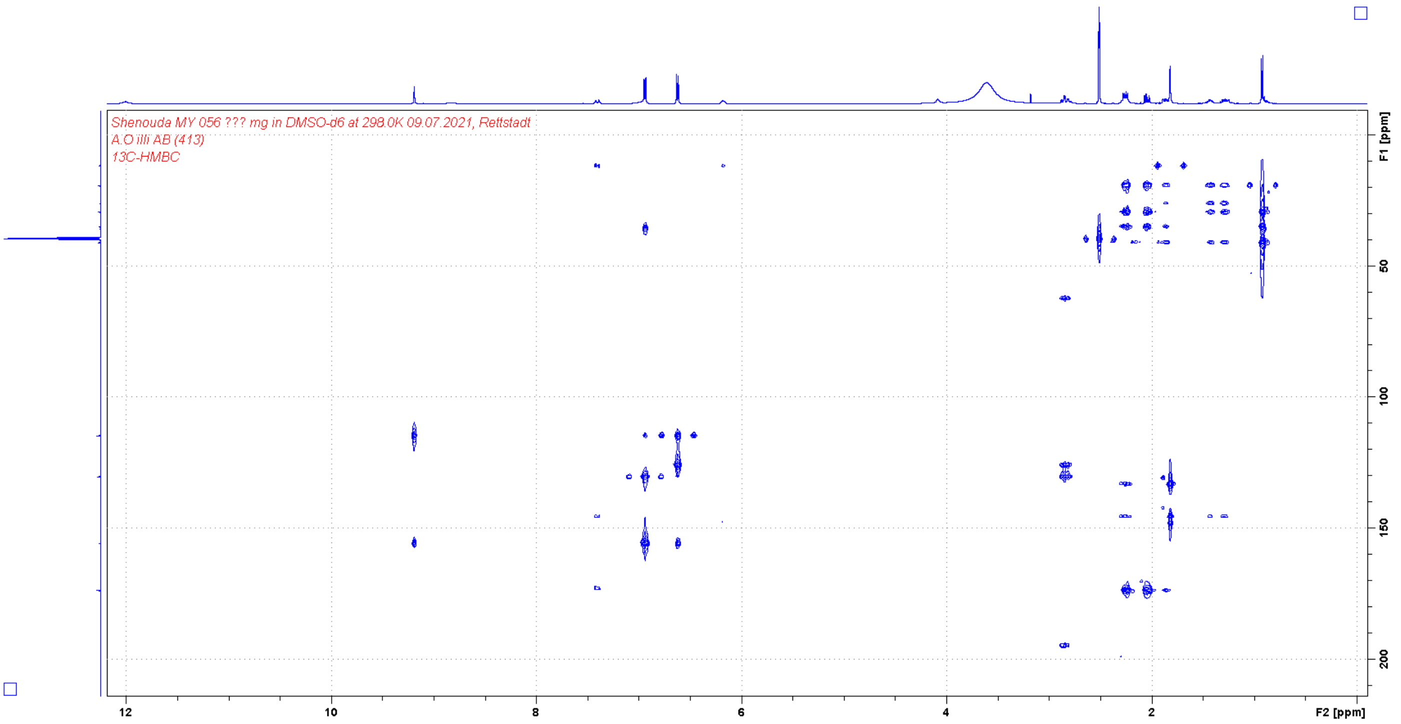

Figure S8.9 HMBC spectrum of compound **11** (DMSO-d_6).

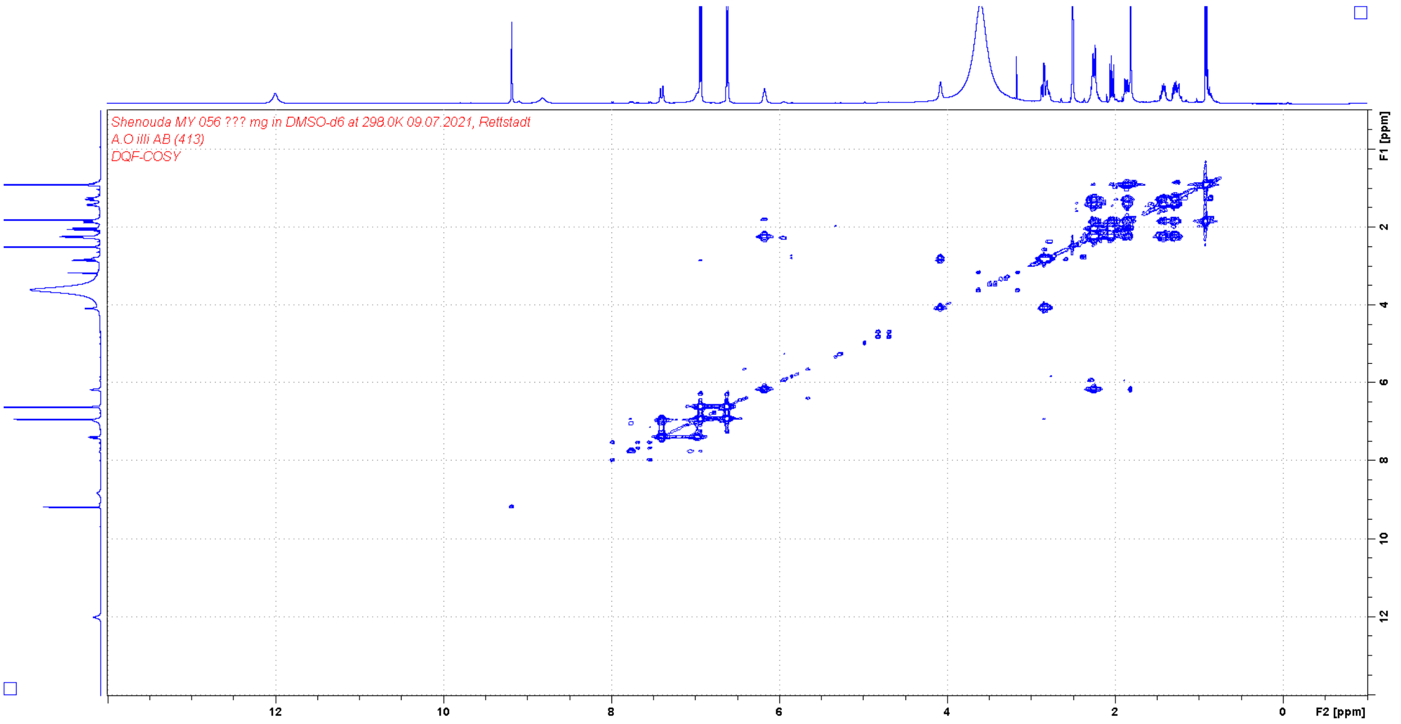

Figure S8.10 COSY spectrum of compound **11** (DMSO-d_6).

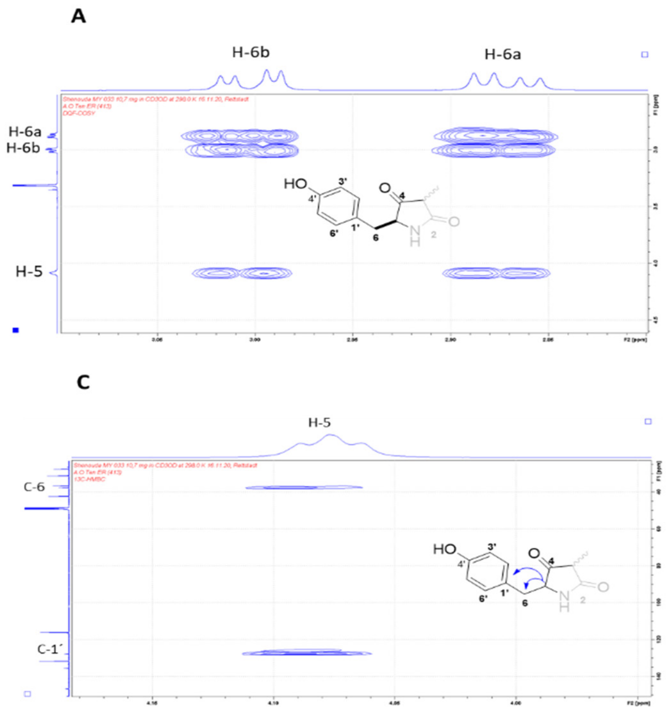

Figure S8.11 NMR spectra of compound **11**: **A**, COSY correlation of H-6; **B**, HMBC correlation of H-6; **C**, HMBC correlation of H-5; **D**, HMBC correlation of H-3´,5´and 2´,6´.

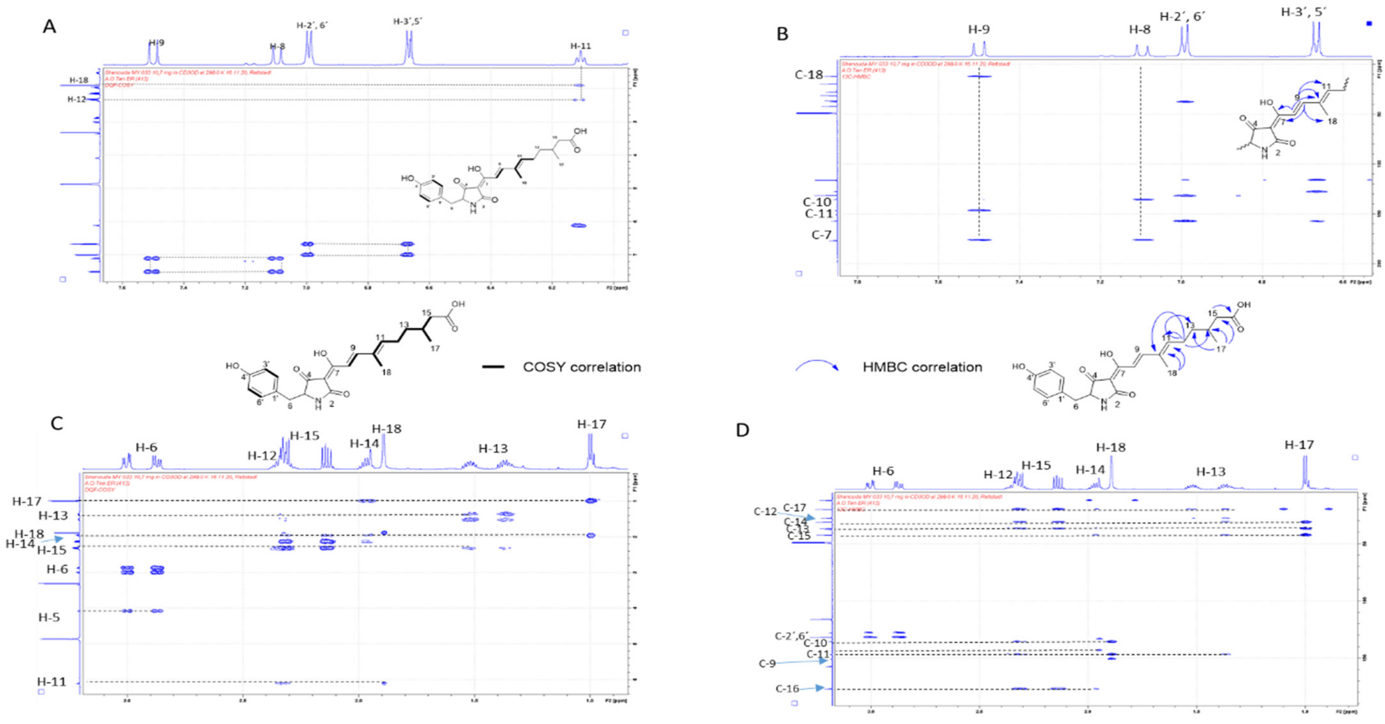

Figure S8.12 NMR spectra of compound **11**: **A**, COSY correlations of H-8, 9, 11, 2`, 6`and 3`,5`; **B**, HMBC correlation of H-8 and H-9; **C**, COSY correlation of H-6, 12, 13, 14, 15, 17 and 18; **D**, HMBC correlation of H-6, 12, 13, 14, 15, 17 and 18.

Compound 12

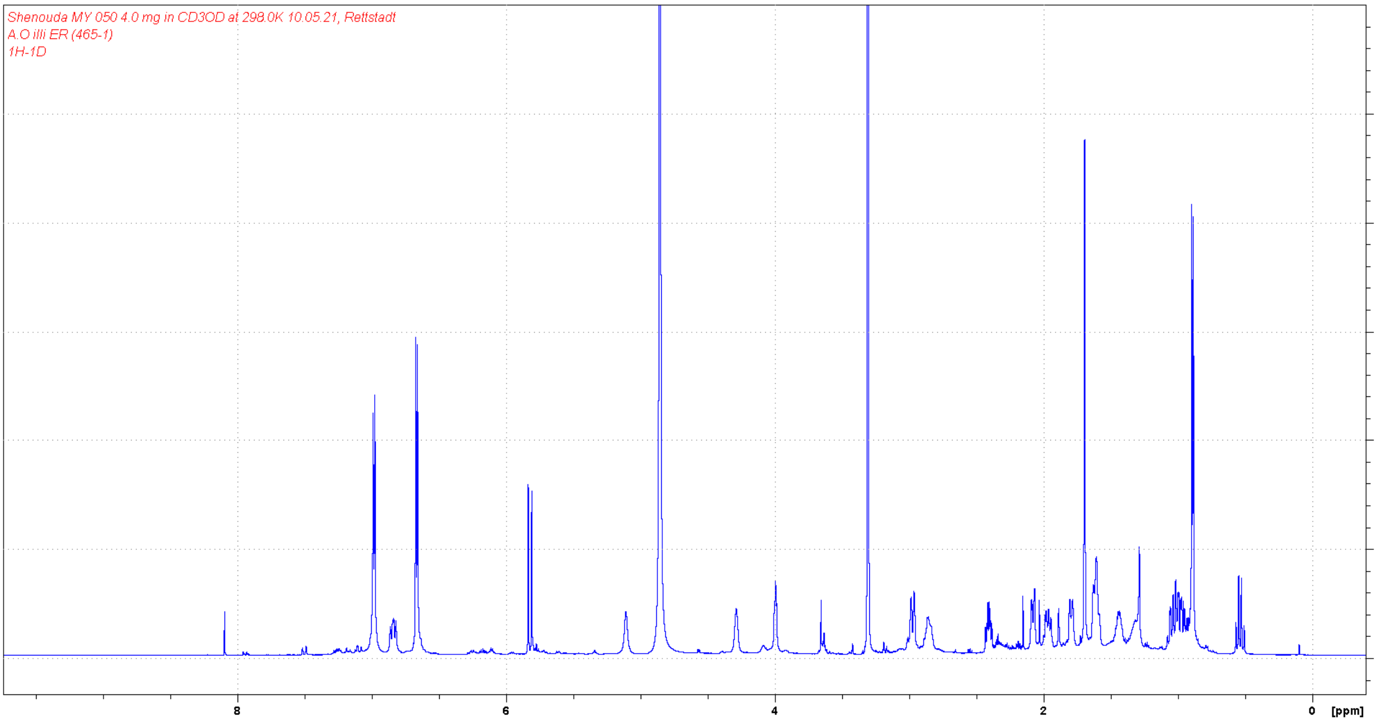

Figure S8.13 ^{1}H-NMR spectrum of compound **12** (500 MHz, CD_3OD).

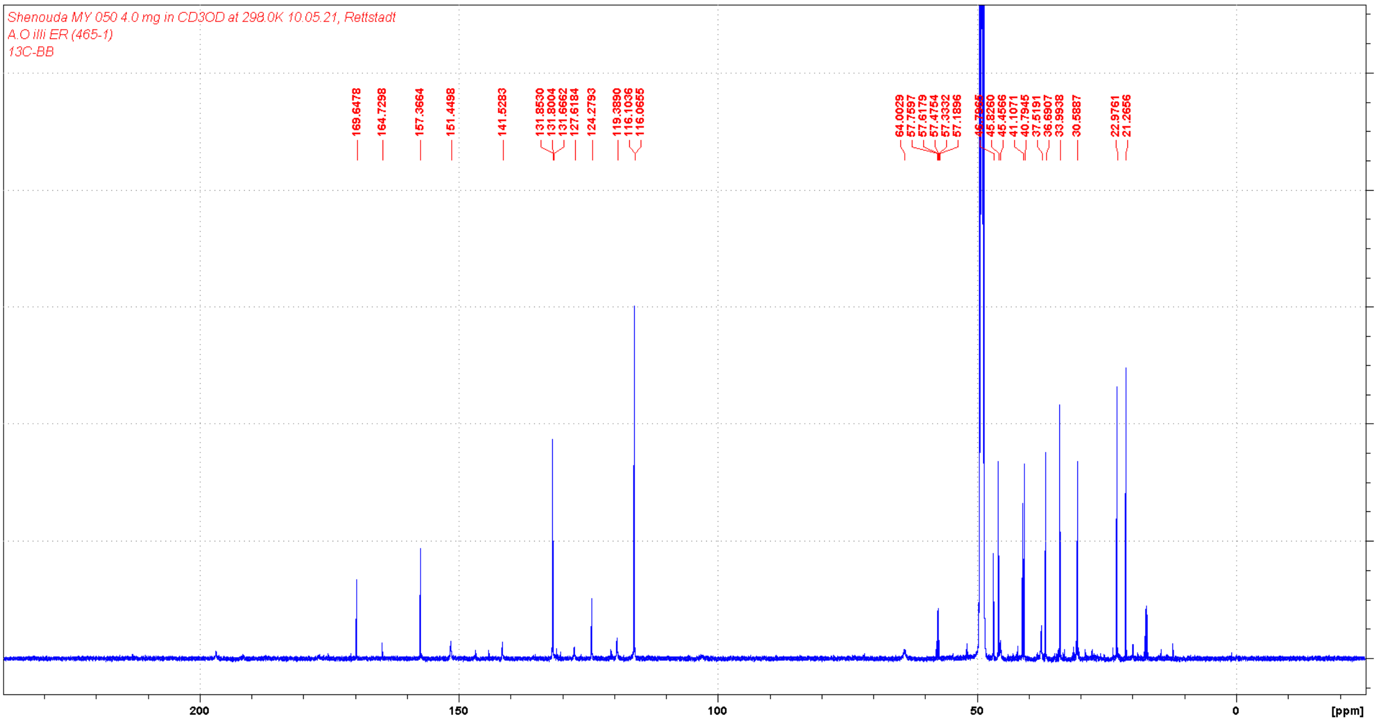

Figure S8.14 ^{13}C-NMR spectrum of compound **12** (125 MHz, CD_3OD).

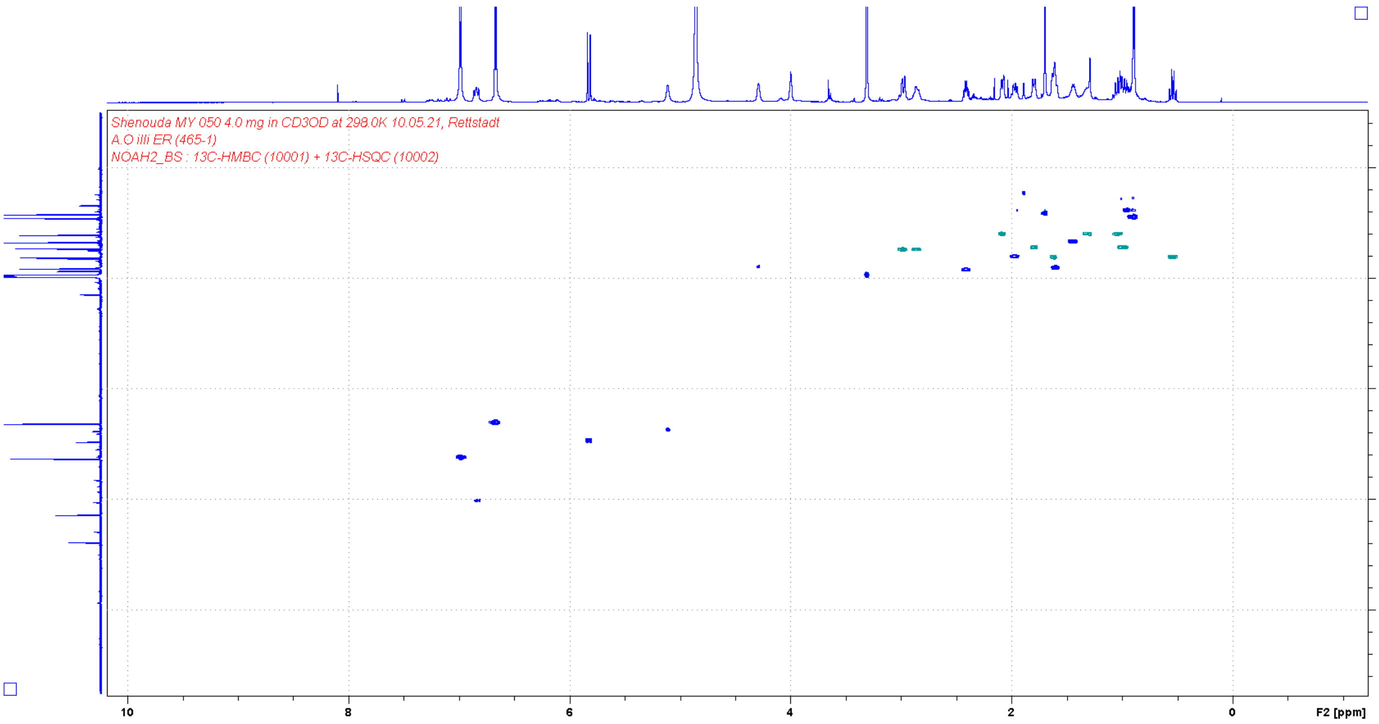

Figure S8.15 HSQC spectrum of compound **12**.

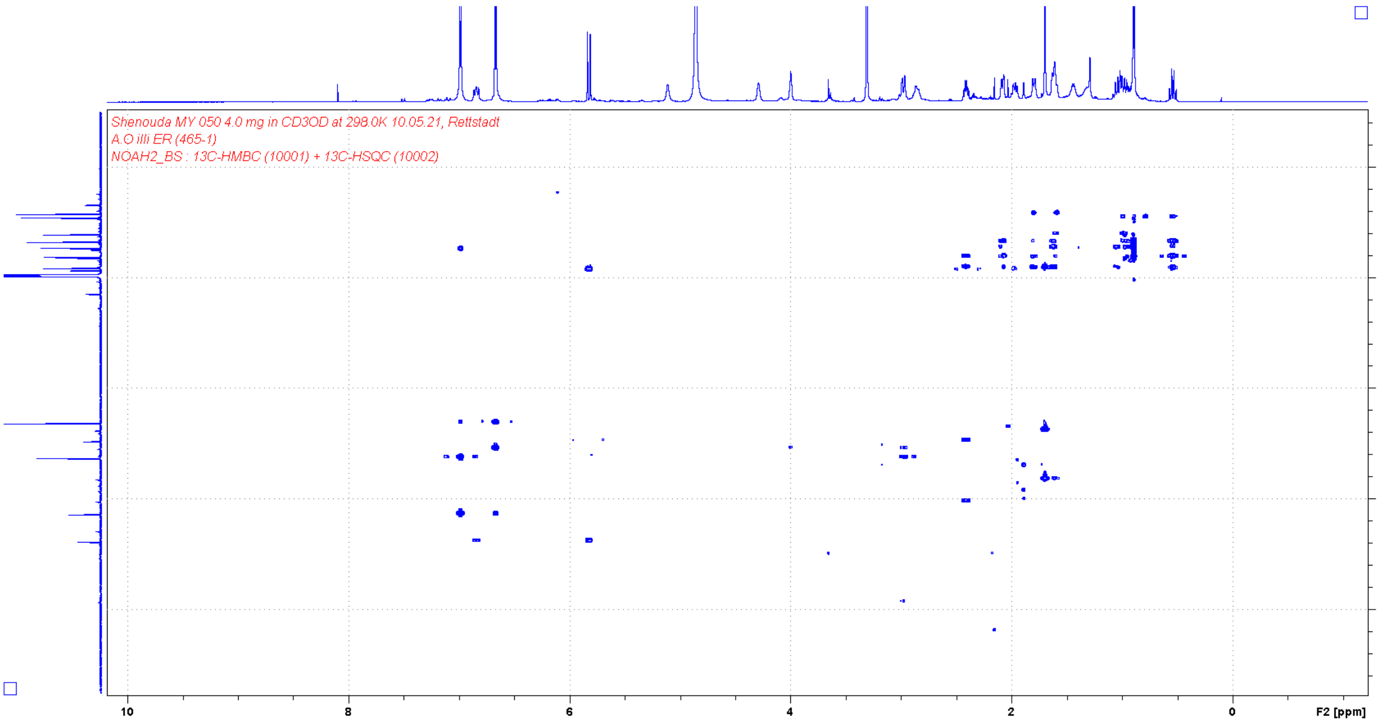

Figure S8.16 HMBC spectrum of compound **12**.

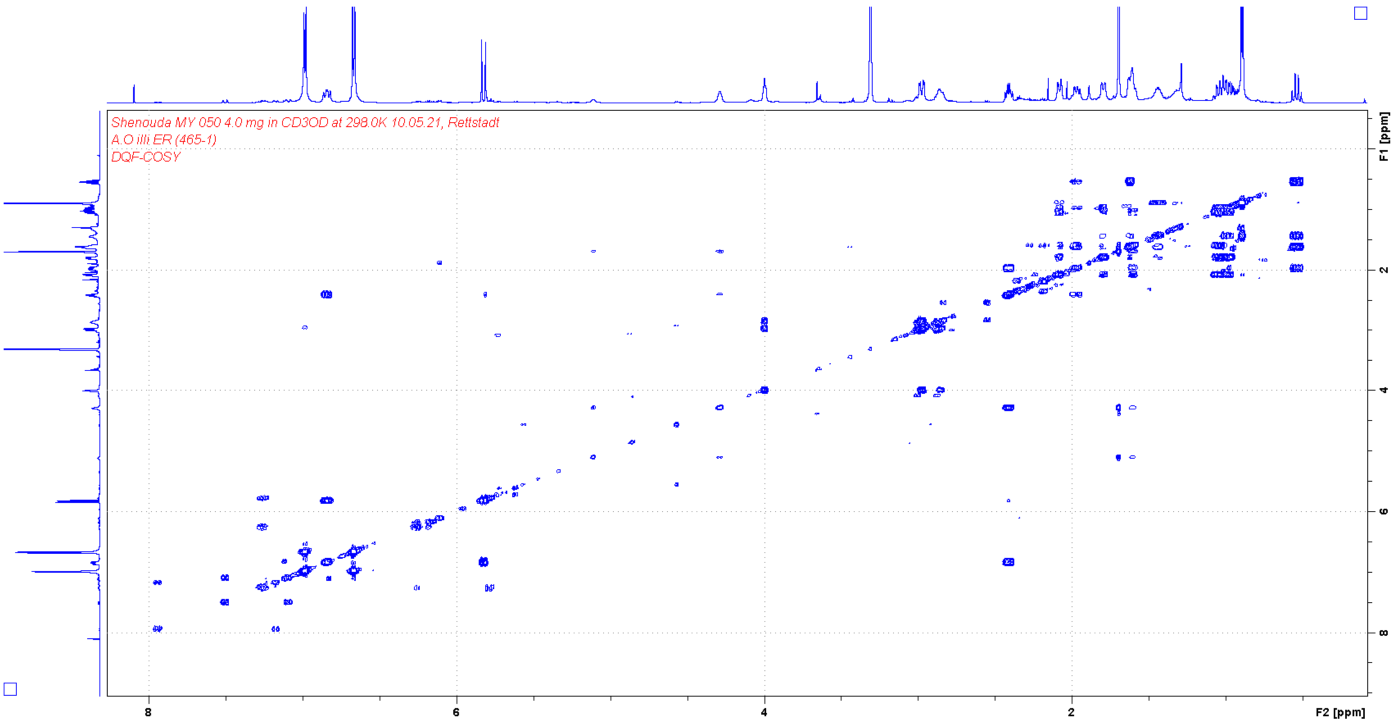

Figure S8.17 COSY spectrum of compound **12**.

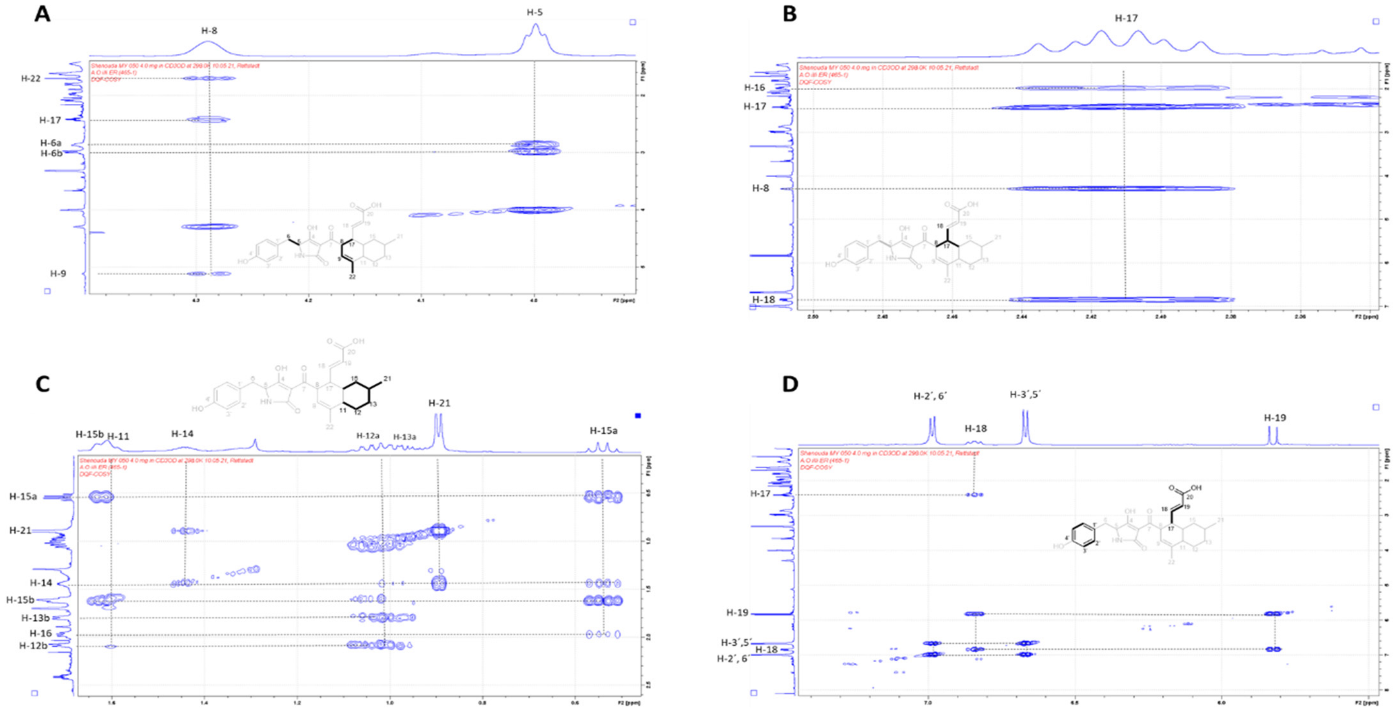

Figure S8.18 COSY correlations of compound **12**: **A**, COSY correlation of H-5 and H-8; **B**, COSY correlations of H-17; **C**, COSY correlation of H-11, 12, 14, 15 and 21; **D**, COSY correlation of H-18, 19 and H2`, 3`, 5`, 6`

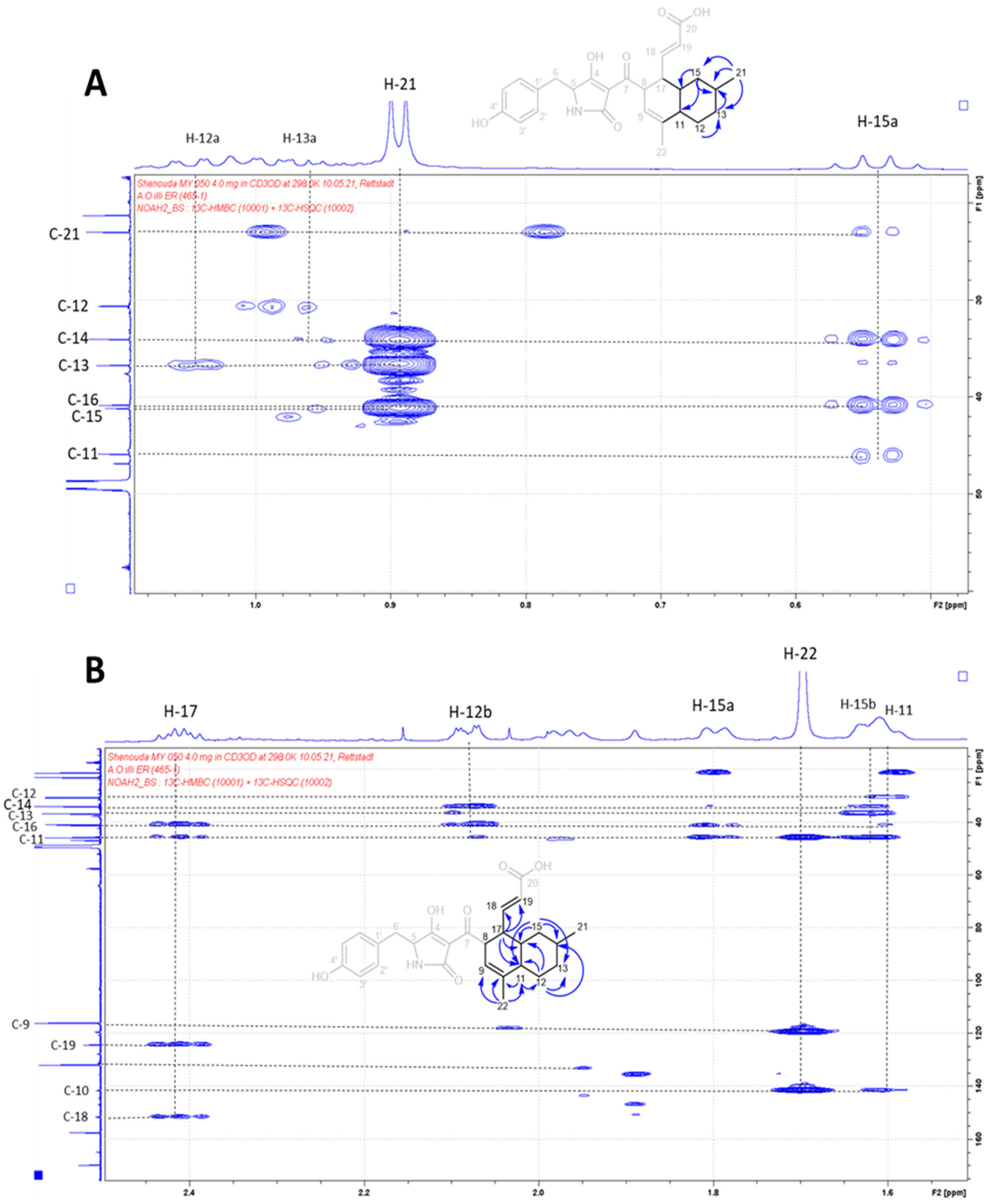

Figure S8.19 HMBC correlations of the decalin structure of compound **12**.

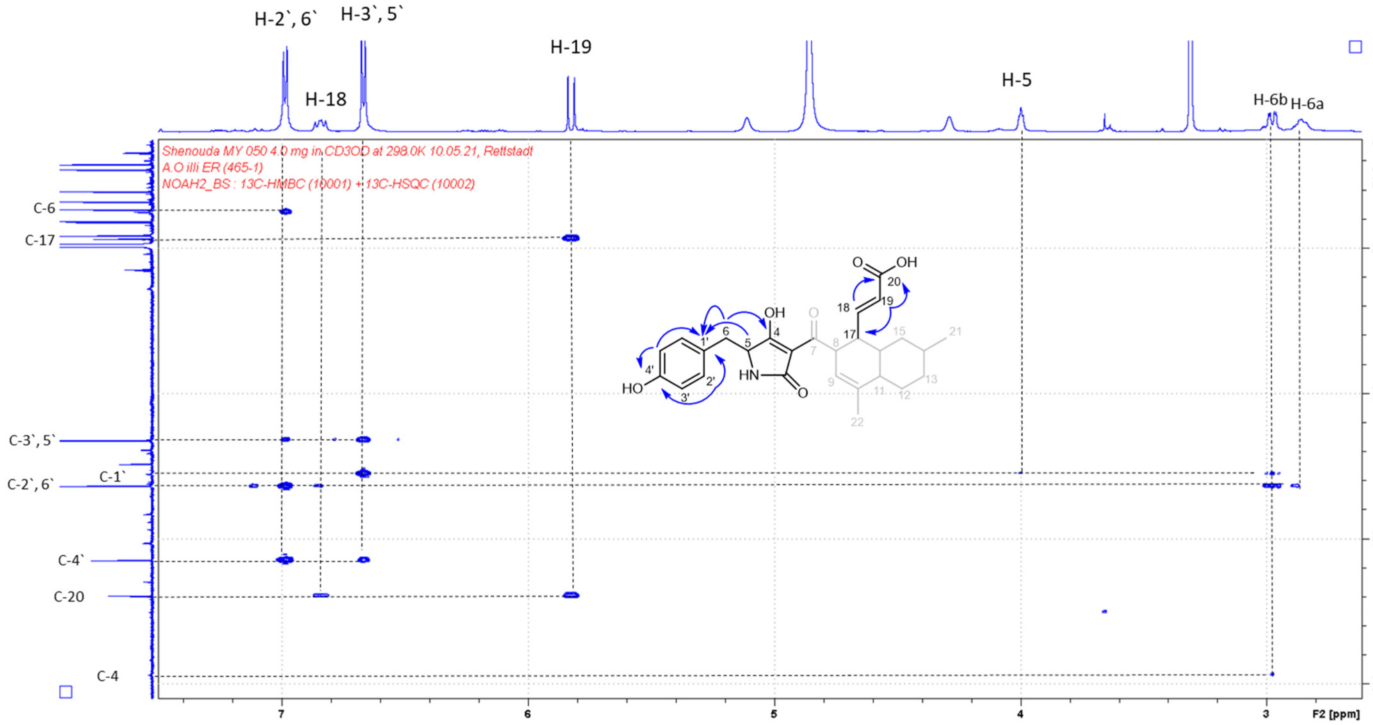

Figure S8.20 HMBC correlations of compound **12**.

Compound 14

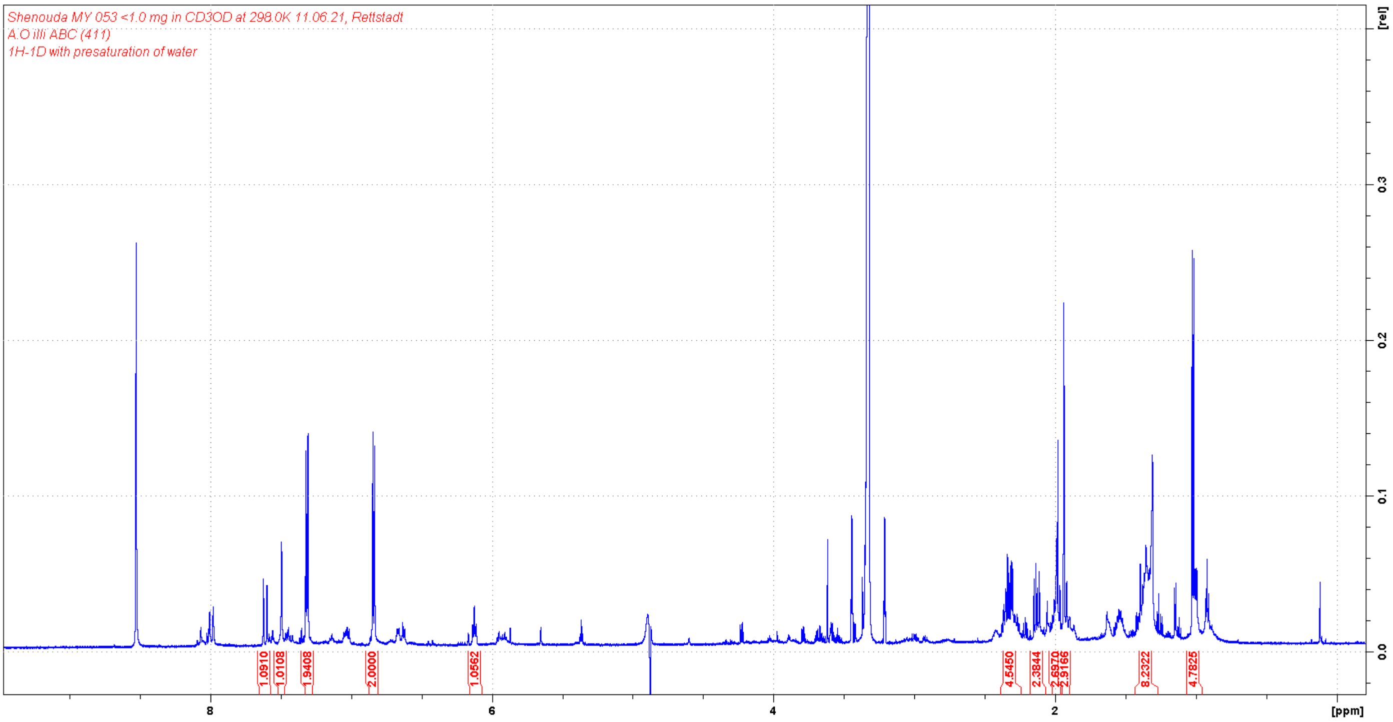

Figure S8.21 ^{1}H-NMR spectrum of compound **14** (500 MHz, CD_3OD).

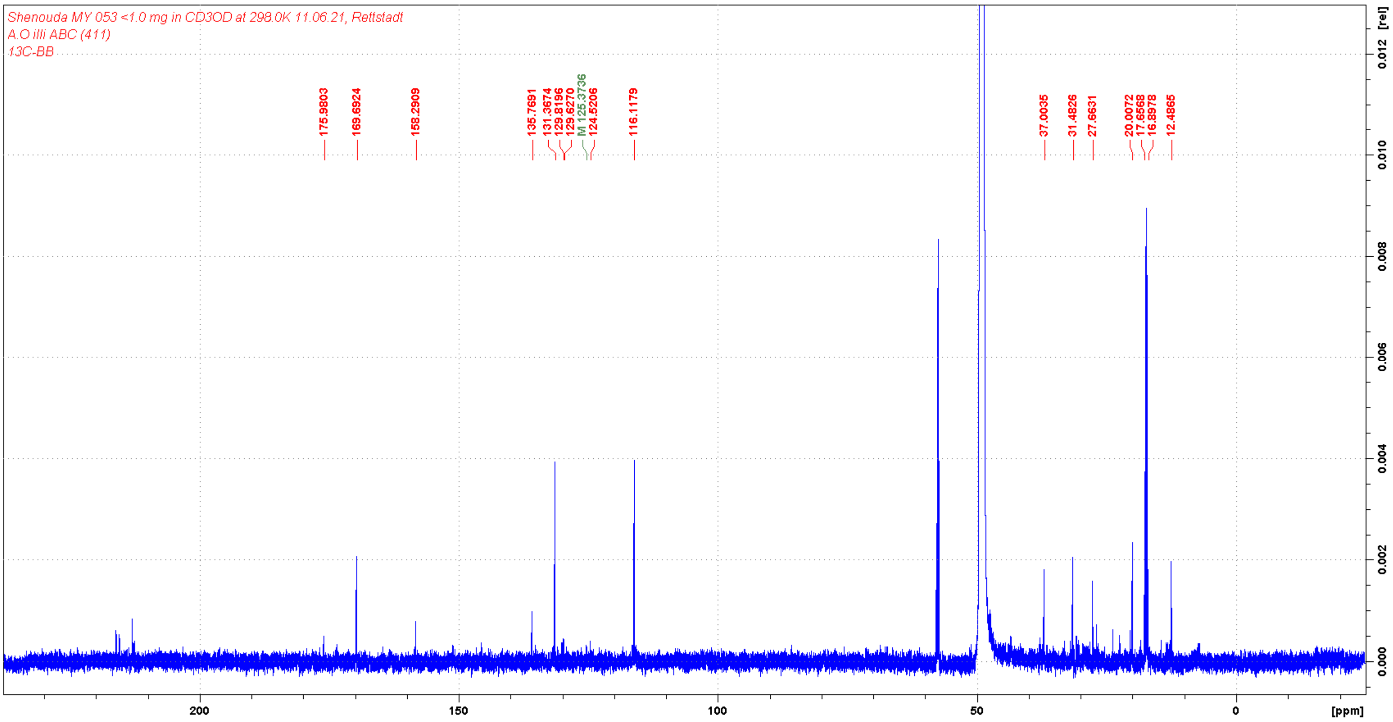

Figure S8.22 ^{13}C-NMR spectrum of compound **14** (125 MHz, CD_3OD).

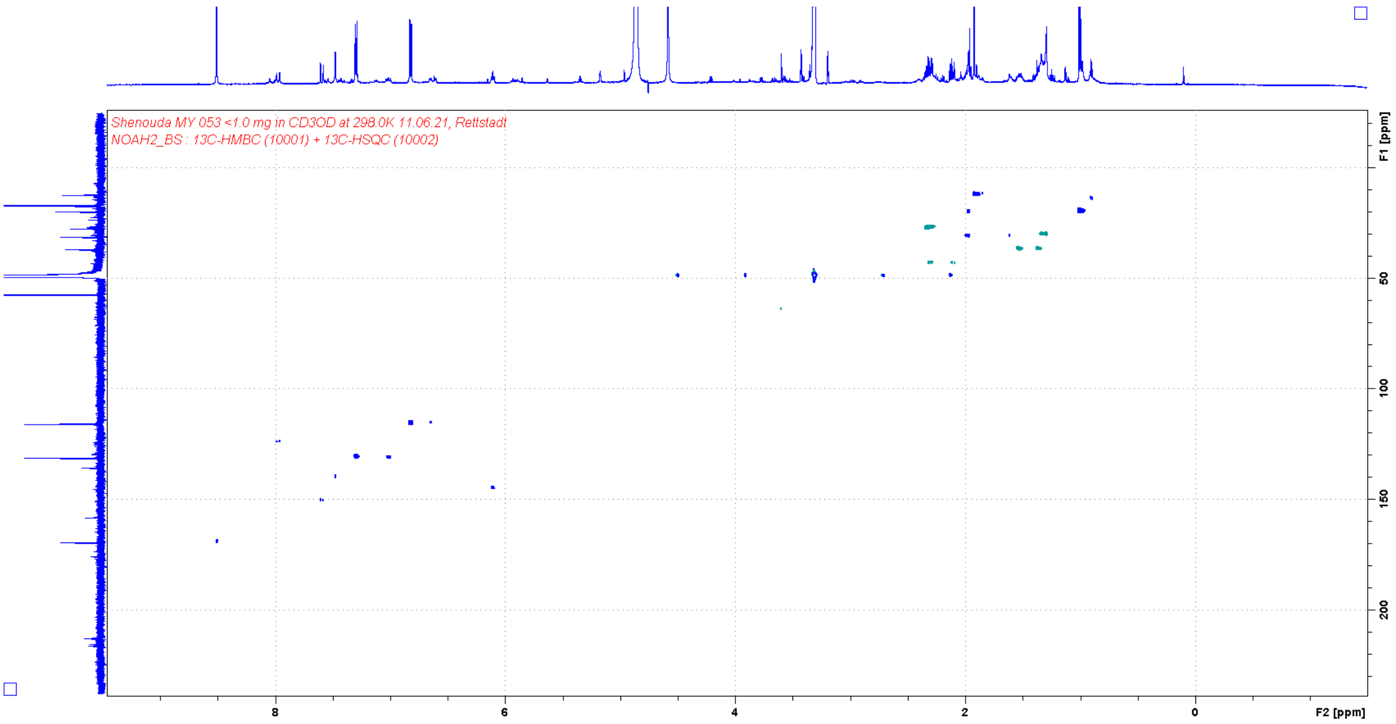

Figure S8.23 HSQC spectrum of compound **14**.

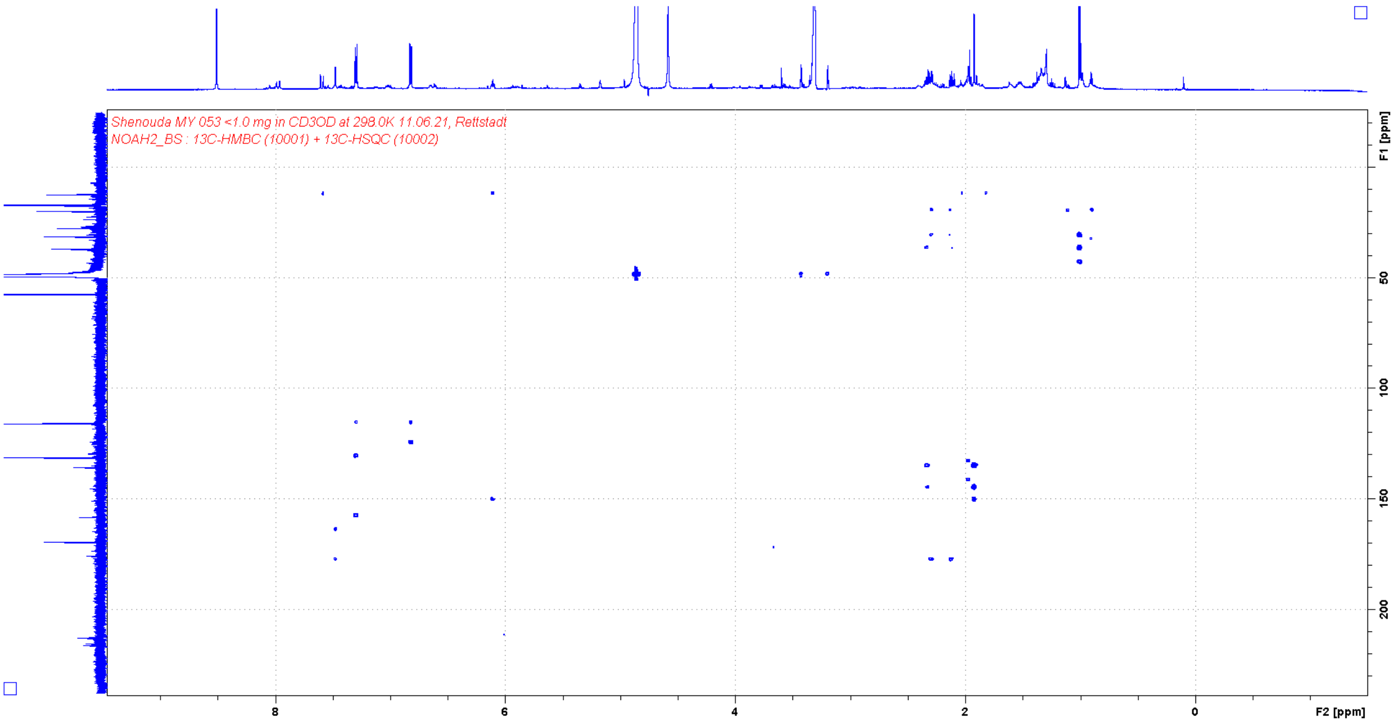

Figure S8.24 HMBC spectrum of compound **14**.

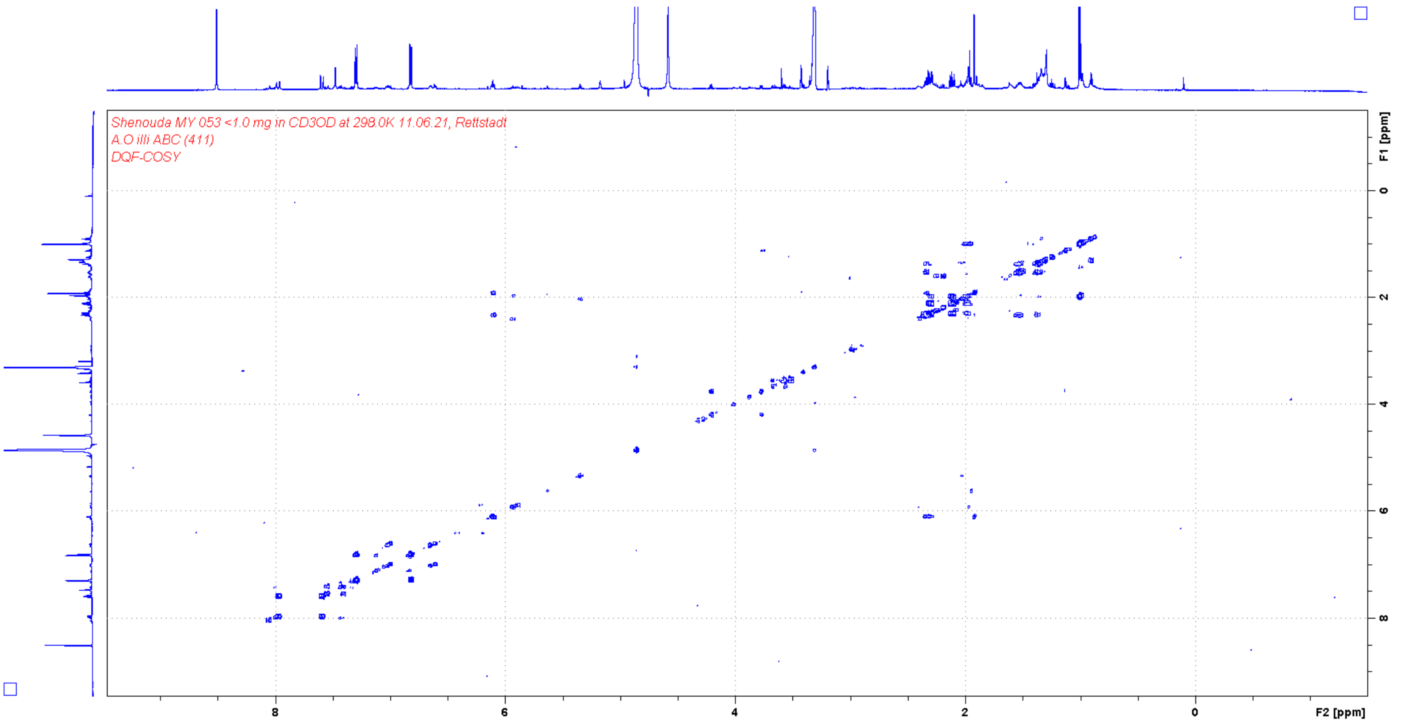

Figure S8.25 COSY spectrum of compound **14**.

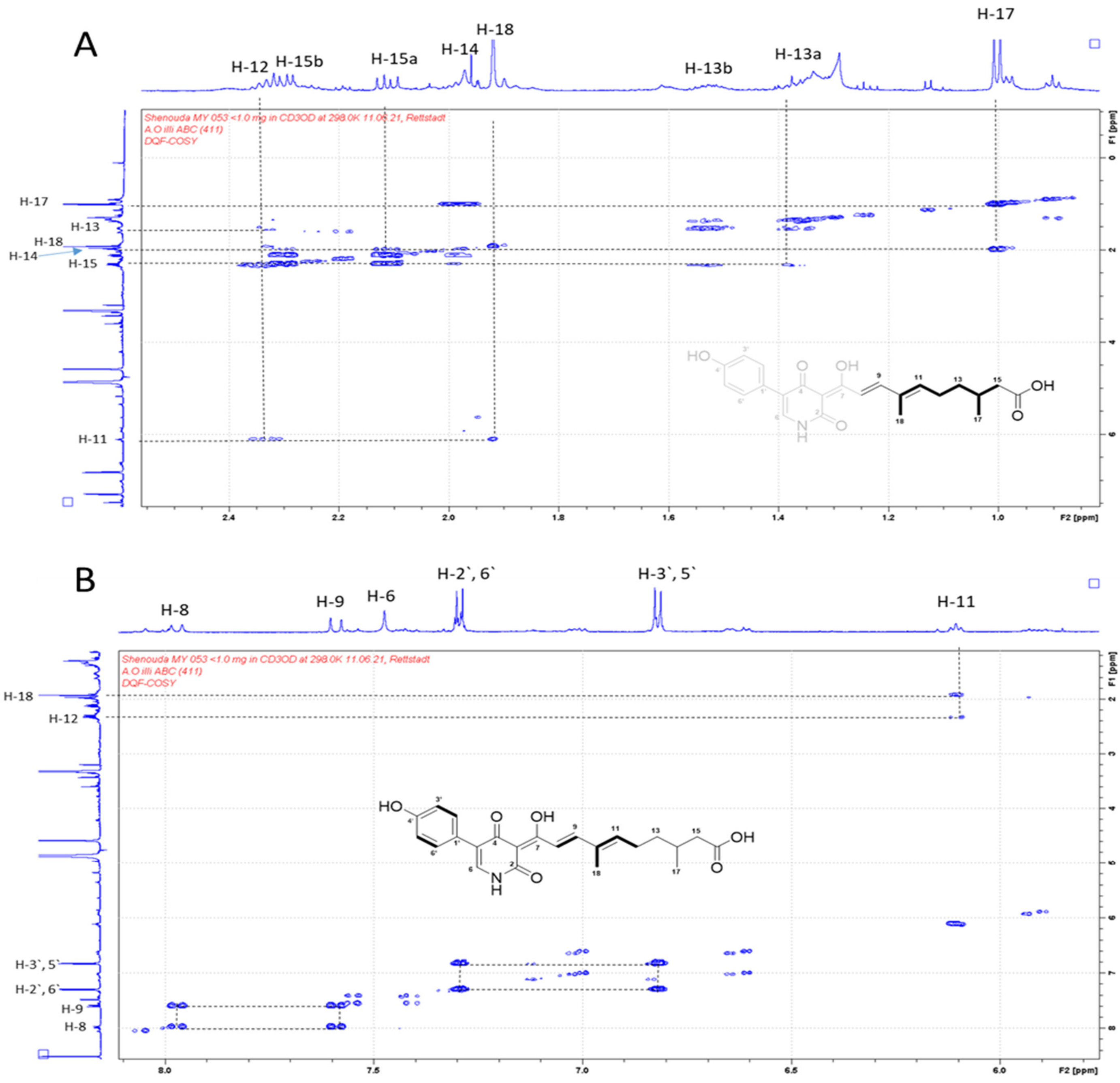

Figure S8.26 COSY correlations of compound **14**.

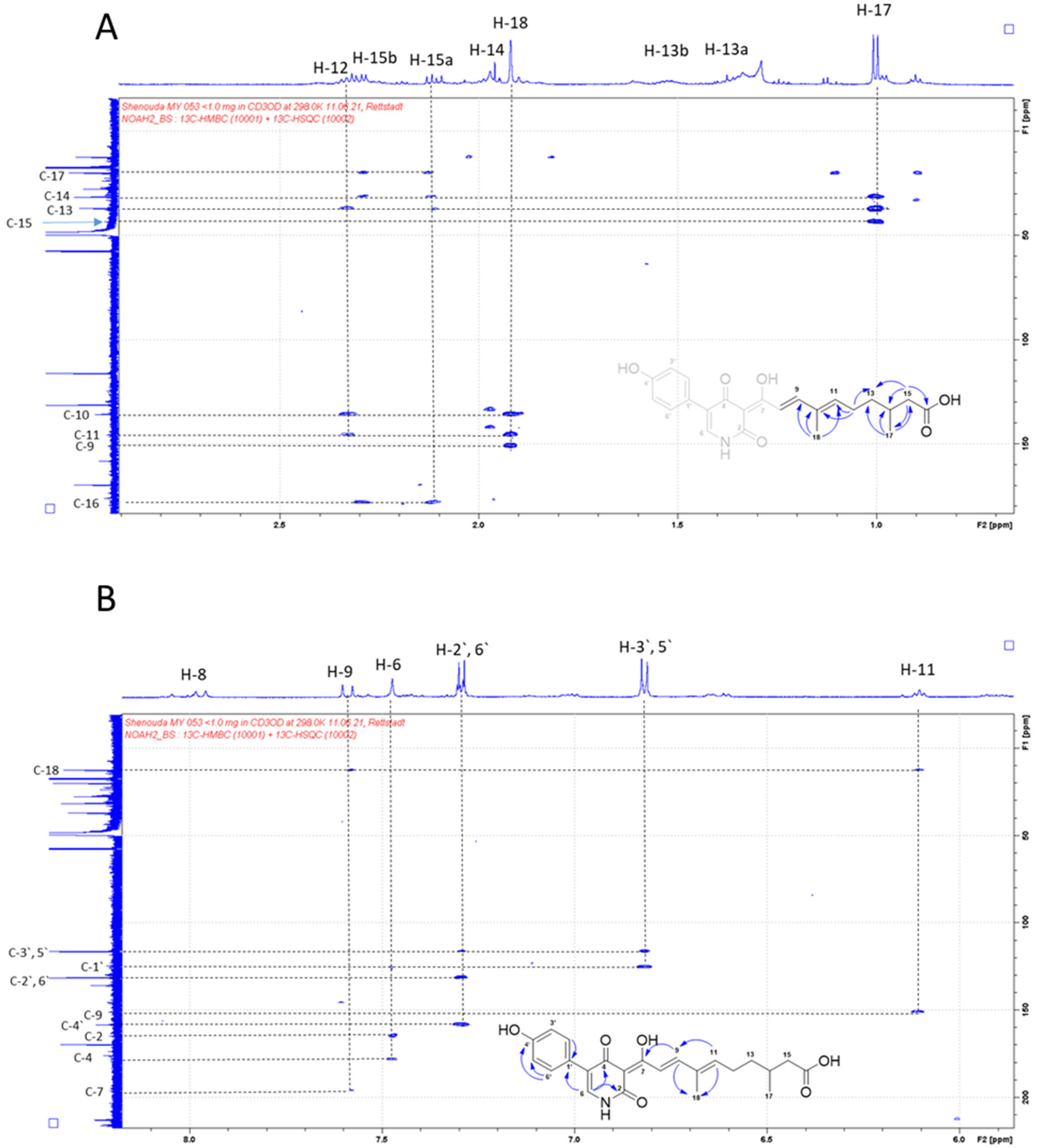

Figure S8.27 HMBC correlations of compound **14**.

Compound 15

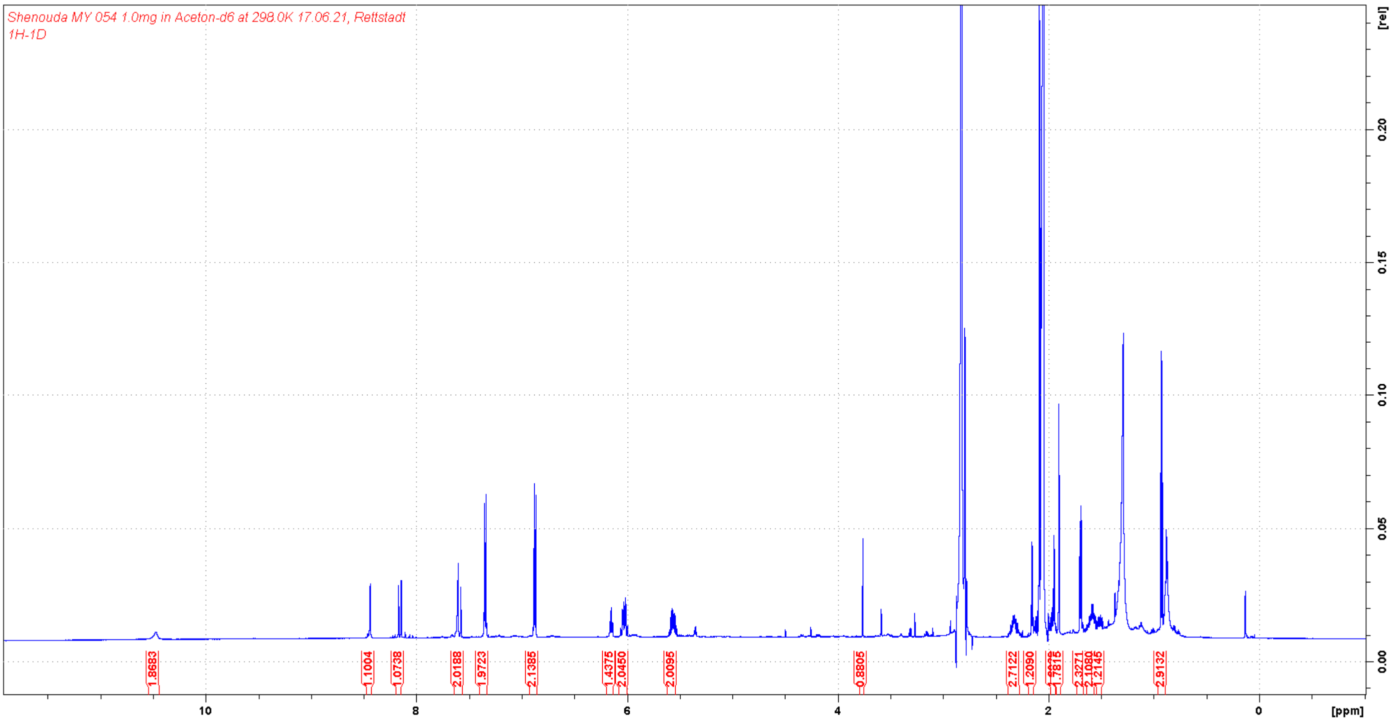

Figure S8.28 ^{1}H-NMR spectrum of compound **15** (500 MHz, Acetone-d_6).

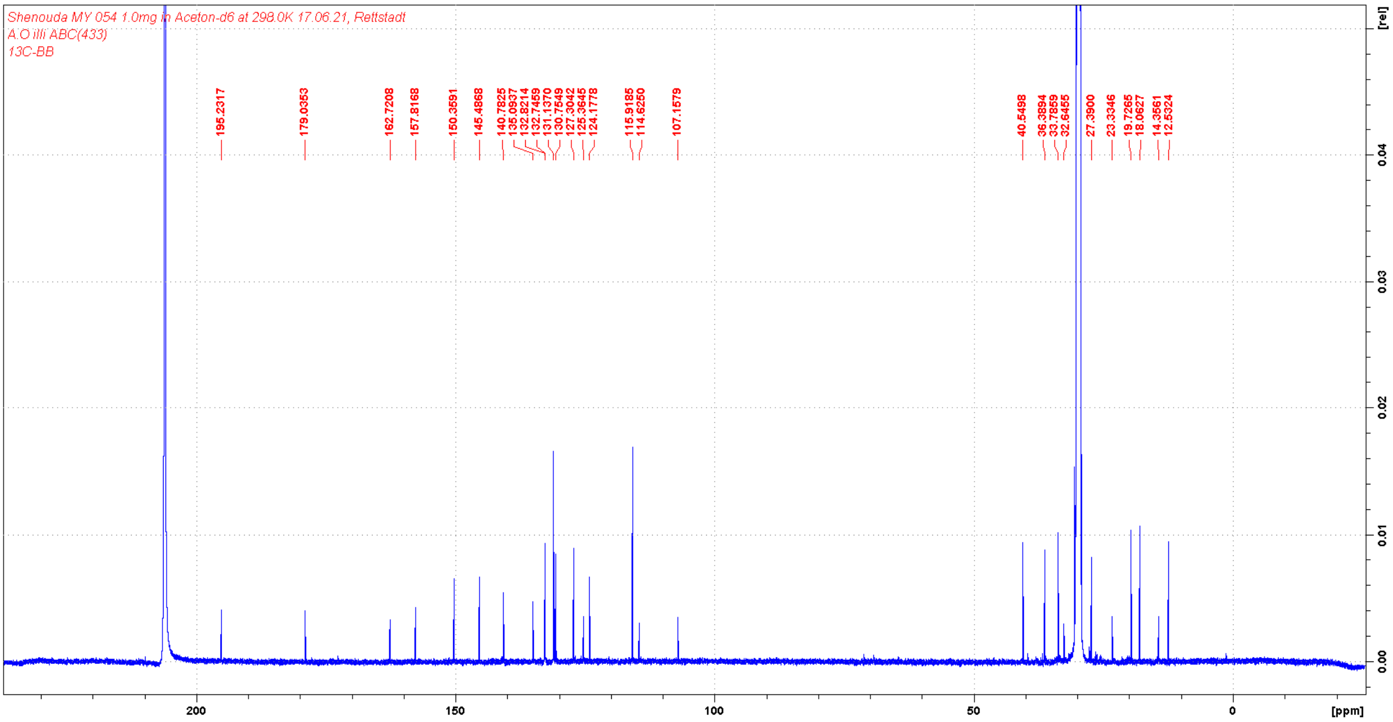

Figure S8.29 ^{13}C-NMR spectrum of compound **15** (125 MHz, Acetone-d_6).

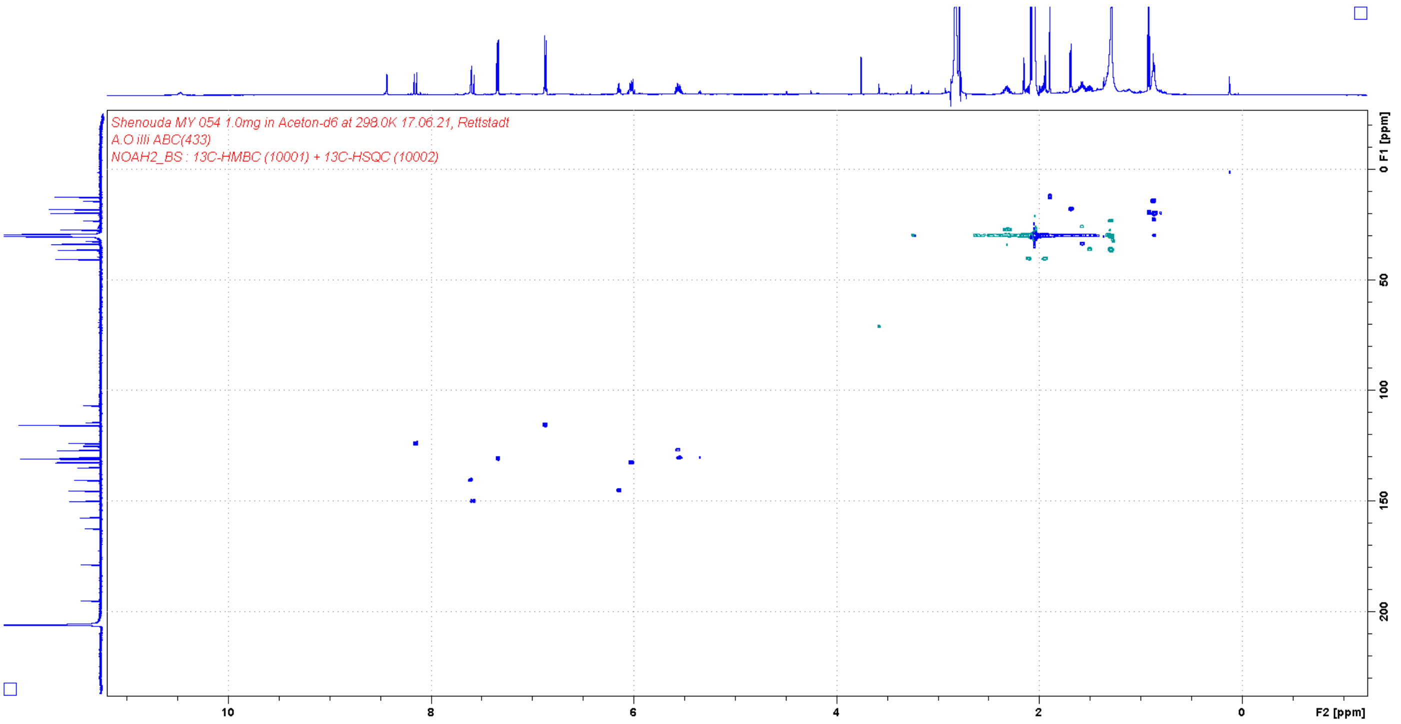

Figure S8.30 HSQC spectrum of compound **15** (Acetone-d_6).

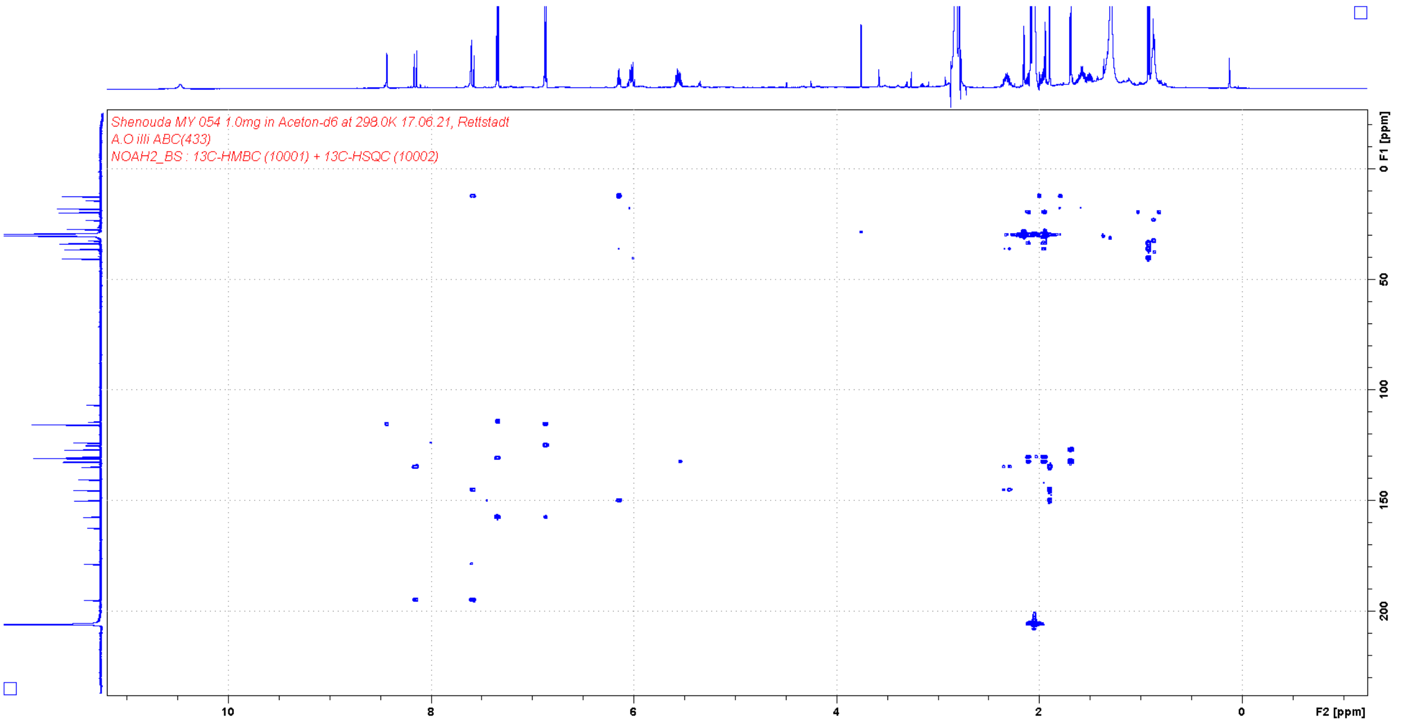

Figure S8.31 HMBC spectrum of compound **15** (Acetone-d_6).

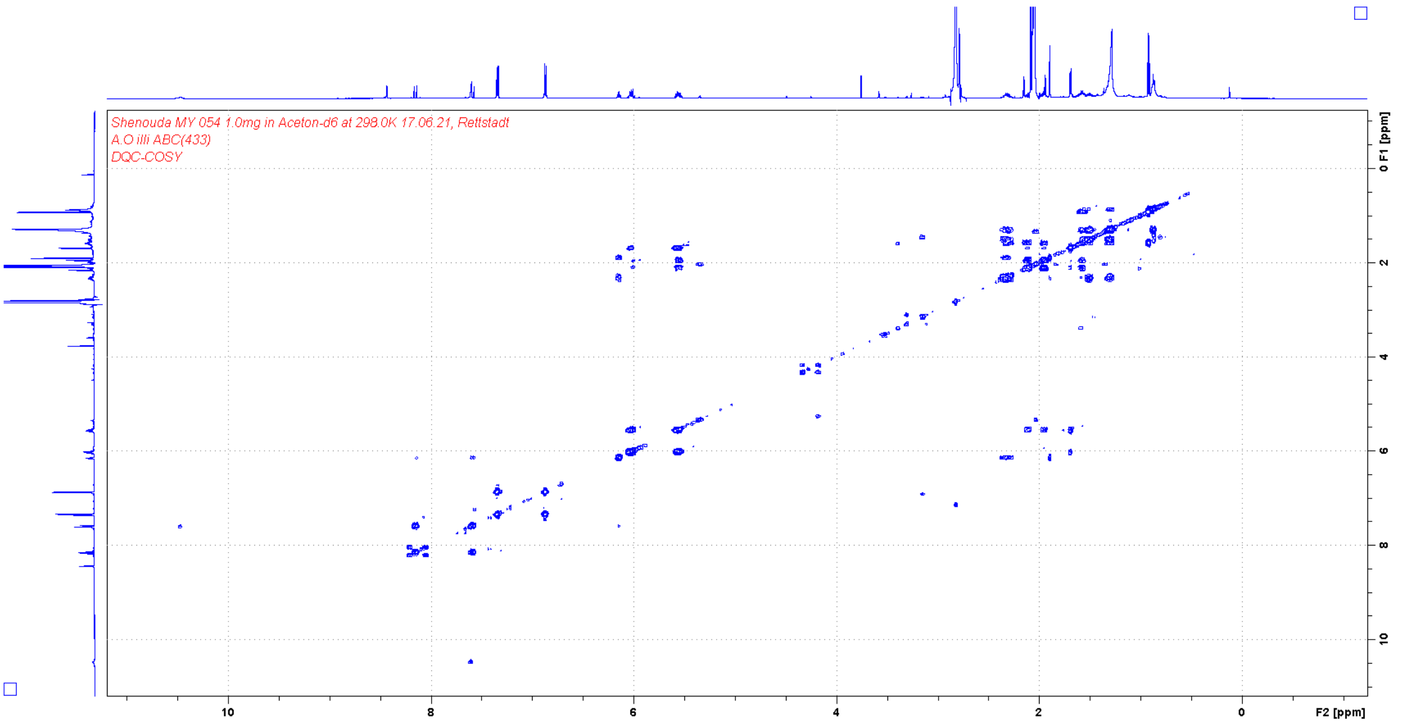

Figure S8.32 COSY spectrum of compound **15** (Acetone-d_6).

Compound 16

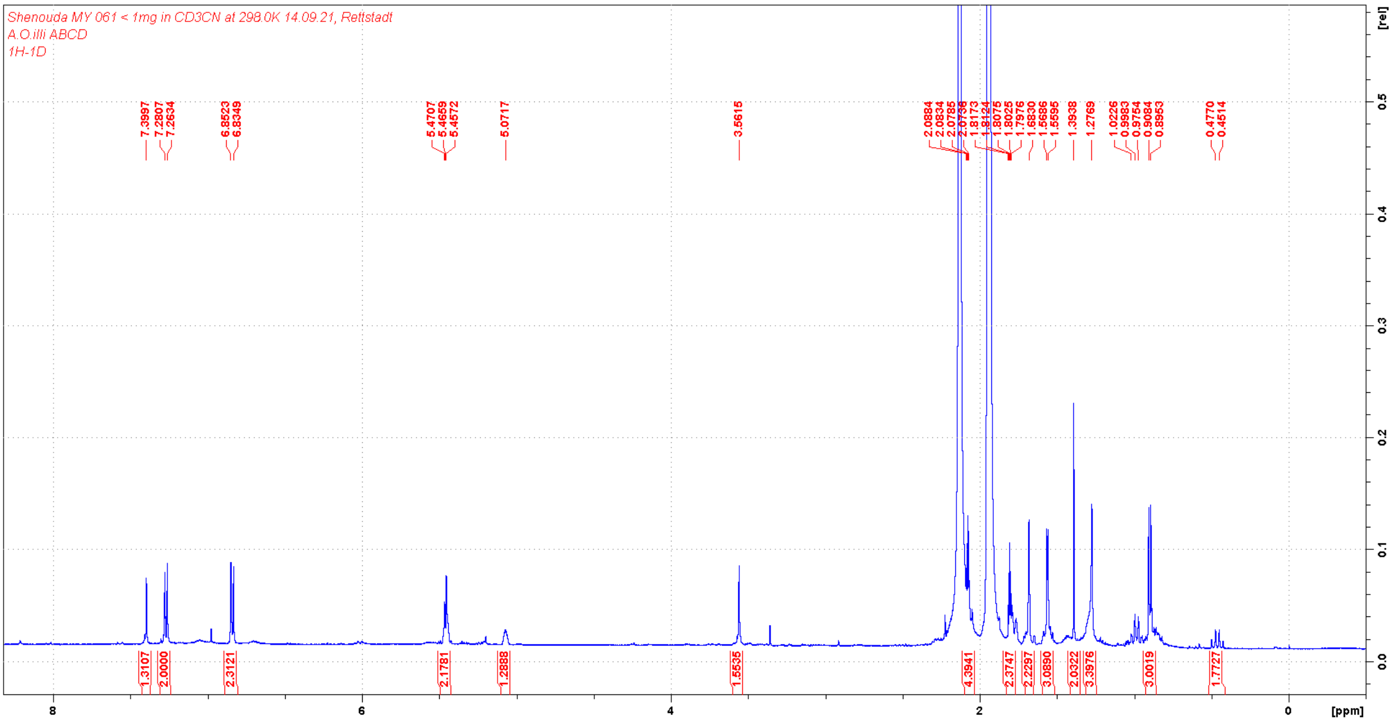

Figure S8.33 ^{1}H-NMR spectrum of compound **16** (500 MHz, Acetonitrile-d$_3$).

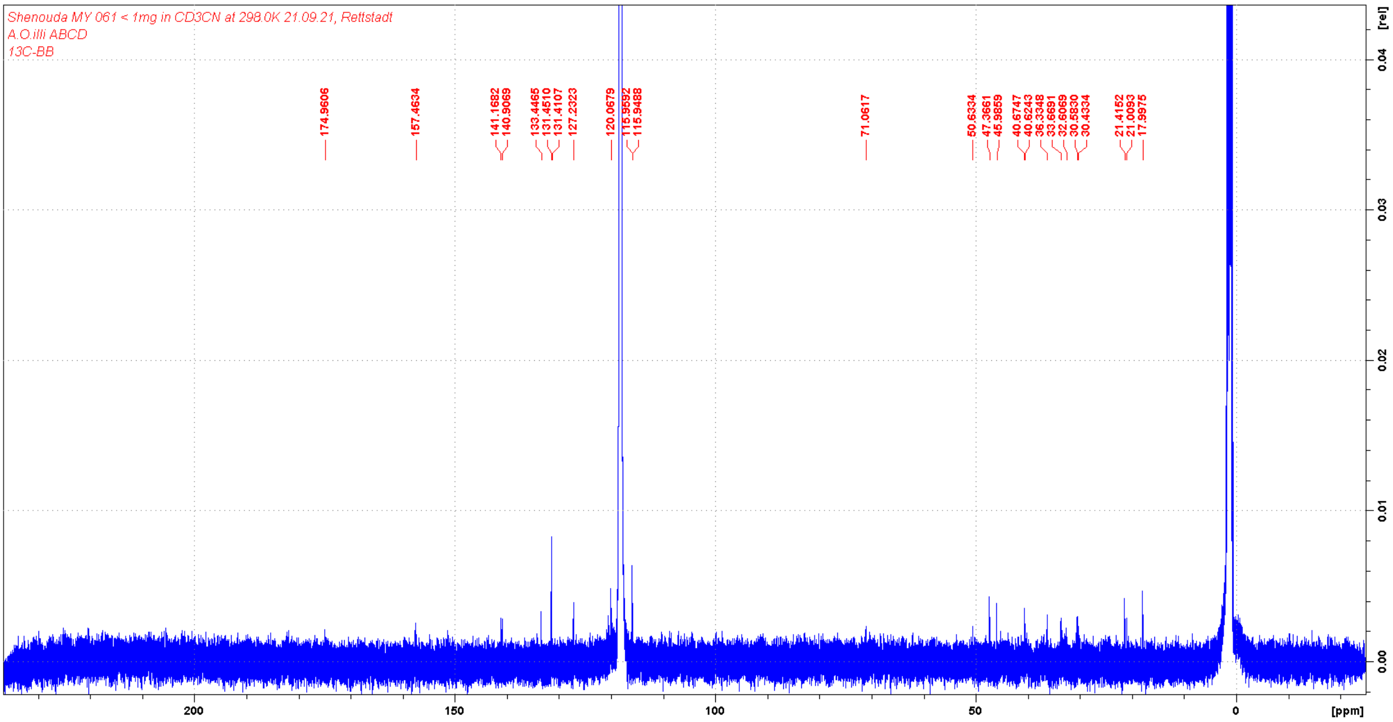

Figure S8.34 ^{13}C-NMR spectrum of compound **16** (125 MHz, Acetonitrile-d$_3$).

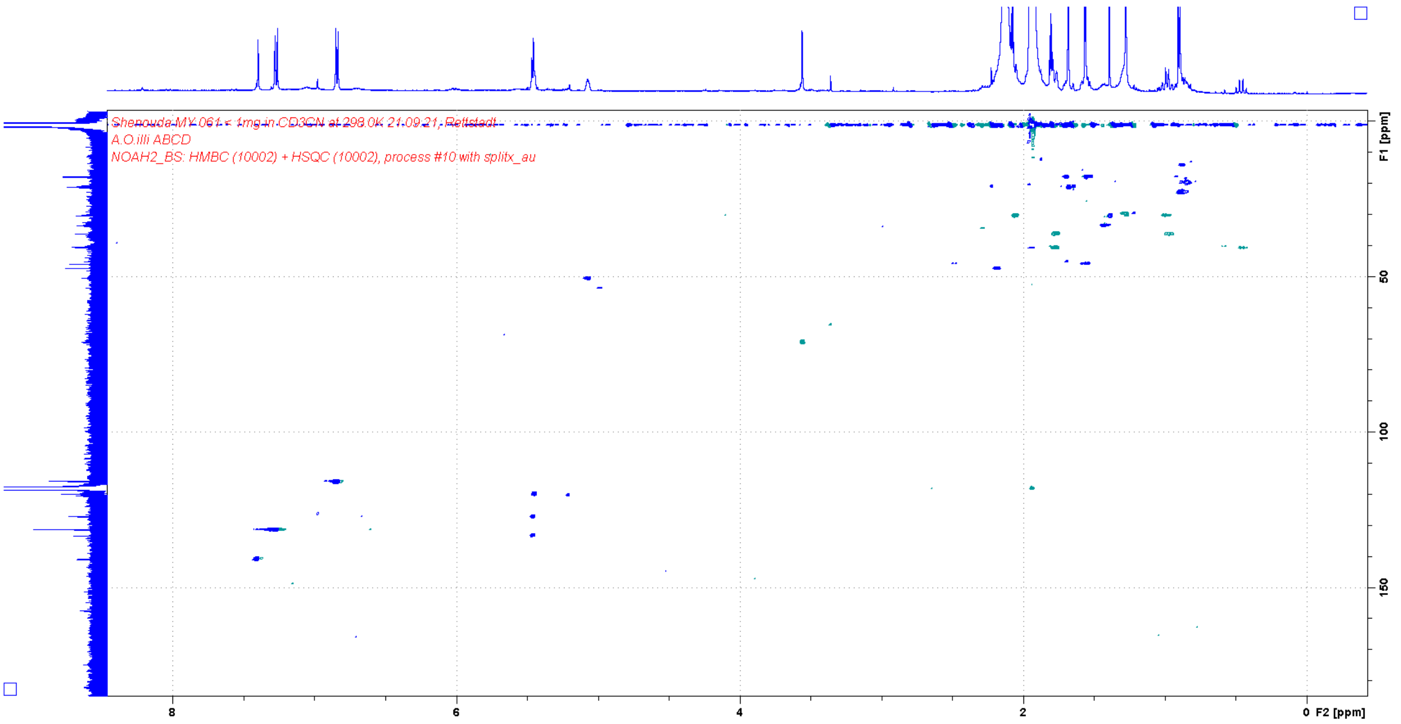

Figure S8.35 HSQC spectrum of compound **16** (Acetonitrile-d_3).

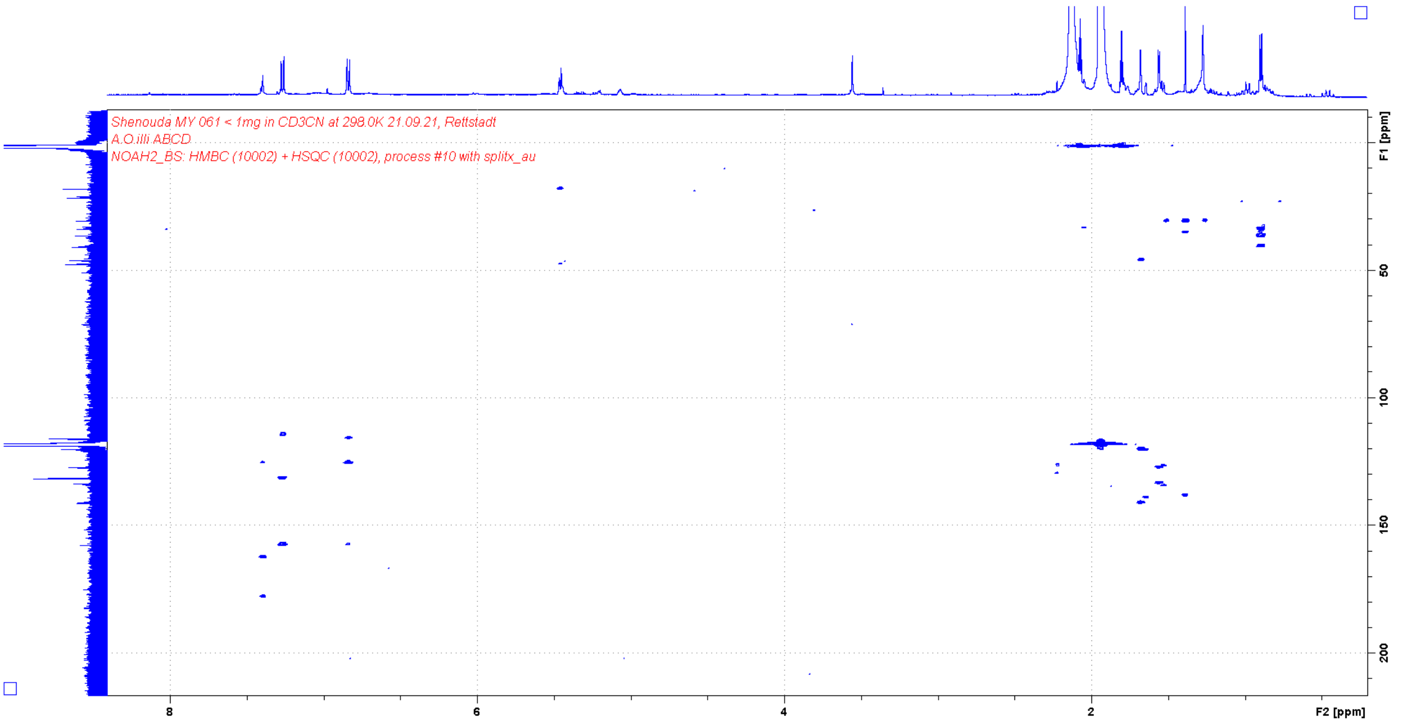

Figure S8.36 HMBC spectrum of compound **16** (Acetonitrile-d_3).

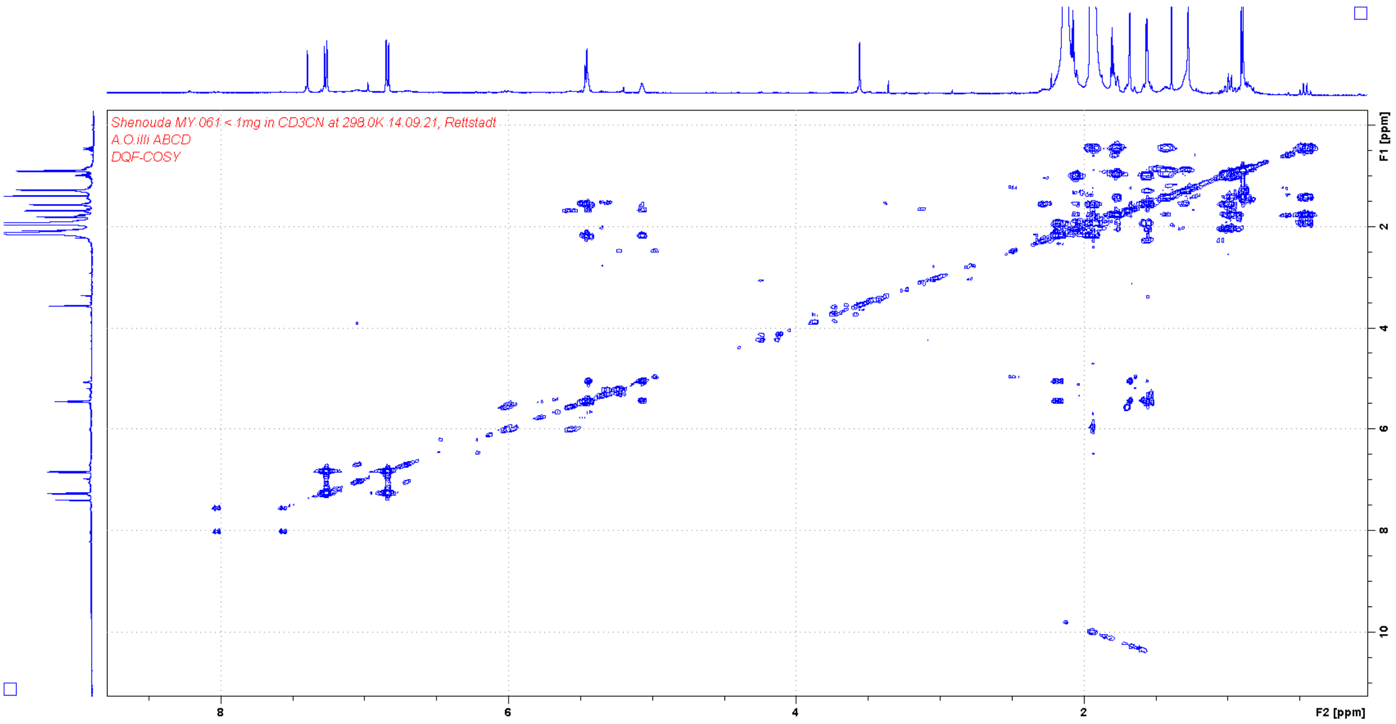

Figure S8.37 COSY spectrum of compound **16** (Acetonitrile-d_3).

Compound 10

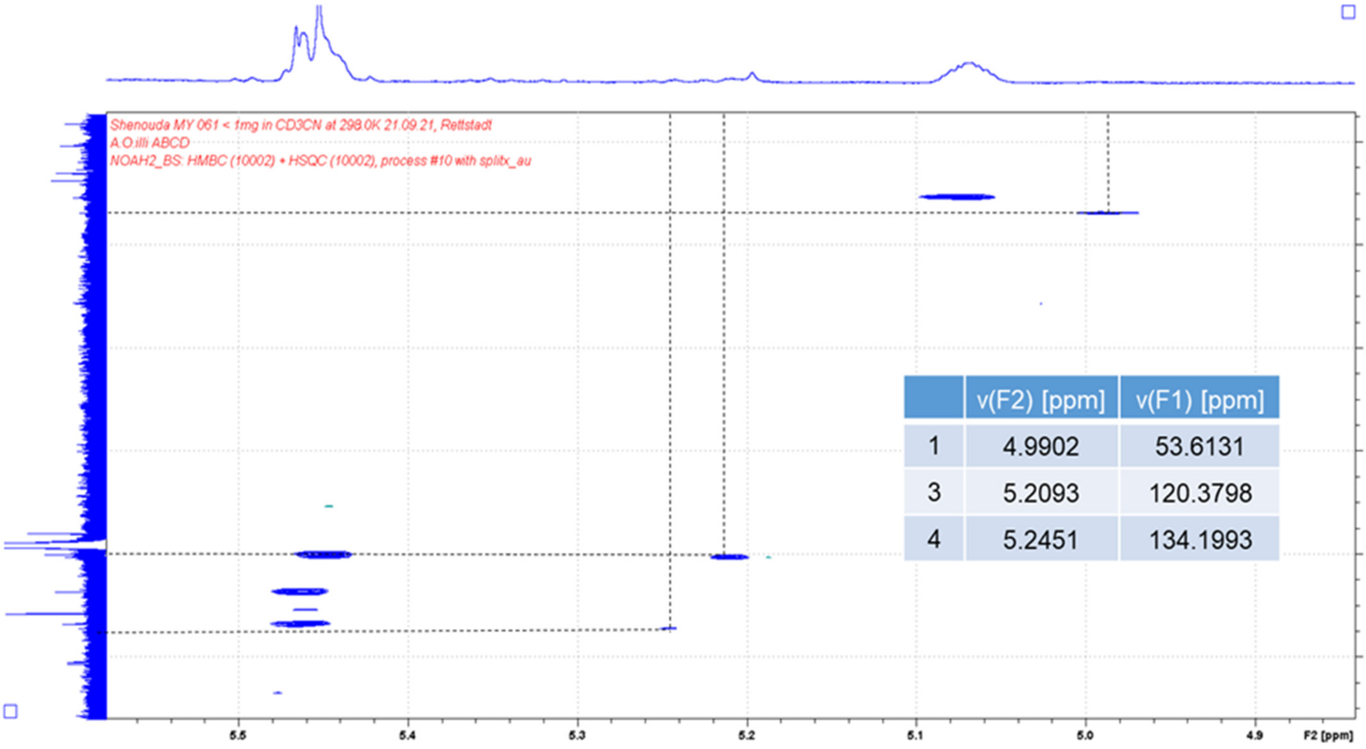

	ν(F2) [ppm]	ν(F1) [ppm]
1	4.9902	53.6131
3	5.2093	120.3798
4	5.2451	134.1993

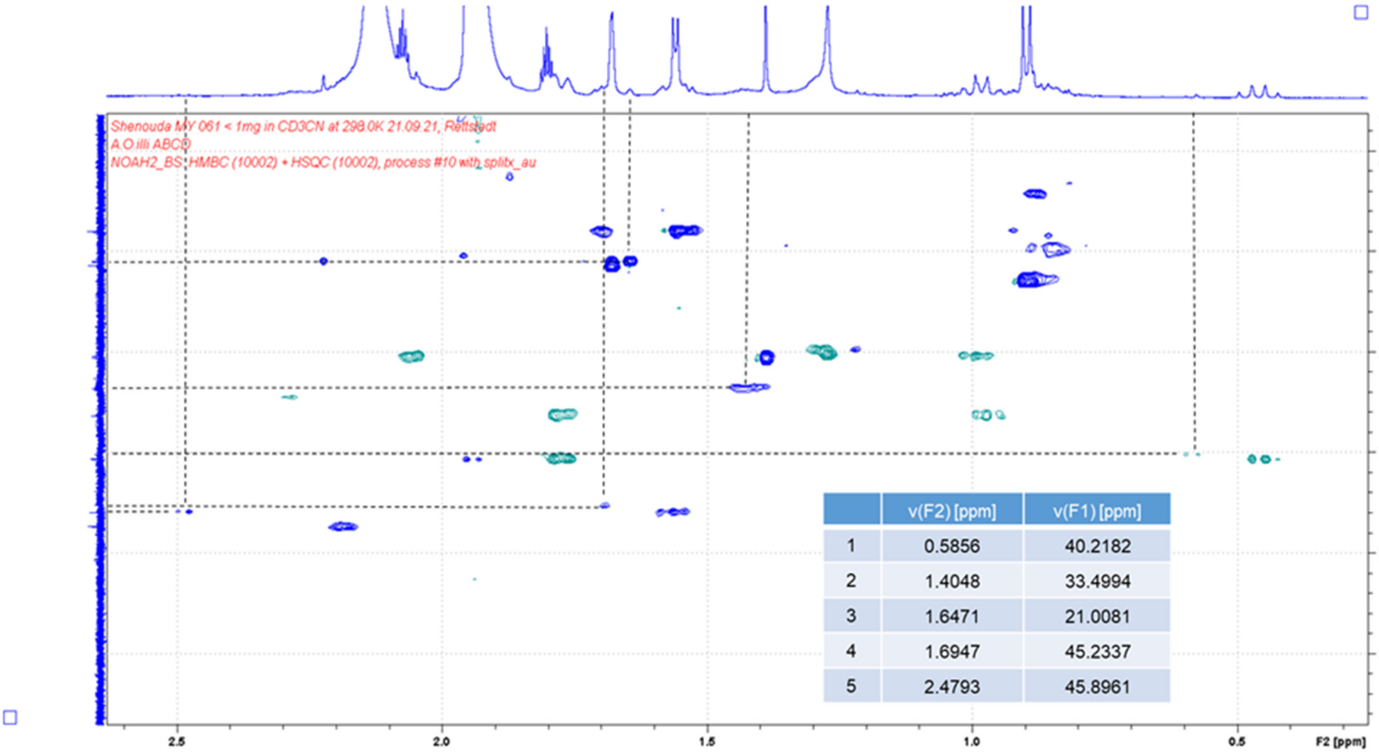

	ν(F2) [ppm]	ν(F1) [ppm]
1	0.5856	40.2182
2	1.4048	33.4994
3	1.6471	21.0081
4	1.6947	45.2337
5	2.4793	45.8961

Figure S8.38 HSQC spectrum showing minor peaks corresponding to ilicicolin-H **10**.

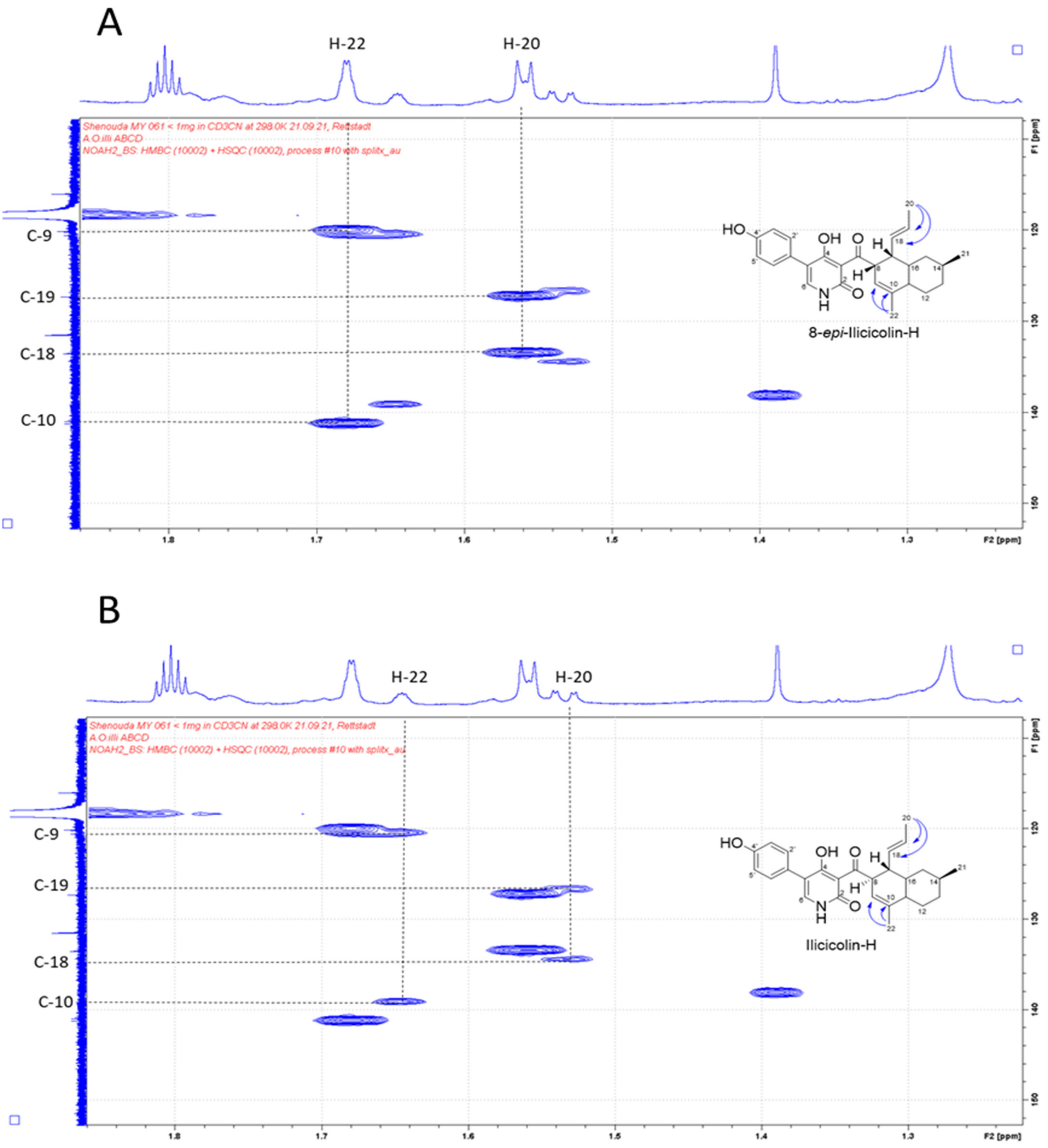

Figure S8.39 HMBC correlation of the compound with nominal mass 433 isolated from *A. oryzae* trasnformants expressing the four genes from the investigated BGC: **A**, HMBC spectrum showing major peaks corresponding to the major compound 8-*epi*-ilicicolin-H **16**; **B**, HMBC spectrum showing minor peaks corresponding to the minor compound ilicicolin-H **10**.

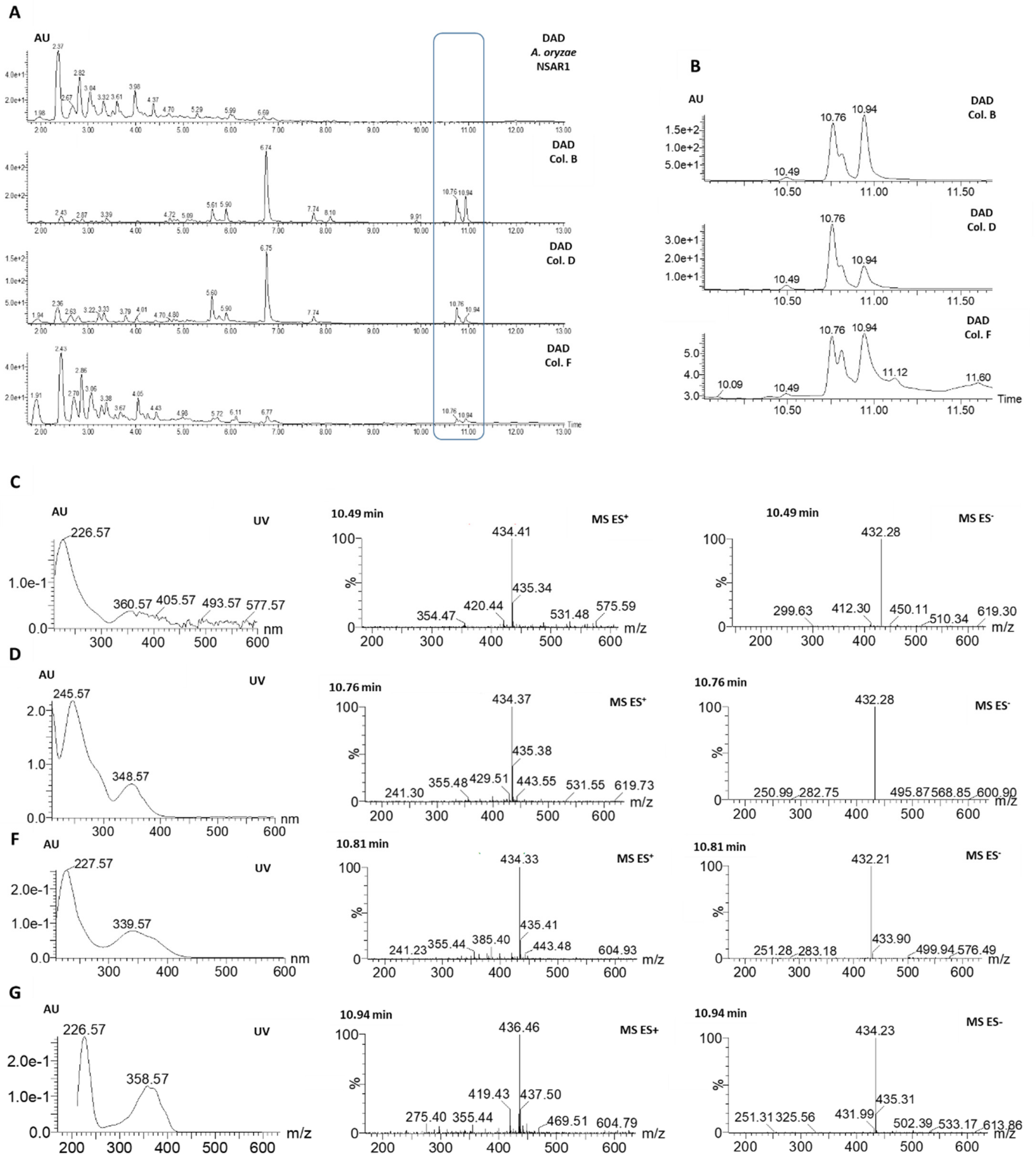

Figure S8.40 Co-expression of four genes in *A. oryzae* NSAR1: **A-B**, LCMS chromatograms of different colonies of *A. oryzae* + *PKS/NRPS58285·ER58289·P45058953* against *A. oryzae* NSAR1 as the WT; **C-G**, UV, ES+ and ES- chromatograms of the four compounds produced by *A. oryzae* pTYGS·*argB·triliA·triliB·triliC·triliD* transformants.

9. HRMS data

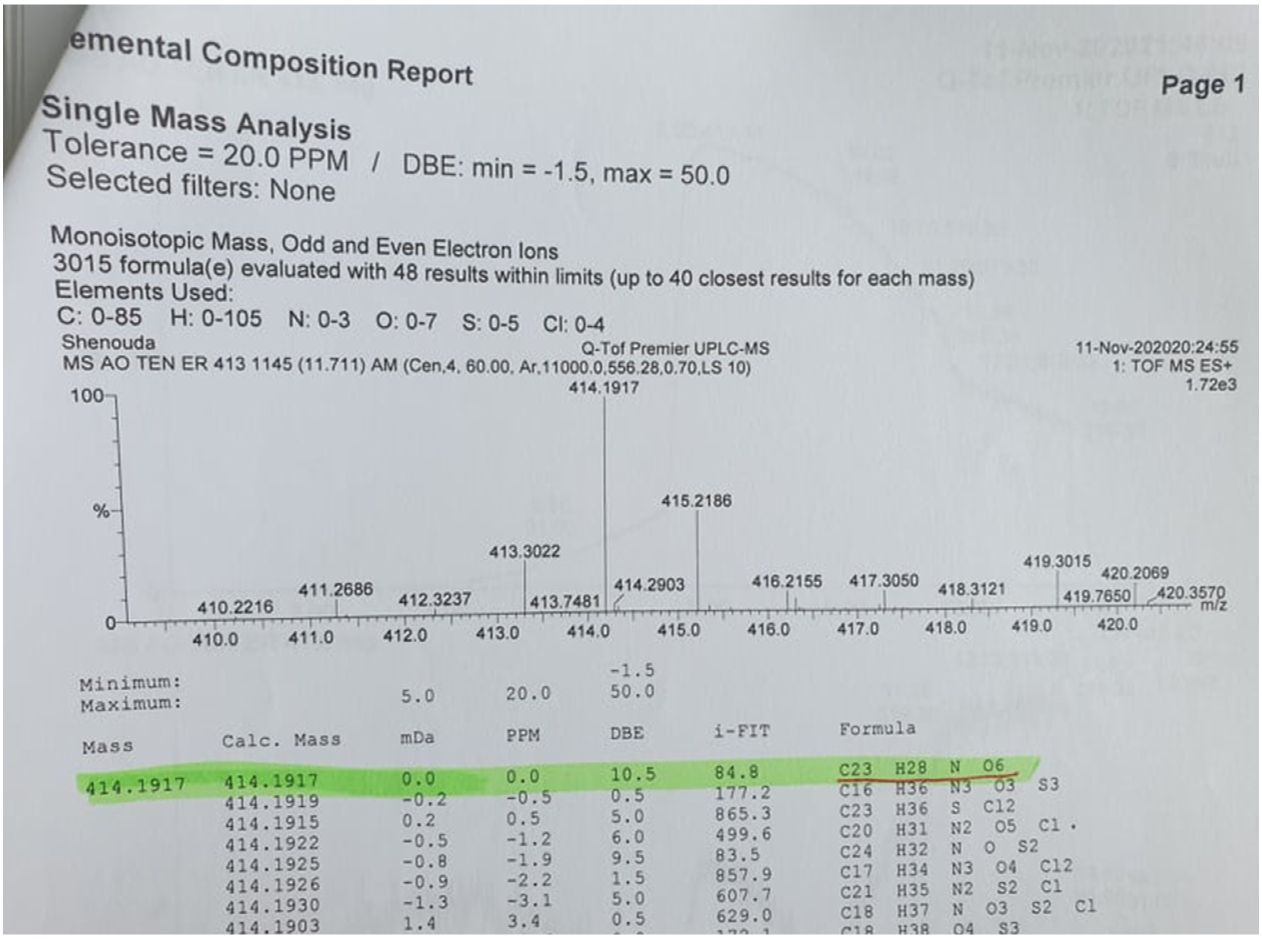

Mass	Calc. Mass	mDa	PPM	DBE	i-FIT	Formula
414.1917	414.1917	0.0	0.0	10.5	84.8	C23 H28 N O6
	414.1919	-0.2	-0.5	0.5	177.2	C16 H36 N3 O3 S3
	414.1915	0.2	0.5	5.0	865.3	C23 H36 S Cl2
	414.1922	-0.5	-1.2	6.0	499.6	C20 H31 N2 O5 Cl
	414.1925	-0.8	-1.9	9.5	83.5	C24 H32 N O S2
	414.1926	-0.9	-2.2	1.5	857.9	C17 H34 N3 O4 Cl2
	414.1930	-1.3	-3.1	5.0	607.7	C21 H35 N2 S2 Cl
	414.1903	1.4	3.4	0.5	629.0	C18 H37 N O3 S2 Cl
	[illegible]	[illegible]	[illegible]	[illegible]	[illegible]	C18 H38 O4 S3

Figure S9.1. HRMS anaylsis of compound **11**.

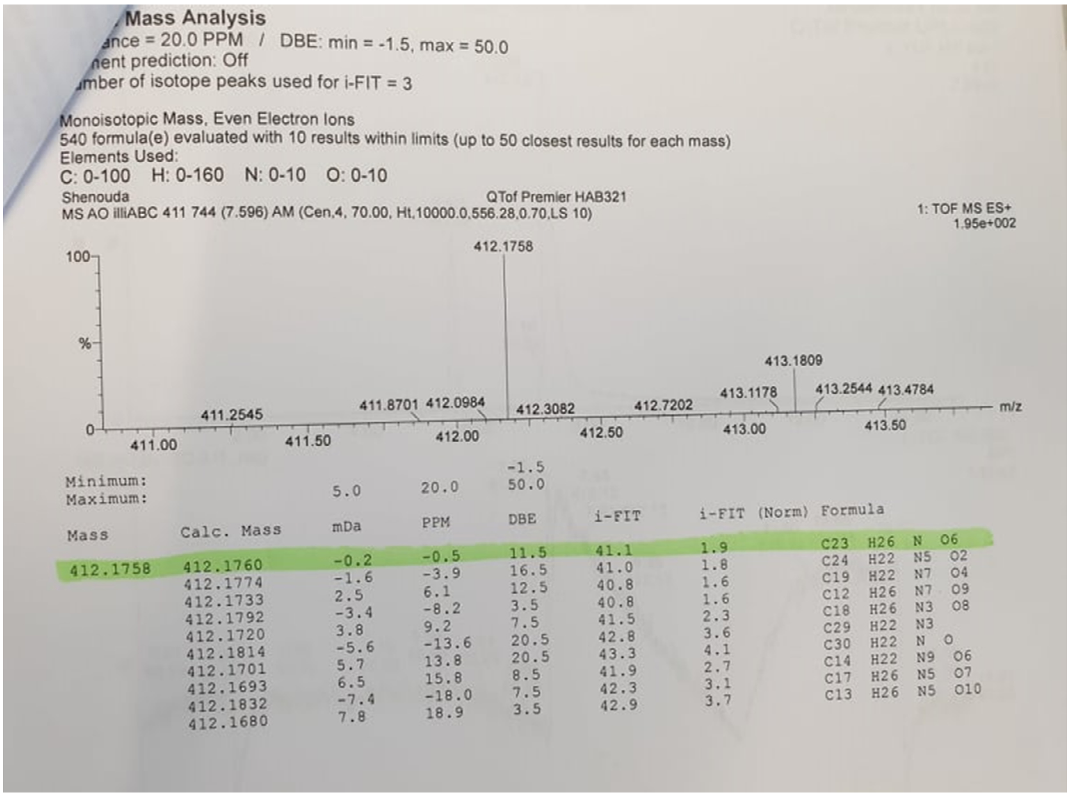
Mass Analysis
ance = 20.0 PPM / DBE: min = -1.5, max = 50.0
ent prediction: Off
mber of isotope peaks used for i-FIT = 3

Monoisotopic Mass, Even Electron Ions
540 formula(e) evaluated with 10 results within limits (up to 50 closest results for each mass)
Elements Used:
C: 0-100 H: 0-160 N: 0-10 O: 0-10
Shenouda QTof Premier HAB321
MS AO illiABC 411 744 (7.596) AM (Cen,4, 70.00, Ht,10000.0,556.28,0.70,LS 10) 1: TOF MS ES+ 1.95e+002

Minimum:				-1.5			
Maximum:		5.0	20.0	50.0			
Mass	Calc. Mass	mDa	PPM	DBE	i-FIT	i-FIT (Norm)	Formula
412.1758	412.1760	-0.2	-0.5	11.5	41.1	1.9	C23 H26 N O6
	412.1774	-1.6	-3.9	16.5	41.0	1.8	C24 H22 N5 O2
	412.1733	2.5	6.1	12.5	40.8	1.6	C19 H22 N7 O4
	412.1792	-3.4	-8.2	3.5	40.8	1.6	C12 H26 N7 O9
	412.1720	3.8	9.2	7.5	41.5	2.3	C18 H26 N3 O8
	412.1814	-5.6	-13.6	20.5	42.8	3.6	C29 H22 N3
	412.1701	5.7	13.8	20.5	43.3	4.1	C30 H22 N O
	412.1693	6.5	15.8	8.5	41.9	2.7	C14 H22 N9 O6
	412.1832	-7.4	-18.0	7.5	42.3	3.1	C17 H26 N5 O7
	412.1680	7.8	18.9	3.5	42.9	3.7	C13 H26 N5 O10

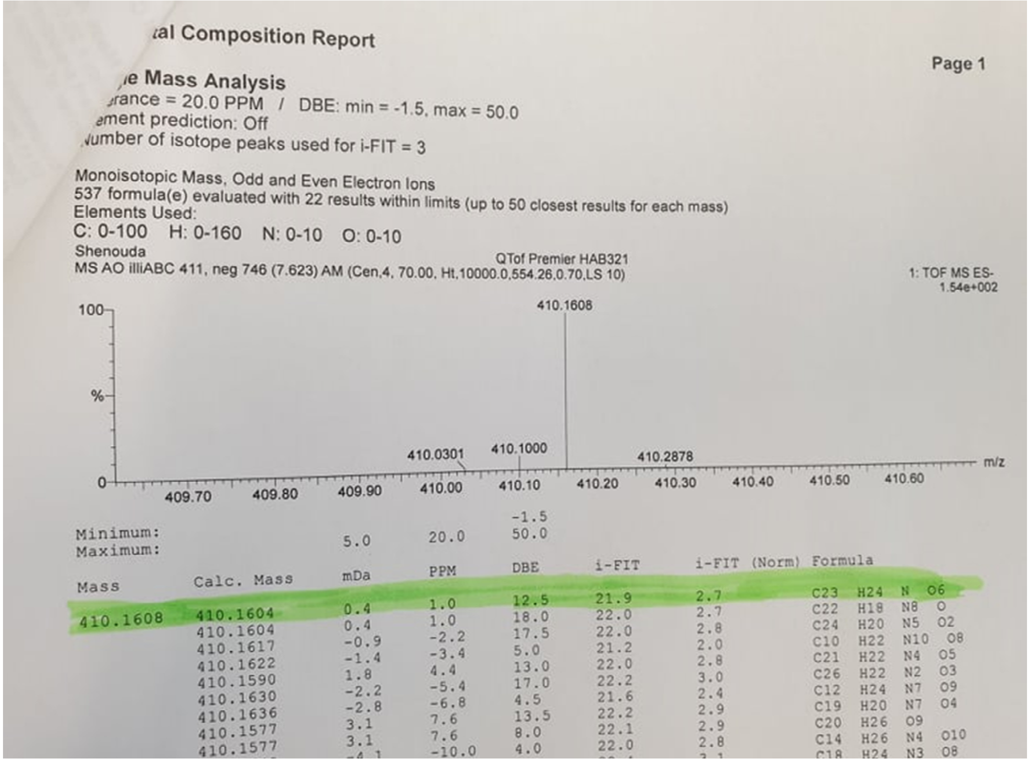
al Composition Report
Page 1
e Mass Analysis
rance = 20.0 PPM / DBE: min = -1.5, max = 50.0
ement prediction: Off
Number of isotope peaks used for i-FIT = 3

Monoisotopic Mass, Odd and Even Electron Ions
537 formula(e) evaluated with 22 results within limits (up to 50 closest results for each mass)
Elements Used:
C: 0-100 H: 0-160 N: 0-10 O: 0-10
Shenouda QTof Premier HAB321
MS AO illiABC 411, neg 746 (7.623) AM (Cen,4, 70.00, Ht,10000.0,554.26,0.70,LS 10) 1: TOF MS ES- 1.54e+002

Minimum:				-1.5			
Maximum:		5.0	20.0	50.0			
Mass	Calc. Mass	mDa	PPM	DBE	i-FIT	i-FIT (Norm)	Formula
410.1608	410.1604	0.4	1.0	12.5	21.9	2.7	C23 H24 N O6
	410.1604	0.4	1.0	18.0	22.0	2.7	C22 H18 N8 O
	410.1617	-0.9	-2.2	17.5	22.0	2.8	C24 H20 N5 O2
	410.1622	-1.4	-3.4	5.0	21.2	2.0	C10 H22 N10 O8
	410.1590	1.8	4.4	13.0	22.0	2.8	C21 H22 N4 O5
	410.1630	-2.2	-5.4	17.0	22.2	3.0	C26 H22 N2 O3
	410.1636	-2.8	-6.8	4.5	21.6	2.4	C12 H24 N7 O9
	410.1577	3.1	7.6	13.5	22.2	2.9	C19 H20 N7 O4
	410.1577	3.1	7.6	8.0	22.1	2.9	C20 H26 O9
	[illegible]	-4.1	-10.0	4.0	22.0	2.8	C14 H26 N4 O10
	[illegible]	[illegible]	[illegible]	[illegible]	[illegible]	3.1	C18 H24 N3 O8

Figure S9.2. HRMS analysis of compound **14** (ES+ and ES-).

...mposition Report

Page 1

...gle Mass Analysis
...lerance = 20.0 PPM / DBE: min = -1.5, max = 50.0
...elected filters: None

Monoisotopic Mass, Odd and Even Electron Ions
3628 formula(e) evaluated with 63 results within limits (up to 40 closest results for each mass)
Elements Used:
C: 0-85 H: 0-105 N: 0-3 O: 0-7 S: 0-5 Cl: 0-4
Shenouda
Q-Tof Premier UPLC-MS
11-Nov-202020:40:30
MS AO TEN ER 465 671 (6.860) AM (Cen,4, 60.00, Ar,11000.0,556.28,0.70,LS 10); Cm (670:680)
1: TOF MS ES+
1.94e3

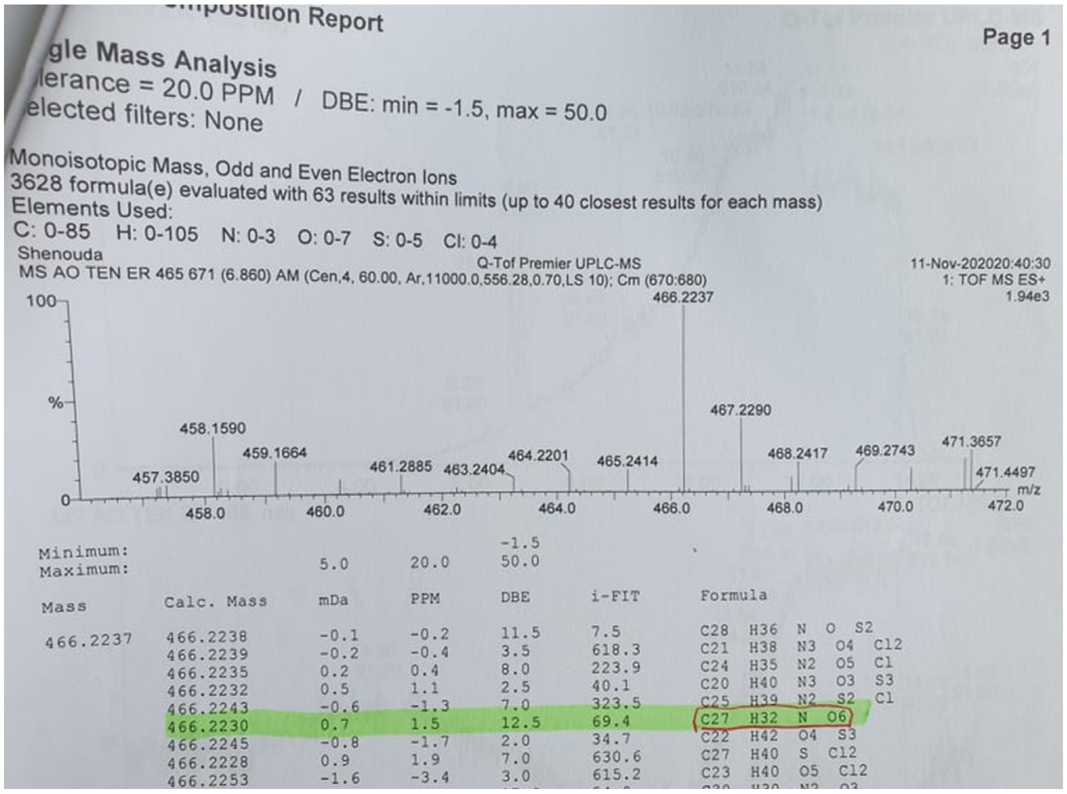

Minimum:				-1.5		
Maximum:		5.0	20.0	50.0		
Mass	Calc. Mass	mDa	PPM	DBE	i-FIT	Formula
466.2237	466.2238	-0.1	-0.2	11.5	7.5	C28 H36 N O S2
	466.2239	-0.2	-0.4	3.5	618.3	C21 H38 N3 O4 Cl2
	466.2235	0.2	0.4	8.0	223.9	C24 H35 N2 O5 Cl
	466.2232	0.5	1.1	2.5	40.1	C20 H40 N3 O3 S3
	466.2243	-0.6	-1.3	7.0	323.5	C25 H39 N2 S2 Cl
	466.2230	0.7	1.5	12.5	69.4	C27 H32 N O6
	466.2245	-0.8	-1.7	2.0	34.7	C22 H42 O4 S3
	466.2228	0.9	1.9	7.0	630.6	C27 H40 S Cl2
	466.2253	-1.6	-3.4	3.0	615.2	C23 H40 O5 Cl2

Figure S9.3. HRMs analysis of compound **12**.

Elemental Composition Report

Page 1

Single Mass Analysis
Tolerance = 20.0 PPM / DBE: min = -1.5, max = 50.0
Element prediction: Off
Number of isotope peaks used for i-FIT = 3

Monoisotopic Mass, Even Electron Ions
568 formula(e) evaluated with 11 results within limits (up to 50 closest results for each mass)
Elements Used:
C: 0-100 H: 0-160 N: 0-10 O: 0-10
Shenouda
QTof Premier HAB321
MY 054,neg 1082 (11.041) AM (Cen,4, 70.00, Ht,10000.0,554.26,0.70,LS 10)
1: TOF MS ES-
2.24e+002

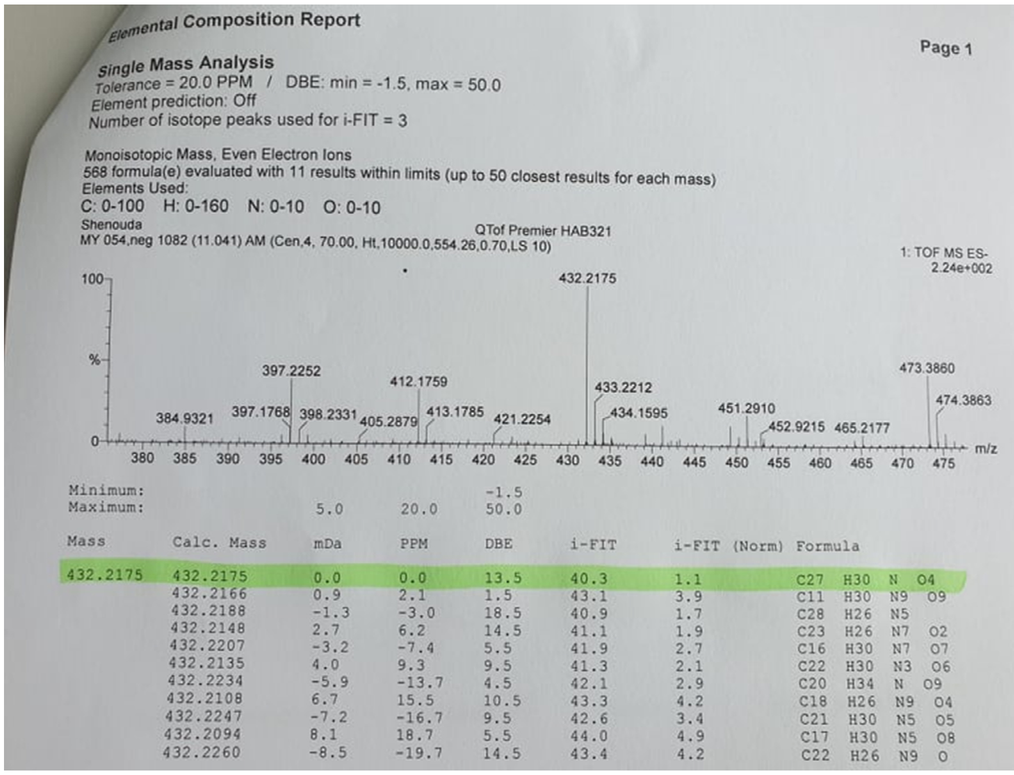

Minimum:				-1.5			
Maximum:		5.0	20.0	50.0			
Mass	Calc. Mass	mDa	PPM	DBE	i-FIT	i-FIT (Norm)	Formula
432.2175	432.2175	0.0	0.0	13.5	40.3	1.1	C27 H30 N O4
	432.2166	0.9	2.1	1.5	43.1	3.9	C11 H30 N9 O9
	432.2188	-1.3	-3.0	18.5	40.9	1.7	C28 H26 N5
	432.2148	2.7	6.2	14.5	41.1	1.9	C23 H26 N7 O2
	432.2207	-3.2	-7.4	5.5	41.9	2.7	C16 H30 N7 O7
	432.2135	4.0	9.3	9.5	41.3	2.1	C22 H30 N3 O6
	432.2234	-5.9	-13.7	4.5	42.1	2.9	C20 H34 N O9
	432.2108	6.7	15.5	10.5	43.3	4.2	C18 H26 N9 O4
	432.2247	-7.2	-16.7	9.5	42.6	3.4	C21 H30 N5 O5
	432.2094	8.1	18.7	5.5	44.0	4.9	C17 H30 N5 O8
	432.2260	-8.5	-19.7	14.5	43.4	4.2	C22 H26 N9 O

Figure S9.4. HRMS analysis of compound **15**.

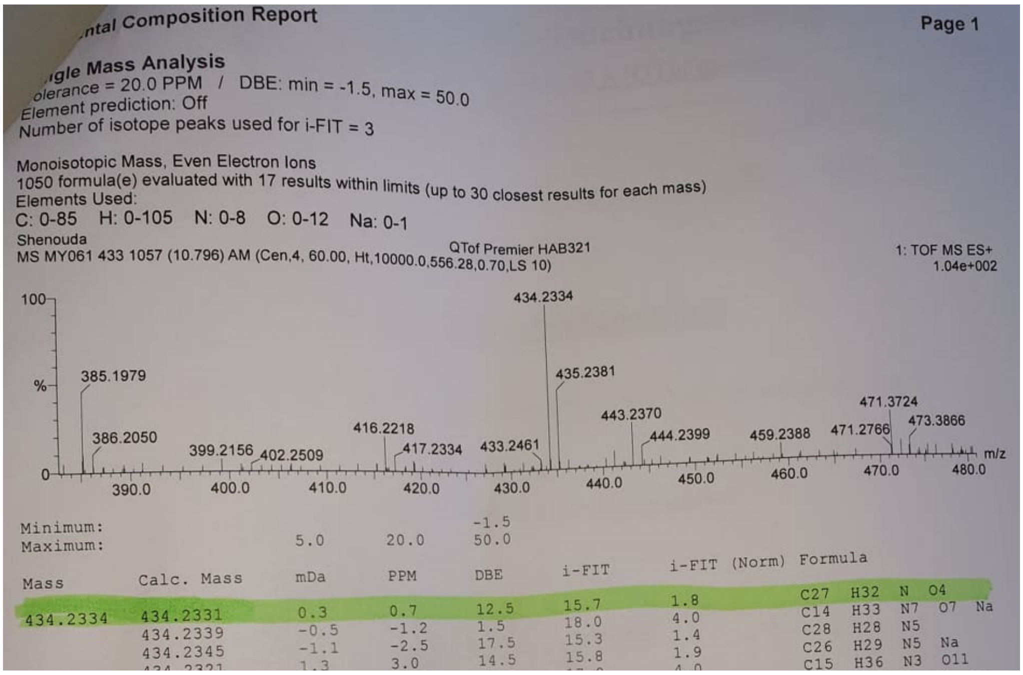

Figure S9.5. HRMS analysis of compound **16**.

10. References

1. Jin, F.J.; Maruyama, J.; Juvvadi, P.R.; Arioka, M.; Kitamoto, K. Development of a Novel Quadruple Auxotrophic Host Transformation System by ArgB Gene Disruption Using AdeA Gene and Exploiting Adenine Auxotrophy in *Aspergillus oryzae*. *FEMS Microbiol Lett* **2004**, *239*, 79–85, doi:10.1016/j.femsle.2004.08.025.
2. Pahirulzaman KA, Williams K, Lazarus CM. A toolkit for heterologous expression of metabolic pathways in *Aspergillus oryzae*. *Methods Enzymol*. 2012;517:241-60. doi: 10.1016/B978-0-12-404634-4.00012-7. PMID: 23084942.
3. Derntl C, Kiesenhofer DP, Mach RL, Mach-Aigner AR. Novel Strategies for Genomic Manipulation of *Trichoderma reesei* with the Purpose of Strain Engineering [published correction appears in *Appl Environ Microbiol*. 2017 Oct 31;83(22):]. *Appl Environ Microbiol*. 2015; 81(18):6314-6323. doi:10.1128/AEM.01545-15
4. Gietz, R. D.; Schiestl, R. H. High-Efficiency Yeast Transformation Using the LiAc/SS Carrier DNA/PEG Method, *Nature Protoc.*, **2007**, *2*, 31–34, doi:10.1038/nprot.2007.13.
5. Gilchrist, C.L.; Chooi, Y.H. Synthaser: A CD-Search Enabled Python Toolkit for Analysing Domain Architecture of Fungal Secondary Metabolite Megasynth(Et)Ases and Beyond. **2021**, doi:10.21203/rs.3.rs-850498/v1.
6. S. F. Altschul, T. L. Madden, A. A. Schäffer, J. Zhang, Z. Zhang, W. Miller and D. J. Lipman, *Nucleic Acids Res.*, 1997, **25**, 3389–3402.
7. Kahlert, L.; Bassiony, E.F.; Cox, R.J.; Skellam, E.J. Diels-Alder Reactions During the Biosynthesis of Sorbicillinoids. *Angewandte Chemie Int Ed Engl* **2020**, *59*, 5816–5822, doi:10.1002/anie.201915486.
8. Derntl, C.; Guzmán-Chávez, F.; Mello-de-Sousa, T.M.; Busse, H.-J.; Driessen, A.J.; Mach, R.L.; Mach-Aigner, A.R. In Vivo Study of the Sorbicillinoid Gene Cluster in Trichoderma Reesei. *Frontiers in Microbiology* **2017**, *08*, 2037, doi:10.3389/fmicb.2017.02037.
9. Baker, S.E.; Kroken, S.; Inderbitzin, P.; Asvarak, T.; Li, B.-Y.; Shi, L.; Yoder, O.C.; Turgeon, B.G. Two Polyketide Synthase-Encoding Genes Are Required for Biosynthesis of the Polyketide Virulence Factor, T-Toxin, by Cochliobolus Heterostrophus. *Mol Plant-microbe Interactions* **2006**, *19*, 139–149, doi:10.1094/mpmi-19-0139.
10. Brown, D.W.; Butchko, R.; Busman, M.; Proctor, R.H. Identification of Gene Clusters Associated with Fusaric Acid, Fusarin, and Perithecial Pigment Production in F*usarium Verticillioides*. *Fungal Genetics and Biology* **2012**, *49*, 521–532, doi:10.1016/j.fgb.2012.05.010.

11. Fujii, I.; Yoshida, N.; Shimomaki, S.; Oikawa, H.; Ebizuka, Y. An Iterative Type I Polyketide Synthase PKSN Catalyzes Synthesis of the Decaketide Alternapyrone with Regio-Specific Octa-Methylation. *Chem Biol* **2005**, *12*, 1301–1309, doi:10.1016/j.chembiol.2005.09.015.
12. Wang, X.; Gao, Y.-L.; Zhang, M.-L.; Zhang, H.-D.; Huang, J.-Z.; Li, L. Genome Mining and Biosynthesis of the Acyl-CoA:Cholesterol Acyltransferase Inhibitor Beauveriolide I and III in Cordyceps Militaris. *J Biotechnol* **2020**, *309*, 85–91, doi:10.1016/j.jbiotec.2020.01.002.
13. Bai, T.; Quan, Z.; Zhai, R.; Awakawa, T.; Matsuda, Y.; Abe, I. Elucidation and Heterologous Reconstitution of Chrodrimanin B Biosynthesis. *Organic Letters* **2018**, doi:10.1021/acs.orglett.8b03268.
14. Gallo, A.; Knox, B.P.; Bruno, K.S.; Solfrizzo, M.; Baker, S.E.; Perrone, G. Identification and Characterization of the Polyketide Synthase Involved in Ochratoxin A Biosynthesis in *Aspergillus Carbonarius*. *Int J Food Microbiol* **2014**, *179*, 10–17, doi:10.1016/j.ijfoodmicro.2014.03.013.
15. Wang, S.; Xu, Y.; Maine, E.A.; Wijeratne, E.M.K.; Espinosa-Artiles, P.; Gunatilaka, A.A.L.; Molnár, I. Functional Characterization of the Biosynthesis of Radicicol, an Hsp90 Inhibitor Resorcylic Acid Lactone from *Chaetomium Chiversi*i. *Chem Biol* **2008**, *15*, 1328–1338, doi:10.1016/j.chembiol.2008.10.006.
16. Lin, X.; Yuan, S.; Chen, S.; Chen, B.; Xu, H.; Liu, L.; Li, H.; Gao, Z. Heterologous Expression of Ilicicolin H Biosynthetic Gene Cluster and Production of a New Potent Antifungal Reagent, Ilicicolin J. *Molecules* **2019**, *24*, 2267, doi:10.3390/molecules24122267.
17. Liu, L.; Tang, M.-C.; Tang, Y. Fungal Highly Reducing Polyketide Synthases Biosynthesize Salicylaldehydes That Are Precursors to Epoxycyclohexenol Natural Products. *J Am Chem Soc* **2019**, *141*, 19538–19541, doi:10.1021/jacs.9b09669.
18. Zeng, G.; Zhang, P.; Zhang, Q.; Zhao, H.; Li, Z.; Zhang, X.; Wang, C.; Yin, W.-B.; Fang, W. Duplication of a Pks Gene Cluster and Subsequent Functional Diversification Facilitate Environmental Adaptation in *Metarhizium* Species. *Plos Genet* **2018**, *14*, e1007472, doi:10.1371/journal.pgen.1007472.
19. Ahuja, M.; Chiang, Y.-M.; Chang, S.-L.; Praseuth, M.B.; Entwistle, R.; Sanchez, J.F.; Lo, H.-C.; Yeh, H.-H.; Oakley, B.R.; Wang, C.C. Illuminating the Diversity of Aromatic Polyketide Synthases In *Aspergillus Nidulan*s. *Journal of the American Chemical Society* **2012**, *134*, 8212–8221, doi:10.1021/ja3016395.
20. Araki, Y.; Awakawa, T.; Matsuzaki, M.; Cho, R.; Matsuda, Y.; Hoshino, S.; Shinohara, Y.; Yamamoto, M.; Kido, Y.; Inaoka, D.; et al. Complete Biosynthetic Pathways of Ascofuranone and Ascochlorin in Acremonium Egyptiacum. *Proceedings of the National Academy of Sciences* **2019**, 201819254, doi:10.1073/pnas.1819254116.
21. Gilchrist, C.L.M.; Booth, T.J.; Wersch, B. van; Grieken, L. van; Medema, M.H.; Chooi, Y.-H. Cblaster: A Remote Search Tool for Rapid Identification and Visualization of Homologous Gene Clusters. *Bioinform Adv* **2021**, *1*, vbab016, doi:10.1093/bioadv/vbab016.
22. Rush, T.A.; Shrestha, H.K.; Meena, M.G.; Spangler, M.K.; Ellis, J.C.; Labbé, J.L.; Abraham, P.E. Bioprospecting Trichoderma: A Systematic Roadmap to Screen Genomes and Natural Products for Biocontrol Applications. *Frontiers Fungal Biology* **2021**, *2*, 716511, doi:10.3389/ffunb.2021.716511.
23. Zhang, Z.; Jamieson, C.S.; Zhao, Y.-L.; Li, D.; Ohashi, M.; Houk, K.N.; Tang, Y. Enzyme-Catalyzed Inverse-Electron Demand Diels–Alder Reaction in the Biosynthesis of Antifungal Ilicicolin H. *J Am Chem Soc* **2019**, *141*, 5659–5663, doi:10.1021/jacs.9b02204.
